# Supplementary material for: Neural general circulation models for weather and climate
Source: Nature. 2024 Jul 22;632(8027):1060–6. doi: 10.1038/s41586-024-07744-y (PMC11357988; doi:10.1038/s41586-024-07744-y)
Supplement: Supplementary file 1 — Supplementary Information (38 figures, 6 tables): (A) Lines of code in atmospheric models; (B) Dynamical core of NeuralGCM; (C) Learned physics of NeuralGCM; (D) Encoder and decoder of NeuralGCM; (E) Time integration; (F) Evaluation metrics; (G) Training; (H) Additional weather evaluations; (I) Additional climate evaluations. [file 41586_2024_7744_MOESM1_ESM.pdf]

nature portfolio

https://doi.org/10.1038/s41586-024-07744-y

Supplementary information

Neural general circulation models for weather and climate

In the format provided by the authors and unedited

Neural General Circulation Models for Weather and Climate - Supplementary Information

Dmitrii Kochkov[1*†], Janni Yuval[1*†], Ian Langmore[1†], Peter Norgaard[1†], Jamie Smith[1†], Griffin Mooers[1], Milan Klöwer[4], James Lottes[1], Stephan Rasp[1], Peter Düben[3], Sam Hatfield[3], Peter Battaglia[2], Alvaro Sanchez-Gonzalez[2], Matthew Willson[2], Michael P. Brenner[1,5], Stephan Hoyer[1*†]

[1]Google Research, Mountain View, CA.
[2]Google DeepMind, London, UK.
[3]European Centre for Medium-Range Weather Forecasts, Reading, UK.
[4]Earth, Atmospheric and Planetary Sciences, Massachusetts Institute of Technology.
[5]School of Engineering and Applied Sciences, Harvard University.

*Corresponding author(s). E-mail(s): dkochkov@google.com; janniyuval@google.com; shoyer@google.com;
†These authors contributed equally to this work.

Appendices

A Lines of code in atmospheric models 4
A.1 fv3atm . . . 4
A.2 GraphCast . . . 4
A.3 NeuralGCM . . . 4

B Dynamical core of NeuralGCM 5
B.1 Discretization of the dynamical core . . . 5
B.2 Primitive equations . . . 6
B.3 Numerics . . . 7

C Learned physics of NeuralGCM 8
C.1 Input features for all models . . . 9

C.2 Additional input features for stochastic models . . . 9
C.3 Normalization of input features . . . 10
C.4 Network architecture . . . 10
C.4.1 Vertical embedding network . . . 11
C.4.2 Surface embedding network . . . 12
C.5 Network output scaling . . . 12
C.6 Interpretability of learned physics tendencies . . . 12

D Encoder and Decoder of NeuralGCM **14**
D.1 Encoder . . . 15
D.2 Decoder . . . 15

E Time integration **18**
E.1 Time integration scheme . . . 18
E.2 Filtering . . . 18

F Evaluation metrics **20**
F.1 Root mean square error (RMSE) . . . 20
F.2 Root mean squared bias (RMSB) . . . 21
F.3 Continuous Ranked Probability Score (CRPS) . . . 21
F.4 Spread-Skill ratio . . . 22

G Training **23**
G.1 Optimizer settings . . . 23
G.2 Training data and unroll schedules . . . 24
G.3 Variable rescaling for losses . . . 24
G.4 Loss for deterministic models . . . 25
G.4.1 Accuracy loss: filtered MSE . . . 25
G.4.2 Sharpness loss: spectrum MSE . . . 26
G.4.3 Bias loss: spectral bias MSE . . . 26
G.5 Decoder fine-tuning . . . 27
G.6 Loss for stochastic models . . . 27
G.7 Training resources . . . 28

H Additional weather evaluations **30**
H.1 Accuracy . . . 30
H.2 Derived variables and spectra . . . 39
H.3 Visualization of ensemble weather forecasts . . . 40
H.4 Evaluation of lower resolution models . . . 43
H.5 Diagnosing precipitation minus evaporation . . . 43
H.6 Ablation tests . . . 47
H.6.1 Different loss functions . . . 47
H.6.2 Training on shorter rollouts . . . 48
H.6.3 Learning curve - pure ML vs hybrid . . . 48
H.7 Instability case study . . . 50

I Additional climate evaluations **54**

I.1 Seasonal cycle 54
I.2 Tropical cyclone tracking 55
I.3 CMIP6 models used in AMIP runs 59
I.4 Generalizing to unseen data 63
I.4.1 Weather forecasting in warmer years 63
I.4.2 Extrapolation to warmer climates 64

A Lines of code in atmospheric models

To measure of the complexity of different code-bases, we counted the number of “core model” lines of code, excluding files devoted to tests, examples, input/output, coupling between different frameworks and running models.

A.1 fv3atm

We counted a total of 376 578 lines of core model code for fv3atm, the atmospheric component of the NOAA’s Unified Forecast System (UFS) weather model, which we downloaded from https://github.com/NOAA-EMC/fv3atm on 7 November, 2023.

Excluding files with “test” or “example” in their paths, we counted the following number of lines in Fortran files in the two major modules of fv3atm (ending in .f or .f90):

1. 42 387 lines in the FV3 dynamical core (atmos_cubed_sphere/model)
2. 334 191 lines in the Common Community Physics Package (CCPP) Physics (ccpp/physics/physics)

A.2 GraphCast

We counted a total of 5417 lines of core model code for GraphCast [1], which we downloaded from https://github.com/deepmind/graphcast on 7 November, 2023.

A.3 NeuralGCM

We counted a total of 20 257 lines of core model code for NeuralGCM, split between two major modules:

1. 9628 lines in the spectral dynamical core, available at https://github.com/google-research/dinosaur
2. 10 629 lines in the machine learning code, available at https://github.com/google-research/neuralgcm

B Dynamical core of NeuralGCM

The dynamical core provides NeuralGCM with strong physics priors based on well understood and easy to simulate phenomena. In section B.1 we provide more details on spatial discretization of the atmospheric state in NeuralGCM. In section B.2 we summarize the governing equations of the dynamical core. In section B.3 we provide references to numerical implementations and rationale for our choices.

B.1 Discretization of the dynamical core

Our dynamical core uses a Gaussian grid and sigma coordinates [2] to discretize the computational domain. Gaussian grids enable fast and accurate transformations between the grid space representation and spherical harmonics basis. They result in equiangular longitude lines and unequal spacing latitudes defined by the Gaussian quadrature. Terrain-following sigma coordinates discretize the vertical direction by the fraction of the surface pressure, and thus correspond to non-stationary vertical height since surface pressure changes with time. Cell boundaries in sigma coordinates take values $\sigma \in [0, 1]$, with $\sigma = 0$ corresponding to the top of the atmosphere ($p = 0$ pressure boundary) and $\sigma = 1$ representing the earth's surface.

In this work we have trained a lineup of models that make forecasts at varying horizontal resolutions: 2.8°, 1.4°, and 0.7°, corresponding to truncated linear Gaussian grids TL63, TL127, TL255. The number in the grid name corresponds to the maximum total wavenumber of spherical harmonic that the grid can represent. These grids provide a framework for transforming data from grid space (nodal) to spherical harmonic representations with minimal loss of information. When solving model equations we use cubic truncation Gaussian grids T62, T125 and T253, that capture a similar number of spherical harmonics, while avoiding aliasing errors and minimizing the need to increase array dimensions above a multiple of 128, which is expensive on the Google TPU. See SI Table 1 for resolution details. All models use 32 equidistant sigma levels for vertical discretization. We suspect that using higher vertical resolution with assimilation data from more levels could further improve the performance.

Grid name	Longitude nodes	Latitude nodes	Max total wavenumber
TL63	128	64	63
TL127	256	128	127
TL255	512	256	255
T62	190	95	62
T125	379	190	125
T254	766	383	254

Supplementary Table 1: Spatial and spectral resolutions of horizontal grids used by NeuralGCM.

B.2 Primitive equations

The dynamical core of NeuralGCM solves the primitive equations, which represent a combination of (1) momentum equations, (2) the second law of thermodynamics, (3) a thermodynamic equation of state (ideal gas), (4) continuity equation and (5) hydrostatic approximation. For solving the equations we use a divergence-vorticity representation of the horizontal winds, resulting in equations for the following seven prognostic variables: divergence δ, vorticity ζ, temperature T, logarithm of the surface pressure $\log p_s$, as well as 3 moisture species (specific humidity q, specific cloud ice q_{c_i} and specific liquid cloud water content q_{c_l}). To facilitate efficient time integration of our models we split temperature T into a uniform reference temperature on each sigma level $\bar{T}_\sigma$ and temperature deviations per level $T'_\sigma = T_\sigma - \bar{T}_\sigma$. The resulting equations are:

$$\begin{aligned}
\frac{\partial \zeta}{\partial t} &= -\nabla \times \left((\zeta + f)\mathbf{k} \times \mathbf{u} + \dot{\sigma}\frac{\partial \mathbf{u}}{\partial \sigma} + RT'\nabla \log p_s \right) \\
\frac{\partial \delta}{\partial t} &= -\nabla \cdot \left((\zeta + f)\mathbf{k} \times \mathbf{u} + \dot{\sigma}\frac{\partial \mathbf{u}}{\partial \sigma} + RT'\nabla \log p_s \right) - \nabla^2 \left(\frac{||\mathbf{u}||^2}{2} + \Phi + R\bar{T}\log p_s \right) \\
\frac{\partial T}{\partial t} &= -\mathbf{u} \cdot \nabla T - \dot{\sigma}\frac{\partial T}{\partial \sigma} + \frac{\kappa T \omega}{p} = -\nabla \cdot \mathbf{u}T' + T'\delta - \dot{\sigma}\frac{\partial T}{\partial \sigma} + \frac{\kappa T \omega}{p} \\
\frac{\partial q_i}{\partial t} &= -\nabla \cdot \mathbf{u}q_i + q_i\delta - \dot{\sigma}\frac{\partial q_i}{\partial \sigma} \\
\frac{\partial \log p_s}{\partial t} &= -\frac{1}{p_s}\int_0^1 \nabla \cdot (\mathbf{u}p_s)\, d\sigma = -\int_0^1 (\delta + \mathbf{u} \cdot \nabla \log p_s)\, d\sigma
\end{aligned} \tag{1}$$

with horizontal velocity vector $\mathbf{u} = \nabla(\Delta^{-1}\delta) + \mathbf{k} \times \nabla(\Delta^{-1}\zeta)$, Coriolis parameter f, upward-directed unit vector parallel to the z-axis $\mathbf{k}$, ideal gas constant R, heat capacity at constant pressure C_p, $\kappa = \frac{R}{C_p}$, diagnosed vertical velocity in sigma coordinates $\dot{\sigma}$, diagnosed change in pressure of a fluid parcel $\omega \equiv \frac{dp}{dt}$, diagnosed geopotential Φ, diagnosed virtual temperature T_ν and each moisture species denoted as q_i.

Diagnostic quantities are computed as follows:

$$\dot{\sigma}_{k+\frac{1}{2}} = -\sigma_{k+\frac{1}{2}}\frac{\partial \log p_s}{\partial t} - \frac{1}{p_s}\int_0^{\sigma_{k+\frac{1}{2}}} \nabla \cdot (p_s\mathbf{u})\, d\sigma \tag{2}$$

$$\frac{\omega_k}{p_s\sigma_k} = \mathbf{u}_k \cdot \nabla \log p_s - \frac{1}{\sigma_k}\int_0^{\sigma_k} (\delta + \mathbf{u} \cdot \nabla \log p_s)\, d\sigma \tag{3}$$

$$\Phi_k = \Phi_s + R\int_{\log \sigma_k}^{0} T_\nu\, d\log \sigma \tag{4}$$

$$T_\nu = T(1 + \left(\frac{R_{vap}}{R} - 1\right) q - q_{c_i} - q_{c_l}) \tag{5}$$

where $\Phi_s = gz_s$ is the geopotential at the surface.

B.3 Numerics

Our choice of the numerical schemes for interpolation, integrals and diagnostics exactly follows Durran's book [3] §8.6, with the addition of moisture species (which are advected by the wind and only affect the dynamics through through their effect on the virtual temperature). We use semi-implicit time-integration scheme, where all right hand side terms are separated into groups that are treated either explicitly or implicitly. This avoids severe time step limitations due to fast moving gravity waves.

Our choice of dynamical core was also informed by our desire to run efficiently on machine learning accelerators, in particular Google TPUs [4]. TPUs have dedicated hardware for low-precision matrix-matrix multiplication, which conveniently is well suited for the bottleneck in spectral methods, which are forward and inverse spherical harmonic transformations. Accordingly, we use single-precision arithmetic throughout. We found that full single precision for spherical harmonic transformations was not required to obtain accurate results even on our largest grid sizes, and according use only three passes of bfloat16 matrix-multiplication rather than the six passes that would required for full single precision [5]. Our implementation supports parallelism across spatial dimensions (x, y, and z) for running on multiple accelerator cores, using XLA SPMD [6], with JAX's `shard_map` for parallelizing key bottlenecks including matrix-multiplications in spherical harmonic transforms [7].

C Learned physics of NeuralGCM

In NeuralGCM, effects of physical processes not accounted for by the dynamical core, as well as computational errors within the dynamical core, are approximated by neural networks. Associated tendencies of the atmospheric state are computed in two steps: (1) features extraction and normalization (sections C.1, C.3) (2) neural network forward pass and rescaling (sections C.4, C.5).

The overall data flow diagram, with the exclusion of feature normalization steps, is shown in SI Fig. 1. To describe the initial value problem solved by each NeuralGCM forecast, we let $Y(t)$ be the ERA5 state at time t, $X(t)$ the (decoded) NeuralGCM state, and $\tilde{X}(t)$ the encoded state. The network tendencies $\Psi(\tilde{X})$ are added to $\Phi(\tilde{X})$, the right hand side of the primitive equations (1) in encoded space. In other words, NeuralGCM implements

$$\frac{\partial \tilde{X}}{\partial t} = \Phi(\tilde{X}) + \Psi(\tilde{X}), \quad t_0 < t < t_0 + \tau,$$
$$\tilde{X}(t_0) = \mathrm{Encode}(Y(t_0)).$$

Then finally, $X = \mathrm{Decode}(\tilde{X})$ is evaluated against Y.

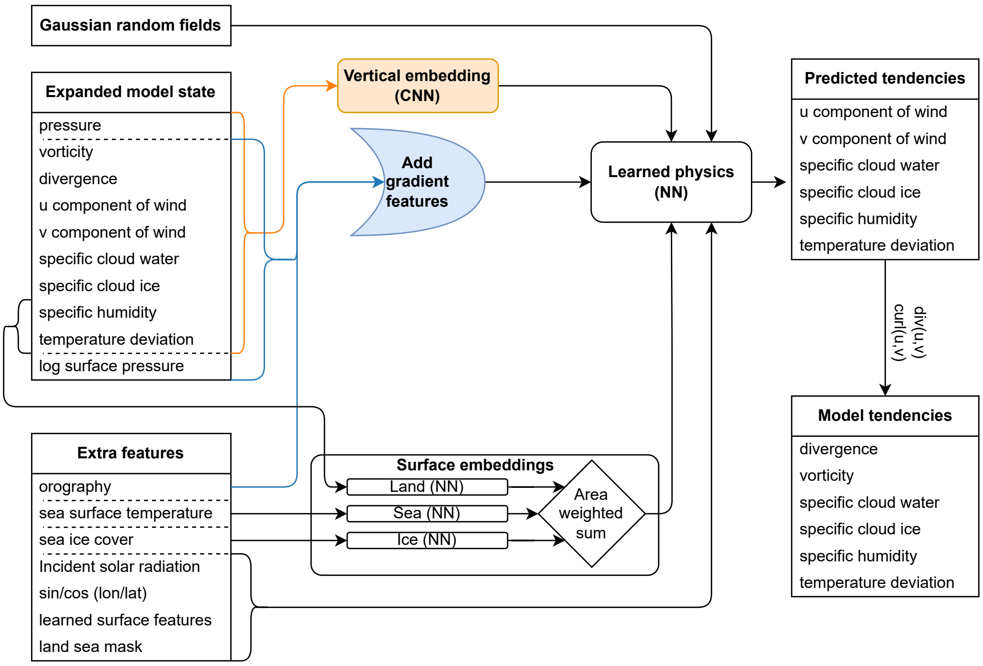

Supplementary Figure 1: Visualization of the data flow in the learned physics module of NeuralGCM.

C.1 Input features for all models

The core input features to the neural network include the vertical structure of the divergence, vorticity, wind vector, temperature deviation, specific humidity, specific cloud ice water content, specific cloud liquid water content and (log) surface pressure.

Additionally, our model incorporates various supplementary features to capture critical information relevant to the atmospheric conditions. These features include spatial derivatives of the core features (∂_ϕ, ∂_θ and ∇^2), a land-sea mask, incoming solar radiation, orography (along with spatial gradients), cosine, and sine of the latitude, pressure levels (i.e., sigma level times the pressure surface), and an 8-dimensional (for NeuralGCM-2.8° and NeuralGCM-ENS) or 32-dimensionanl (for NeuralGCM-0.7° and NeuralGCM-1.4°) location-specific embedding vector for each horizontal grid-point. This embedding vector aims to represent static location-specific information. It is initialized to random values and optimized during training.

We use two embedding modules in NeuralGCM that aim to extract helpful representations that are used as input features by the main network described in C.4. Both are computed by small neural networks that use the same weights shared across all spatial locations to promote feature learning. Each embedding module takes a restricted set of inputs. Vertical embedding network C.4.1 aims to extract common features across the vertical structure of the atmosphere. Surface embedding network C.4.2 aims to estimate the state of the atmosphere's surface boundary. To accomplish this, it receives additional surface-related inputs, in particular sea surface temperature (SST) and sea ice concentration.

When running weather forecast evaluation the provided SST and sea ice concentration remain constant throughout each forecast, with values taken from the day before the initial time of the forecast launch. This approach ensures that the model relies only on data available at the forecast initialization.

When running seasonal and climate evaluation forecasts, we do prescribe the SST and sea ice concentration from ERA5 data and update them at 6-hour intervals for the 2.8° resolution model and 12-hour intervals for the 1.4° resolution model (details on how land surfaces were used can be found in C.4.2). This simulates a one way coupling to an ocean model that follows historical evolution.

In some NeuralGCM variations we experimented with adding core features from the previous time step as additional inputs. We did not find this modification to have any significant effect on model performance.

C.2 Additional input features for stochastic models

In stochastic models, the encoder and forward step (but not decoder) each make use of ten additional space-time correlated Gaussian Random Fields (GRF), for a total of twenty fields. All fields are independent of each other. The encoder's fields are used only once in each simulation, and the forward step's fields evolve independently of the model state. Every forecast uses a different seed to form new, independent GRFs. This provides sources of randomness needed to generate an ensemble of statistically independent forecasts.

Each GRF is constructed in a spherical harmonic basis, with adjustable length scale λ and time scale γ.

$$Z(t) = \sum_{l,m} Z_{l,m}(t) Y_{l,m},$$

where $Y_{l,m}$ are the spherical harmonics, and $Z_{l,m}$ are scalar Gaussian processes satisfying (with R_e the earth's radius)

$$\mathbb{E}\left[Z_{l,m}(t)\right] \equiv 0,$$
$$\mathbb{E}\left[Z_{l,m}(t) Z_{l',m'}(t')\right] = \begin{cases} F_0 \exp\left\{-\left(\frac{\lambda}{R_e}\right)^2 \frac{l(l+1)}{2}\right\} \exp\{-|t-t'|/\gamma\}, & l = l', m = m' \\ 0 & \text{otherwise.} \end{cases}$$

The normalization constant F_0 is chosen so that the global mean variance is 1. This is the same construction used by ECMWF in [8]. In training NeuralGCM-ENS, the fields are initialized with length scales ranging from 85km to $10,000$km and time scales from 30 minutes to 60 hours. These values were fixed for the first 1000 training iterations and optimized afterwards. SI Fig. 2 shows parameter values during training. Most changed very little. Some parameters became constant over space/time (still with variance $= 1$). We interpret them as representing random model parameters, but more investigation is needed.

C.3 Normalization of input features

We normalize all input features to be approximately distributed with zero mean and unit variance to improve training dynamics. We do so by estimating the mean and the standard deviation of all of the features at initialization and adding appropriate shift and rescale transformations before feeding features into the neural network. All features were normalized uniformly across all atmospheric levels, except for specific humidity q which is normalized per-level. Features corresponding to trainable parameters (termed learned features in section C.1), Gaussian random field features (section C.2) and embedding features were skipped during normalization as they are normally distributed by construction.

C.4 Network architecture

For our fully-connected neural networks with residual connections, we use a Encode-Process-Decode (EPD) architecture [9] with 5 fully connected MLP blocks in the "Process" component. All input features are concatenated together before being passed to the "Encode" layer, which is a linear layer that maps input features to a latent vector of size 384. All "Process" blocks use 3-layer MLP block with 384 hidden units to compute updates to the latent vector. Finally, linear "Decode" layer maps the vector of size 384 to the output vector, which is is then split into per-level values for different variables. See extended data table 1 for a count of learnable parameters by model. NeuralGCM models differ in parameter count primarily due to the location-specific

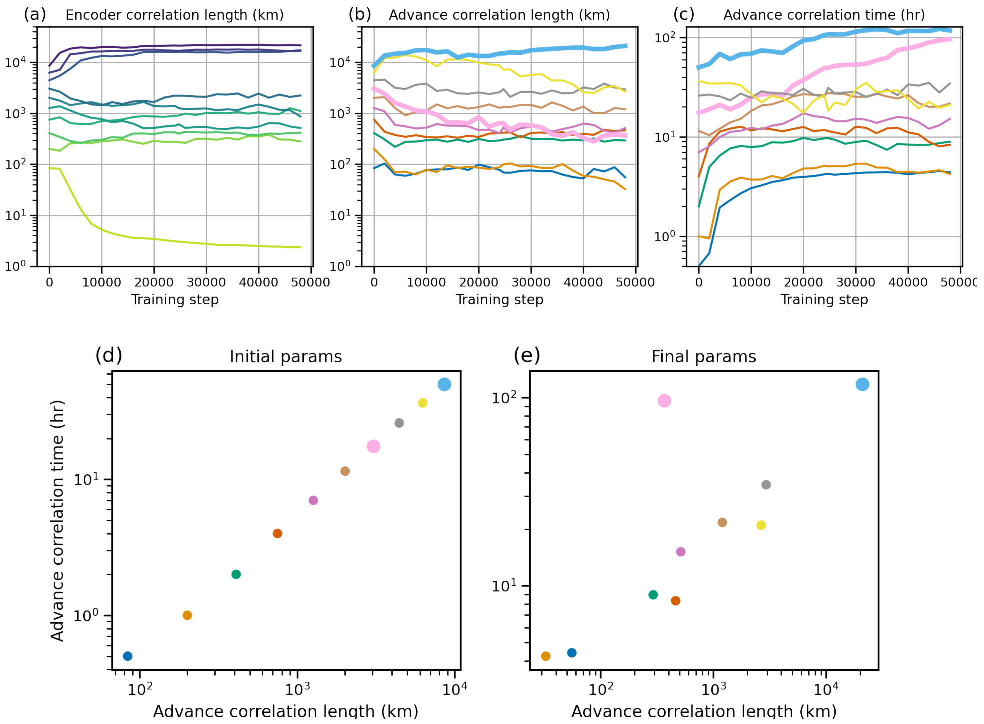

Supplementary Figure 2: Random field parameters evolution during training. (a) Shows encoder field correlation lengths changing during training. The advance step's fields evolution during training are shown in (b) and (c). A scatter plot of before (d) and after training (e) is also shown. The two outlier fields with correlation time ≈ 100 hours are of interest (thicker lines/dots). Since lead time during training is at most 120 hours, the model preferred these fields to be fixed in time.

embedding vector assigned to each horizontal grid point. Higher-resolution models require more parameters. Additionally, NeuralGCM-1.4° and NeuralGCM-0.7° models use 32-length embeddings, while NeuralGCM-ENS and NeuralGCM-2.8° models use 8-length embeddings.

In all models we predict tendencies of the wind vector, temperature and moisture species at all levels.

C.4.1 Vertical embedding network

We use 1D convolutional network to compute vertical embeddings. We use 5 layers with 64 hidden and 32 output channels. Input channels correspond to the 8 atmospheric variables (divergence, vorticity, two components of the wind vector, temperature and three moisture species), aligned by their vertical location in the atmosphere.

C.4.2 Surface embedding network

The surface embeddings are calculated by aggregating outputs from three different embeddings corresponding to distinct surface types: land, sea, and sea ice. The "sea embedding" is a neural network that receives the sea surface temperature (SST) as input. The settings for the "land embedding" and "sea ice embedding" vary slightly across different models. For the deterministic models with resolutions of 1.4° and 0.7°, we employ a constant learned embedding for both land embedding and sea ice embedding. Conversely, for the 2.8° resolution deterministic model and the 1.4° ensemble model, we use neural networks that take the lowest-level atmospheric temperature and moisture as inputs. The output from each network is an embedding of size 8. Each of these smaller networks is a fully connected network comprising three hidden layers with 16 hidden neurons, followed by a readout linear layer that produces an embedding of size 8. The aggregation step is determined based on the relative fraction of each surface type at each grid point.

C.5 Network output scaling

When predicting learned physics tendencies, the outputs of the neural network are scaled by 0.01 standard deviation of the tendencies for each variable, which are estimated from ERA5 data using finite-difference method at 1-hour intervals. The standard deviation is estimated using 10 snapshots averaged over the globe.

C.6 Interpretability of learned physics tendencies

In SI Fig. 3 we show a comparison of tendencies associated with the dynamical core and trained learned physics component of NeuralGCM. While current instantiation of NeuralGCM does not allow for separation of the tendencies associated with different physical processes, we find several physically plausible signatures. In particular, near surface temperature tendencies shown in SI Fig. 3 (a) resemble surface heating region (where land is cooled during night time and is heated during daytime). In SI Fig. 3 (b) we find significant warming signal in the upper atmosphere, potentially associated with convective processes. SI Fig. 3 (c) show that learned physics generally counteracts the wind tendencies that of the dynamical core, potentially representing drag effects, which are not found further away from the surface (SI Fig. 3 (d)).

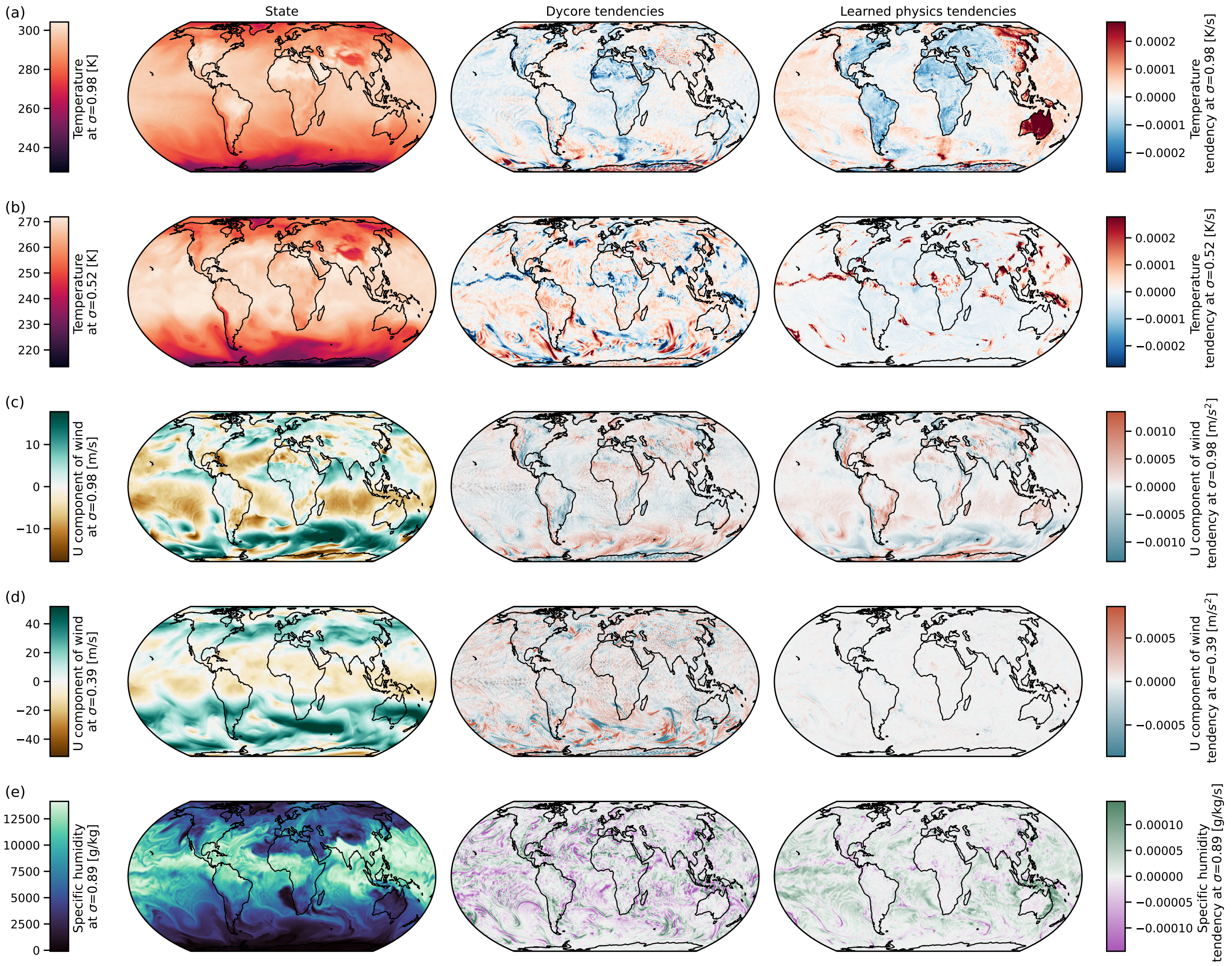

Supplementary Figure 3: Comparison on tendencies produced by the dynamical core and the learned physics component of NeuralGCM-0.7°. All tendencies are computed on 2020-08- 24T00z. The plot shows σ level slices of temperature, zonal component of wind and specific humidity, as well as tendencies associated with the dynamical core and learned physics.

D Encoder and Decoder of NeuralGCM

To interface NeuralGCM that uses sigma coordinate representation of the atmosphere with ERA5 data we use Encoder and Decoder modules. Each component is based on regridding between sigma and pressure levels combined with learned correction. The overall data flow for encoder and decoder components are shown in SI Fig. 4 (a) and (b) respectively.

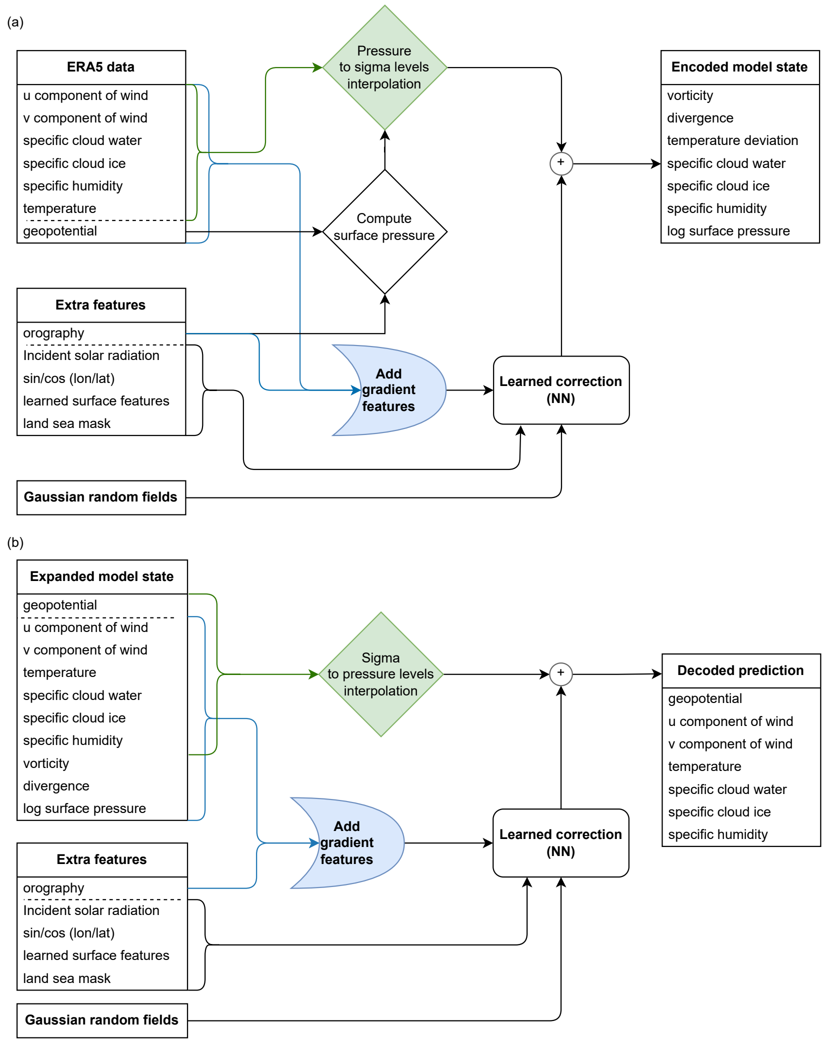

Supplementary Figure 4: Visualization of the data flow in encoder and decoder modules of NeuralGCM.

D.1 Encoder

We use Encoder to obtain a model state in sigma coordinates from an ERA5 snapshot on pressure coordinates in three steps. First, we compute surface pressure for each (longitude, latitude), by calculating the pressure levels at which geopotential field matches that of the surface. Next, we linearly interpolate all relevant atmospheric variables to NeuralGCM's sigma coordinates. Finally, we add a correction computed using a neural network of the same structure as in learned physics module (section C.4) to the interpolation results. The last step significantly alleviates the initialization shock, which otherwise contaminates forecasts with rapid oscillations.

When computing the correction, input features to the network are extracted from input data on pressure levels. We omit embedding features from the Encoder network inputs. Network outputs include corrections to divergence, vorticity, temperature, logarithm of the surface pressure and all moisture species. Before being combined with linear interpolation, network outputs are scaled by 0.02 standard deviation of corresponding variables. SI Fig. 5 compares NeuralGCM-0.7° model states (on vertical sigma levels) computed with encoder against using pressure to sigma level interpolation. As shown in SI Fig. 5 (a), the overall structure of the atmospheric state is hardly affected.

D.2 Decoder

We use Decoder to map model state on sigma coordinates back to ERA5 snapshot on pressure coordinates in three steps. First, we diagnose geopotential from temperature and moisture using Eq. 4. Next, we interpolate the results to pressure levels of ERA5. For pressure levels that correspond to values above the Earth's surface, we uses linear interpolation. To extrapolate data from sigma coordinates (which are terrain-following) to pressure coordinates (that extend below the Earth's surface) we use linear extrapolation for geopotential and temperature. For all other variables we uses boundary values for extrapolation. These choices aim to approximate a more complicated procedure used by ECMWF for pressure extrapolation. Finally, we add a correction computed using a neural network of the same structure as in learned physics module (section C.4) to the interpolation results.

Similarly to the Encoder, embedding features are omitted. Network outputs include corrections to the horizontal wind vector, temperature, geopotential and all moisture species. Before being combined with the result of interpolation, network outputs are scaled by 0.02 standard deviation of corresponding variables. SI Fig. 6 compares NeuralGCM-0.7° predictions processed with decoder and those simply interpolated from sigma to pressure levels. SI Fig. 6 (a) shows that the overall structure of the atmospheric state is hardly affected, for the exception of a few minor artifacts around mountainous areas. SI Fig. 6 (b) shows that corrections introduced by the neural network mostly correspond to high pressure values that fall close to or below topographic features.

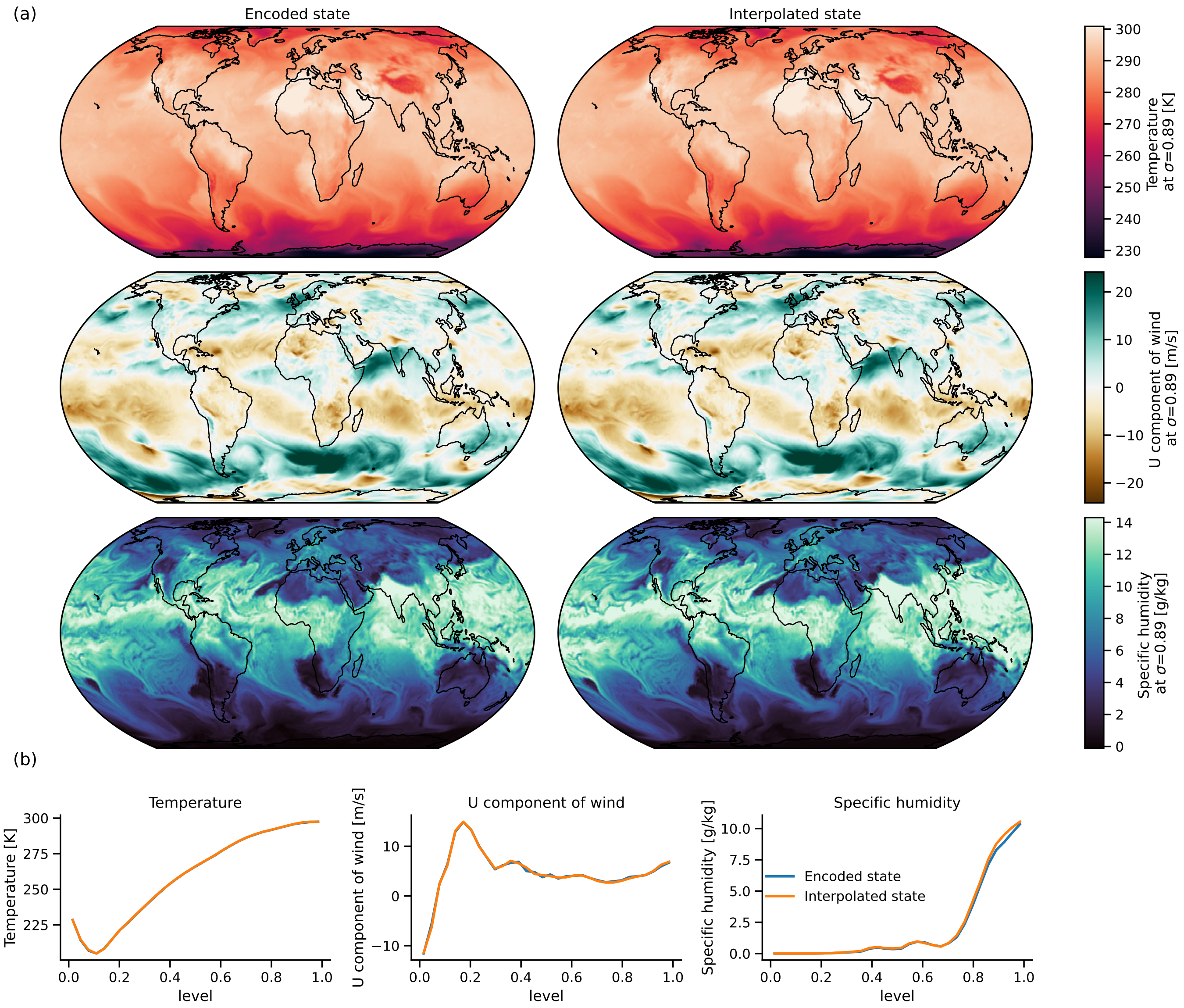

Supplementary Figure 5: Comparison of NeuralGCM-0.7° model states produced by the encoder and that of the pressure to sigma level interpolation scheme. (a) Visualization of temperature, u component of wind and specific humidity with full encoder and that with learned components disabled. (b) Vertical profiles for the same variables evaluated at (longitude≈ 238 and latitude≈ 37).

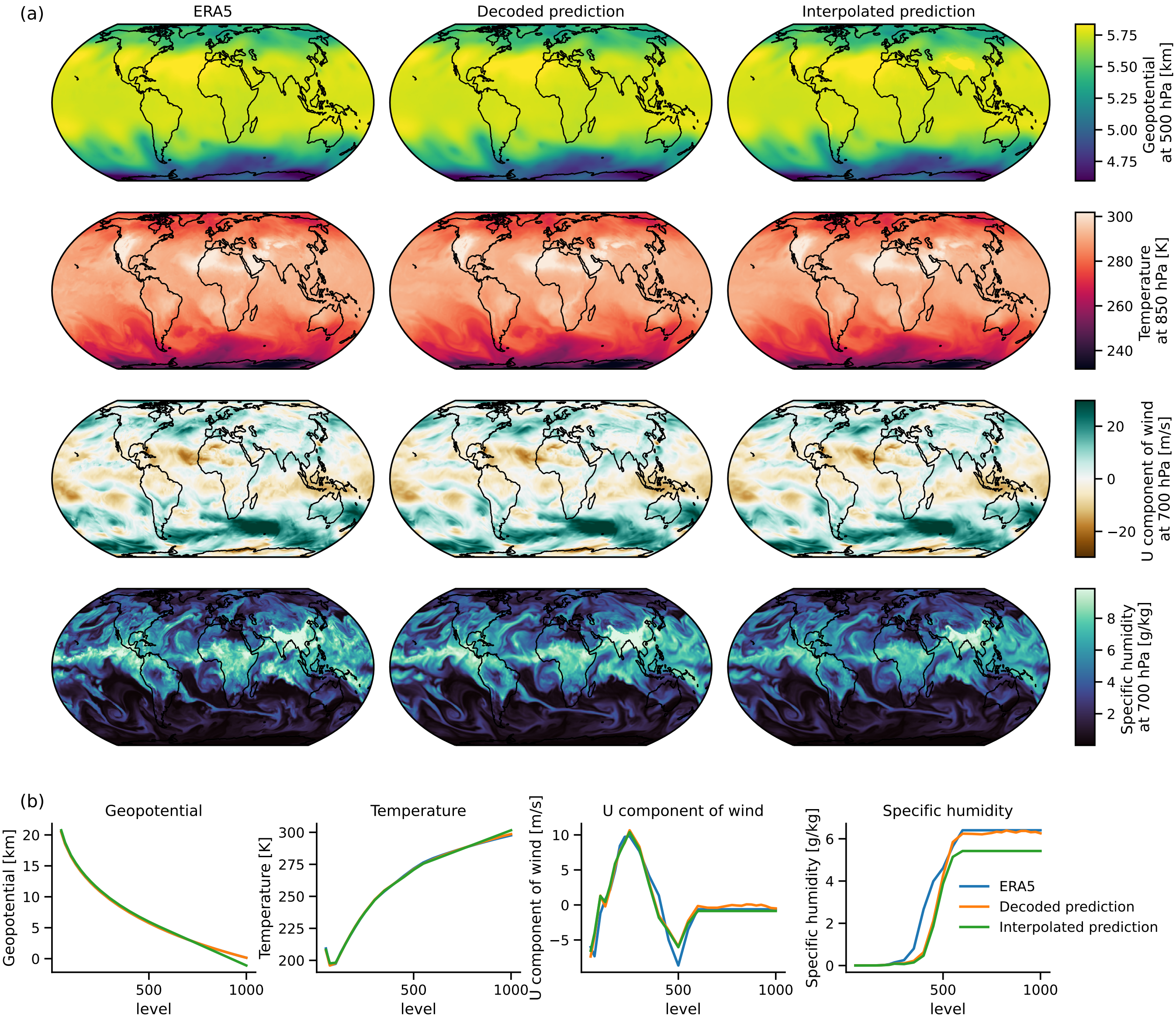

Supplementary Figure 6: Comparison of NeuralGCM decoder and underlying sigma level to pressure level interpolation scheme. (a) Visualization of geopotential, temperature, u component of wind and specific humidity from ERA5, 2 day NeuralGCM-0.7° prediction processed with NeuralGCM decoder and 2 day NeuralGCM-0.7° prediction interpolated from sigma to pressure levels without learned components of the decoder. (b) Vertical profiles for the same variables evaluated around Tibetan plateau (longitude$\approx$ 86 and latitude$\approx$ 32).

E Time integration

In NeuralGCM, the state of the atmosphere is advanced in time by integrating model equations that combine effects from the dynamical core and learned physics parameterizations. This is done iteratively using Implicit-Explicit integration scheme [10] described in E.1. Integration time step varies with resolution, as shown in SI Table 3. This results in iterative updates to the model state every 4-30 minutes, depending on model resolution. In contrast, data-driven methods commonly make predictions at 6-hour jumps [1, 11]. Throughout time integration, dynamical core tendencies are computed at every time step, while learned physics tendencies are only recomputed once every 60 minutes for our lowest resolution (2.8°) model and every 30 minutes for all others. This is done to avoid excessive backpropagation through the neural networks in learned physics. At higher resolutions it might be advantageous to include more frequent updates to learned physics tendencies to be able to account for short-time processes (rather than statistical effect that varies smoothly in time). Similar to traditional spectral GCMs we introduce spectral filters to improve numerical stability [12], which are described in E.2.

E.1 Time integration scheme

As is typical for atmospheric models, in NeuralGCM we use semi-implicit ODE solvers to the solve the primitive equations, by partitioning dynamical tendencies into "implicit" and "explicit" terms. "Implicit" tendencies include linear terms of Eq. 1 that give rise to the low amplitude, fast moving gravity waves. These terms are treated implicitly, allowing for longer stable time steps, while the rest of the terms are computed explicitly.

Rather than the traditional semi-implicit leapfrog method, we use implicit-explicit Runge-Kutta methods to avoid the complexity of keeping track of multiple time-steps and time-filtering required by the traditional semi-implicit leapfrog method. Specifically, we use the semi-implicit Lorenz three cycle scheme (SIL3), which was developed specifically for global spectral weather models [10].

1/3	1/3				1/3	1/6	1/6		
2/3	1/6	1/2			1/3	0	1/3		
1	1/2	−1/2	1		1	3/8	0	3/8	1/4
	1/2	−1/2	1	0		3/8	0	3/8	1/4

Supplementary Table 2: Butcher tableau for the IMEX SIL3 scheme.

E.2 Filtering

During time integration we use two exponential filters of different strengths ("hard" and "soft"). These filters correspond to hyper-diffusion, a standard component of spectral atmospheric models used to stabilize dynamics [13]. Each transform a scalar

field x_{hml} in spherical harmonic representation as:

$$x_{hml} \rightarrow x_{hml} * e^{-a\left(\frac{k-c}{1-c}\right)^{2p}} \tag{6}$$

with filter attenuation a, filter cutoff c, filter order p, and normalized total wavenumber $k \equiv \frac{l}{l_{max}}$.

Filter parameters used by different NeuralGCM models are summarized in SI Table 3, where filter attenuation is specified via attenuation time α and time step dt via $a = \frac{\alpha}{dt}$. Both hard and soft filters are applied to the model state at the end of each integration step. We additionally apply hard filter to the outputs of learned physics parameterizations to avoid injection of high frequency noise in each model step. The filtering strength sets the true length scale of the simulation, which is generally slightly larger than the grid spacing.

Model resolution	Time step [minutes]	Filter	Attenuation time [minutes]	Order	Cutoff
2.8°	12	hard	4	10	0.4
		soft	120	3	0.0
1.4°	6	hard	8	6	0.4
		soft	120	3	0.0
0.7°	3.75	hard	4	6	0.4
		soft	120	3	0.0

Supplementary Table 3: Time step and filtering parameters of NeuralGCM models.

F Evaluation metrics

Evaluation metrics compare forecasts X with ground truth Y. In most cases, Y is ERA5 reanalysis. However, for evaluating ECMWF-HRES and ECMWF-ENS forecasts, we use the lead time = 0 (or "analysis") from ECMWF-HRES. This prevents data-driven approaches from having an unfair advantage. All evaluations reported in the paper were done after regridding X and Y to 1.5°. We use the WeatherBench2 library [14] to standardize model evaluation. Our primary evaluation metrics include: root mean square error (RMSE), root mean squared bias (RSMB), continuous ranked probability score (CRPS) and skill-spread ratio.

F.1 Root mean square error (RMSE)

RMSE compares $Y(t+\tau)$, the ground truth at time $t+\tau$, with $X(t \to t+\tau)$, a forecast initialized at time t (to $Y(t)$), at lead time τ hours into the future. This is reported separately for each variable $v \in \mathcal{V}$ and pressure level p, but averaged over initial times.

$$\mathcal{E}_{RMSE}(\tau, v, p) := \sqrt{\frac{1}{|\mathcal{T}|}\sum_{t\in\mathcal{T}} \|X(t \to t+\tau, v, p) - Y(t+\tau, v, p)\|^2_{nodal}},$$

where $\mathcal{T}$ are the set of 12-hour spaced times in 2020. The *nodal* norm above is area weighted, to avoid polar regions (where points are more dense) from having an undue influence:

$$\|X(t \to t+\tau, v, p) - Y(t+\tau, v, p)\|^2_{RMSE} \tag{7}$$

$$:= \frac{1}{IJ}\sum_{i=1}^{121}\sum_{j=1}^{240} w(i)|X(t \to t+\tau, v, p, i, j) - Y(t+\tau, v, p, i, j)|^2. \tag{8}$$

where (i, j) are the (latitude, longitude) indices and $w(i) \propto (\sin\phi_{i+0.5} - \sin\phi_{i-0.5})$.

For models that produce a single deterministic forecast X, MSE $\equiv \mathbb{E}\left[\|X(t+\tau) - Y(t+\tau)\|^2\right]$ (and hence RMSE) is minimized by $X \equiv \mathbb{E}[Y]$. So the best RMSE scores will be had by a forecast that predicts the (conditional) mean $\mathbb{E}[Y(t+\tau) \mid Y(t)]$. We primarily use this metric for assessing accuracy of deterministic forecasts at shorter lead times, when the distribution of $Y(t+\tau)$ is sharply peaked. As lead time τ increases, the distribution $Y(t+\tau)$ spreads out, as true dynamics is depends on information not fully contained in the initial state $Y(t)$. When this happens, $\mathbb{E}[Y(t+\tau) \mid Y(t)]$ becomes blurry and unphysical.

For models that generate ensemble forecasts we compute RMSE scores using ensemble mean $\mathbb{E}_i[X_i]$. In this setting RMSE remains informative even at later times, as the ensemble mean is taken explicitly. It's important to note that RMSE of the ensemble mean alone is not sufficient to assess the skill of the forecasting system. An ensemble of identical blurry realizations could still achieve good RMSE skill, but fail to capture extreme events and provide probabilistic insight.

F.2 Root mean squared bias (RMSB)

Biases estimate persistent differences between forecasts $X(t+\tau)$ and ground truth $Y(t+\tau)$ averaged over time. This is reported separately for each variable $v \in \mathcal{V}$ and pressure level p, lead time τ, and (latitude, longitude) coordinates (i,j).

$$\mathcal{E}_{bias}(\tau, v, p, i, j) := \frac{1}{|\mathcal{T}|} \sum_{t \in \mathcal{T}} \left[X(t \to t+\tau, v, p, i, j) - Y(t+\tau, v, p, i, j) \right],$$

RMSB is computed by taking RMSE of the bias and aggregating it over spatial dimensions.

$$\mathcal{E}_{RMSB}(\tau, v, p) := \sqrt{\frac{1}{IJ} \sum_{i=1}^{121} \sum_{j=1}^{240} w(i) \|\mathcal{E}_{bias}\|^2}$$

F.3 Continuous Ranked Probability Score (CRPS)

Ideally, evaluation metrics should be minimized when $X(t) \sim Y(t)$, rather than $\mathbb{E}Y(t)$ as is the case for MSE. One such metric is CRPS.

To understand CRPS, consider ground truth scalar random variable V, and two independent forecasts U and U'. CRPS takes the form

$$\mathbb{E}|U - V| - \frac{1}{2}\mathbb{E}|U - U'|. \tag{9}$$

The skill term $\mathbb{E}|U - V|$ penalizes forecasts that deviate from ground truth, while the spread term $(1/2)\mathbb{E}|U - U'|$ encourages well dispersed forecasts. As it turns out, CRPS is minimized just when U has the same distribution as V [15].

Following [14], we extend CRPS to multiple dimensions by summing over components. We estimate this using M ensemble members $(X^{(1)}, \ldots, X^{(M)})$ as

$$\begin{aligned}
&\mathcal{E}_{CRPS}(\tau, v, p) := \\
&\quad \frac{1}{|\mathcal{T}|} \sum_{t \in \mathcal{T}} \frac{1}{IJ} \sum_{i=1}^{121} \sum_{j=1}^{240} w(i) \Bigg[\\
&\quad \frac{1}{M} \sum_{m=1}^{M} \left| X^{(m)}(t \to t+\tau, v, p, i, j) - Y(t+\tau, v, p, i, j) \right| \\
&\quad - \frac{1}{2M(M-1)} \sum_{m,k=1}^{M} \left| X^{(m)}(t \to t+\tau, v, p, i, j) - X^{(k)}(t \to t+\tau, v, p, i, j) \right| \Bigg]
\end{aligned} \tag{10}$$

The resultant "CRPS" is minimized by any distribution X such that the marginals X_n, have the same distribution as Y_n. CRPS will therefore not require correct forecasts, but will not penalize them either.

F.4 Spread-Skill ratio

Spread-skill ratio represents the ratio of the ensemble spread (standard deviation) to skill (RMSE) of the ensemble mean. For generic scalar ground truth Y_t and ensemble of forecasts $\{X_t^{(n)}\}_{n=1}^N$, each given at times $t = 1, \dots, T$, define

$$\mu_t := \frac{1}{N} \sum_{n=1}^{N} X_t^{(n)},$$

$$\mathrm{S}^2 := \frac{1}{T} \sum_{t=1}^{T} \frac{1}{N-1} \sum_{n=1}^{N} (X_t^{(n)} - \mu_t)^2,$$

$$\epsilon^2 := \frac{1}{T} \sum_{t=1}^{T} \frac{1}{N} \sum_{n=1}^{N} (Y_t - \mu_t)^2.$$

Above, S^2 is an unbiased estimate of ensemble variance. However, the mean square error estimate ϵ^2 is biased. Taking expectations, we see it is too large by a term equal to the variance divided by N. To un-bias it, we therefore subtract S^2/N. The resultant spread skill ratio is

$$SSR = \sqrt{\frac{S^2}{\epsilon^2 - S^2/N}}.$$

This de-biasing is only used for spread-skill-ratio, and we report biased RMSE for all ensemble models.

If ensemble members X are distributed identically to the ground truth Y, then the spread-skill ratio should be equal to 1. Similar to other evaluate metrics we compute spread-skill ratio for ECMWF-ENS and NeuralGCM-ENS models for all lead times by averaging over initial time. When reporting global spread-skill ratio we perform spatial aggregation prior to computing the ratio. For spatial visualizations spread-skill ratio is computed for each latitude, longitude and level independently.

G Training

We train NeuralGCM using Adam [16], optimizing model parameters to minimize the loss function between predictions and coarsened ERA5 trajectories. Optimizer parameters are summarized in G.1. For all models we progressively extend the lead time horizon over which the loss is computed as the model trains. The schedule and the data are discussed in G.2. Before computing the loss we rescale errors between trajectories to address the differences in magnitudes of the target distributions (see G.3 for details). Finally, the two loss functions used to train deterministic and stochastic NeuralGCM are discussed in G.4 and G.6 respectively. Computational resources and training times are reported in G.7.

G.1 Optimizer settings

All NeuralGCM models were trained using Adam [16]. We used values $\beta_1 = 0.9$, $\beta_2 = 0.95$ and $\epsilon = 10^{-6}$ for all experiments based on empirical evidence from initial results at coarsest resolution. Learning rate was adjusted as a function of training iterations, linearly increasing to its maximum value over the first 2000 training steps, then remaining constant until training step 15000 when exponential decay with rate 0.5 was initiated with decay time set to 10000 steps. The only exception to these parameters was the decoder fine-tuning stage described in G.5, where warm-up is completed by step 1000 and the learning rate decays with rate 0.5 every 1000 steps starting from step 2000. Peak learning rates and total number of steps for different model configurations are reported in SI Table 4.

	NeuralGCM-2.8°	NeuralGCM-1.4°	NeuralGCM-0.7°	NeuralGCM-ENS(1.4°)
Peak learning rate	0.002	0.002	0.001	0.001
Number of training steps	38000	26000	25000	43000

Supplementary Table 4: Learning rate and number training steps used for NeuralGCM at different resolutions.

Model	Unroll lengths	Transition boundaries
NeuralGCM-2.8°	$(12, 24, 36, 48, 60, 72)$	$(2000, 5656, 10392, 16000, 22360)$
NeuralGCM-1.4°	$(12, 24, 36, 48, 60, 72)$	$(2000, 5656, 10392, 16000, 22360)$
NeuralGCM-0.7°	$(6, 12, 18, 24, 36, 48, 60)$	$(500, 2000, 4500, 8000, 12500, 18000)$
NeuralGCM-ENS (1.4°)	$(6, 12, 18, 24, 36, 48, 60, 72, 96, 120)$	$(500, 2000, 4500, 8000, 12500, 18000, 24500, 32000, 40500)$

Supplementary Table 5: Training curriculum used for NeuralGCM at different resolutions.

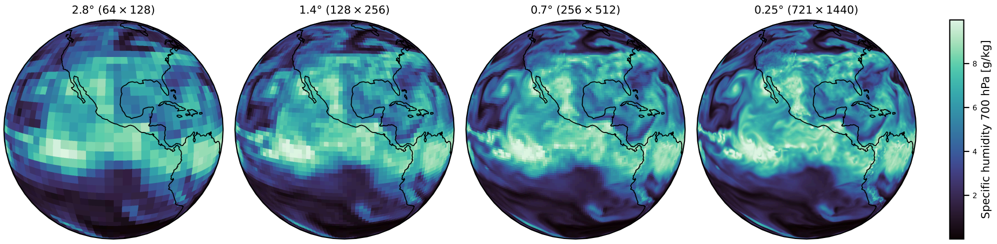

Supplementary Figure 7: Visualization of specific humidity at 700 hPa from ERA5 conservatively regridded to Gaussian grids 2.8°, 1.4°, and 0.7°, and the original ERA5 data.

G.2 Training data and unroll schedules

To train NeuralGCM models at 2.8°, 1.4° and 0.7° resolutions we regridded ERA5 data to the corresponding Gaussian grids. We use a conservative regridding scheme, which linearly aggregates contributions weighted by the relative area overlap. Final 2.8° and 1.4° models were trained on data from years 1979 through 2017 and evaluated on 2018 during the development cycle. 0.7° model and stochastic model variations were trained with data from 1979 through 2019. None of the models had any exposure to data from 2020 prior to running final model evaluations. During training we also change lead time unroll length as a function of training iterations, increasing the prediction horizon as the model improves over the course of training. The values are reported in SI Table 5.

G.3 Variable rescaling for losses

Before computing losses, trajectories of all variables are rescaled to address: (1) differences in natural scales of atmospheric variables, and (2) growth of their deviations from the ground truth as a function of lead time. The former is accomplished by dividing each atmospheric variable by standard deviation of the temporal difference between snapshots that are 24 hours apart, similar to [11]. We further adjusted scaling factors across variables to better balance out the initial skill of the model by the following additional scaling factors: geopotential 2, specific humidity 0.66, logarithm of the surface pressure (internal model representation) 5 and cloud moisture species 0.05. Our rationale for additional balancing is to prevent the loss landscape being dominated by just a few atmospheric variables. Standard deviations were estimated based on 10-60 snapshots of 24-hour differences in ERA5 data averaged over longitude, latitude, level and sample. The only trajectory that was normalized per-level was specific humidity as its values vary significantly with level. When estimating scales for variables in internal representation such as divergence, vorticity, log surface pressure, corresponding quantities were compute from ERA5 data using linear interpolation to sigma coordinates and appropriate transformations relating them to the data variables.

Each trajectory is also rescaled to account for a near linear increase in expected error variance with lead time τ. The scaling factor is $(1 + (\tau/24))^{-1/2}$ for all losses except the spectral losses, which use $(1 + (\tau/40)^4)^{-1/2}$.

Finally, in the case of filtered mean squared loss an additional time-dependent rescaling is performed which described in detail in G.4.1.

G.4 Loss for deterministic models

Our deterministic models are trained in two stages that differ in loss objectives and parameters being optimized: (1) primary training and (2) decoder fine tuning. During both stages, the loss terms take the general form of a mean squared error (MSE) on rescaled variables (Section G.3) with varying definitions of distances ρ_*

$$\mathcal{M}_*(\tau) := \frac{1}{|\mathcal{T}|} \sum_{t\in\mathcal{T}} \sum_{\sigma,v} \sum_{\substack{\tau'\in\mathcal{D} \\ \tau'\leq\tau}} \rho_*(X(t \to t+\tau', v, \sigma), Y(t+\tau', v, \sigma))^2, \tag{11}$$

where the lead time τ, in hours, is selected from

$$\mathcal{D} := \{6, 12, 18, 24, 36, 48, 60, 72, 96, 120\}.$$

During the primary stage we used a combination of three loss types to account for different types of discrepancies between NeuralGCM predictions and ERA5 data: accuracy (G.4.1), sharpness (G.4.2) and biases (G.4.2). All losses, except for the bias loss, are imposed on both "data"(i.e., pressure levels) and "model"(i.e., sigma levels) representations. This results in a training objective that is a sum of five loss terms:

$$\begin{aligned}\mathcal{L}_{Deterministic} =& \lambda_{data}\mathcal{M}_{Data} + \lambda_{spec}\mathcal{M}_{DataSpec} + \lambda_{model}\mathcal{M}_{Model} \\ &+ \lambda_{spec}\mathcal{M}_{ModelSpec} + \lambda_{bias}\mathcal{M}_{MSBias},\end{aligned}$$

where loss scales $\lambda_{data} = 20$, $\lambda_{spec} = 0.1$, $\lambda_{model} = 1$ and $\lambda_{bias} = 2$ were selected empirically. We found that even though the contributions corresponding to "spec" and "bias" terms were small (compared to the total loss), they had a positive effect on sharpness of NeuralGCM predictions. This is possibly related to the fact that the dynamical core simulates an energy cascade, maintaining the spectral energy of the simulated fields.

After the main training phase is complete, we run a short decoder fine-tuning optimization discussed in G.5 to remove spectral artifacts that arise from truncation errors in transformations from spherical harmonic to grid-point space. During this training phase we only update parameters of the decoder component of the model.

G.4.1 Accuracy loss: filtered MSE

Traditional mean squared error loss between forecast X and targets Y can be computed on pressure levels p and in spherical harmonic basis indexed by total (l), and zonal (m), wavenumbers using $\rho_{Data}(X, Y) = \|X - Y\|_{spectral}$, where

$$\|\epsilon\|^2_{spectral} := \sum_l \sum_m |\epsilon(l, m)|^2. \tag{12}$$

Up to discretization error, this is equivalent to $\|\cdot\|^2_{nodal}$, the area-weighted MSE in grid-point space representation.

When used to evaluate forecasts at longer lead time, this loss suffers from the "double penalty problem" [17] as it penalizes the model for predicting sharp features that are slightly misplaced. We address this issue by using "filtered MSE", which applies filtering to X and Y prior to computing the norm 12.

The filtering step aims to retain components of X and Y that we expect to be able to predict and drop components that cross the predictability horizon due to chaotic dynamics. We estimate such filtering parameters by analyzing the normalized MSE growth between ECMWF-HRES and ERA5 as a function of time for each variable and total wavenumber l separately SI Fig. 8(a). We chose a predictability threshold of relative error of 0.12, and determined the largest value of l^* for each lead time that should be included in the loss. Then we determined attenuation parameters for the exponential filters of order 12 that would remove total wavenumbers higher than l^* for each group, shown in SI Fig. 8(b). The effect of applying filters is shown in SI Fig. 8(c).

The *Model* terms are defined similarly to their Data counterparts, except they are computed in the encoded "model" space. This encourages the encoder/decoder round-trip to be the identity. It was found to enhance stability.

G.4.2 Sharpness loss: spectrum MSE

Spectrum MSE is defined by the distance

$$\rho_{DataSpec}(X(t \to t+\tau, v, \sigma, l), Y(t+\tau, v, \sigma, l))^2$$
$$= \sum_{l}^{\tilde{l}} \left[\mathcal{S}(X)(t \to t+\tau, v, \sigma) - \mathcal{S}(Y)(t+\tau, v, \sigma)\right]^2,$$

where $S(X)^2(..., l) = \sum_m X(..., l, m)^2$ is the spectral energy at total wavenumber l and $\tilde{l}$ corresponds to a spectral cutoff for the loss. For NeuralGCM models at 2.8°, 1.4°, 0.7° we used $\tilde{l}$ of 42, 80 and 120 respectively.

G.4.3 Bias loss: spectral bias MSE

Spectral bias MSE is slightly different from the previous two terms, as it averages over the batch and lead time dimensions prior to computing the distance norm:

$$\mathcal{M}_{MSBias}(\tau) = \sum_{\sigma,v} \left\| \frac{1}{|\mathcal{T}|} \sum_{t \in \mathcal{T}} \sum_{\substack{\tau' \in \mathcal{D} \\ \tau' \leq \tau}} \left[X(t \to t+\tau, v, \sigma) - Y(t+\tau, v, \sigma)\right] \right\|^2_{spectral}$$

This loss term discourages accumulation of bias.

G.5 Decoder fine-tuning

The final stage of training deterministic NeuralGCMs is focused on optimizing only the "Decoder" component. After the primary training phase, "Decoder" outputs contain high-frequency artifacts that are not captured by the losses imposed in spherical harmonics representation (because numerical spherical harmonics transform has non-zero kernel). To address that we freeze all of the "Encoder" and "learned physics" components of the model and optimize the "Decoder" parameters using traditional MSE computed in grid-space representation on predictions at 6, 12, 18 and 24 hours (12 and 24 hours for NeuralGCM-1.4°). We use 4000 gradient descent steps, but generally find that loss and evaluation metrics plateau after the first 1000 training steps.

G.6 Loss for stochastic models

The CRPS loss function is defined as the sum of spectral and nodal terms.

For any variable v, and initial/lead times t and τ, the *spectral* CRPS term on pressure level p is

$$\begin{aligned}\mathcal{C}_{spectral}(t,p,v,\tau) = \frac{1}{2}\sum_{l,m}\Big[&|X(t\to t+\tau,\dots,l,m) - Y(t+\tau,\dots,l,m)| \\ &+ |X'(t\to t+\tau,\dots,l,m) - Y(t+\tau,\dots,l,m)| \\ &- |X(t\to t+\tau,\dots,l,m) - X'(t\to t+\tau,\dots,l,m)|\Big],\end{aligned}$$

where X and X' denote two independent ensemble members. $\mathcal{C}_{spectral}$ is minimized just when the forecast has the correct spherical harmonic components. CRPS contributions from lower wavenumbers l encourage forecasts with correct long range correlations, which we found to be critical for training models with good performance. We excluded wavenumbers l and m from spectral CRPS loss above a maximum wavenumber of 80, because we filter out higher wavenumbers for stability in our dynamical core.

The *nodal* CRPS loss is defined similarly, but uses an area-weighted sum over latitude/longitude points, as in (10). These two terms are combined to give

$$\mathcal{L}_{CRPS}(\tau) := \sum_{t\in\mathcal{T}}\sum_{p,v}\sum_{\substack{\tau'\in\mathcal{D}\\ \tau'\le\tau}}\left[\mathcal{C}_{spectral}(t,\tau',v) + \mathcal{C}_{nodal}(t,\tau',v)\right].$$

During training, for each initial time t, and forecast time $t+\tau$, we use one observation $Y(t+\tau)$ and exactly two forecasts $X(t+\tau)$ and $X'(t+\tau)$. This is the minimum needed for an unbiased CRPS estimate (9). It is possible to construct a CRPS loss with more than two forecast samples for every observation. As it turns out, the compute budget (for each minibatch) is better spent on greater variety of initial times. That is, rather than increasing ensemble size, we select a new observation at a new initial time, $Y(s+\tau)$ for $s \neq t$, and once again create the minimum number of forecasts $X(s+\tau)$, $X'(s+\tau)$. This is due to the fact that while adding more ensemble members reduces the variance contribution of forecasts, the variance due to having one single

observation is fixed. The result of adding more forecasts would be a sub-linear variance reduction. On the other hand, increasing the number of distinct observations in a minibatch gives a linear reduction in variance.

We note that the loss functions used in NeuralGCM, which promoted a more realistic spectrum and the ability to produce ensemble forecasts, could also be applied in "pure" ML approaches.

G.7 Training resources

Training resources are described in Table 1.In all cases, we use data parallelism, running our model with one single example on each TPU device, with a batch of examples distributed across multiple TPU cores. For NeuralGCM-0.7° and NeuralGCM-ENS, we additionally use model parallelism across spatial dimensions.

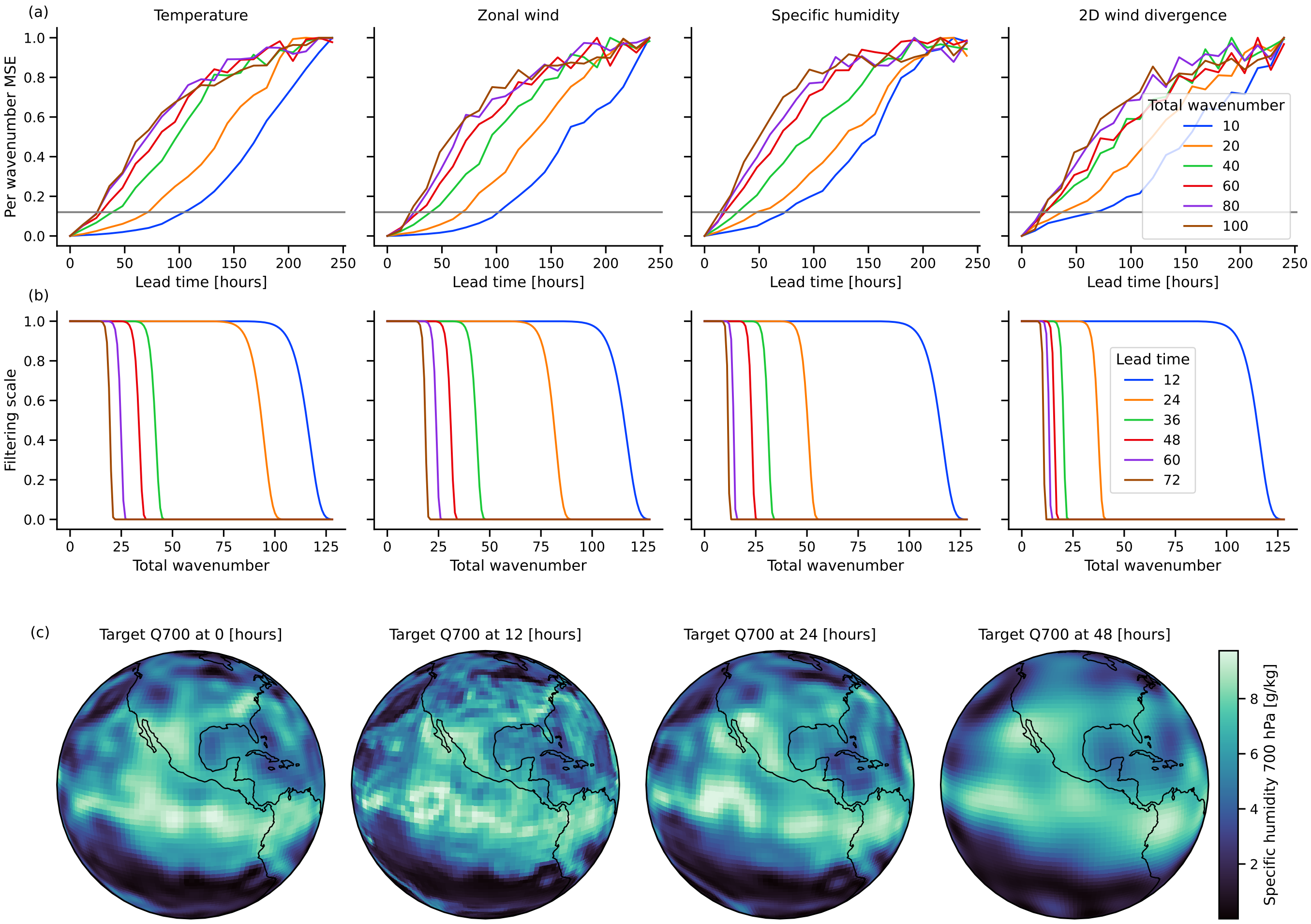

Supplementary Figure 8: Estimation of predictability based on ECMWF-HRES errors. (a) Normalized mean squared error between ECMWF-HRES and ERA5 for each total wavenumber as a function of time for temperature, zonal wind, specific humidity and horizontal wind divergence. Grey line corresponds to threshold that was used to estimate the predictability horizon. (b) Resulting filtering profiles for varying lead times and different variables. (c) Visualization of effective resolution at which errors are estimated at varying lead times. Zero hour forecasts are additionally blurred in the loss so the model does not need to match exact initial conditions.

H Additional weather evaluations

H.1 Accuracy

We assess the accuracy of deterministic NeuralGCM, along with other ML models and ECMWF-HRES, by quantifying their skill using the root mean square error (RMSE) and root-mean-square-bias (RMSB), both computed against the relevant ground truth data. As NeuralGCM was trained to predict ERA5 data, we evaluate its errors against ERA5 as the ground truth. In contrast, the ECMWF-HRES model utilizes ECMWF-HRES analysis as input, leading us to compare ECMWF-HRES against ECMWF-HRES analysis, thereby reducing the reported ECMWF-HRES errors. We additionally include scores for the NeuralGCM-ENS mean. This model is not as accurate as NeuralGCM-0.7° at short lead times, but indicates the time when the RMSE skill starts to be dominated by forecast uncertainty.

In SI Fig. 9 we plot RMSE and RMSB scorecard comparisons of NeuralGCM-0.7° and NeuralGCM-ENS against ECMWF-HRES and GraphCast across all core atmospheric variables (geopotential, temperature, specific humidity and u-component of wind) and 13 pressure levels. Similar to previous ML approaches we find that NeuralGCM-0.7° generally outperforms ECMWF-HRES across all scores (SI Fig. 9(a)), except for the top level of the atmosphere. When compared to GraphCast, we find that on short lead times NeuralGCM-0.7° performs similarly on RMSE, with some variables better modeled by GraphCast (specific humidity) and some by NeuralGCM-0.7° (geopotential) (SI Fig. 9(c)). We note that NeuralGCM-0.7°is almost 3 times coarser than GraphCast, and it is likely that higher-resolution NeuralGCM models would improve their short lead time predictions, a trend already observed in NeuralGCM models [SI Fig. 11]. At longer lead times (5-6 days) NeuralGCM-ENS achieves better RMSE scores than GraphCast across the board, underlining the utility of stochastic models at such timescales. The general trend of NeuralGCM-0.7° to outperform GraphCast at higher levels and being slightly worse at lower levels of the atmosphere is potentially caused by an additional loss weight factor introduced in GraphCast that discounts levels at lower pressure.

Contrary to RMSE, RMSB is not a priori expected to rapidly increase with lead time. NeuralGCM-0.7° model maintains consistently lower bias than ECMWF-HRES for the 10 days (SI Fig. 9(b)). GraphCast achieves lowest bias at very early lead times, but quickly degrades afterwards (SI Fig. 9(e)). NeuralGCM-ENS model has slightly higher biases compared to NeuralGCM-0.7° model, likely due to coarser spatial resolution. In SI Fig. 10 we provide similar RMSE and RMSB scorecards comparing NeuralGCM-ENS to ECMWF-ENS on 15 day forecasts.

SI Figs. 11, 13 and extended data Figs. 1, 2 compare NeuralGCM models against ECMWF-ENS across all core atmospheric variables and 3 pressure levels (500, 700 and 850 hPa), for which we had ECMWF-ENS data to compare against. Beyond two days, NeuralGCM-ENS has lower or similar error (within 1%) compared to ECMWF-ENS across all metrics (RMSE, RSMB and CRPS), with the exception of specific humidity at 850 hPa at longer lead times. At very early lead times ECMWF-ENS has better scores than NeuralGCM-ENS. However, both RMSE and CRPS values at these early times are small, indicating a negligible absolute difference between the models.

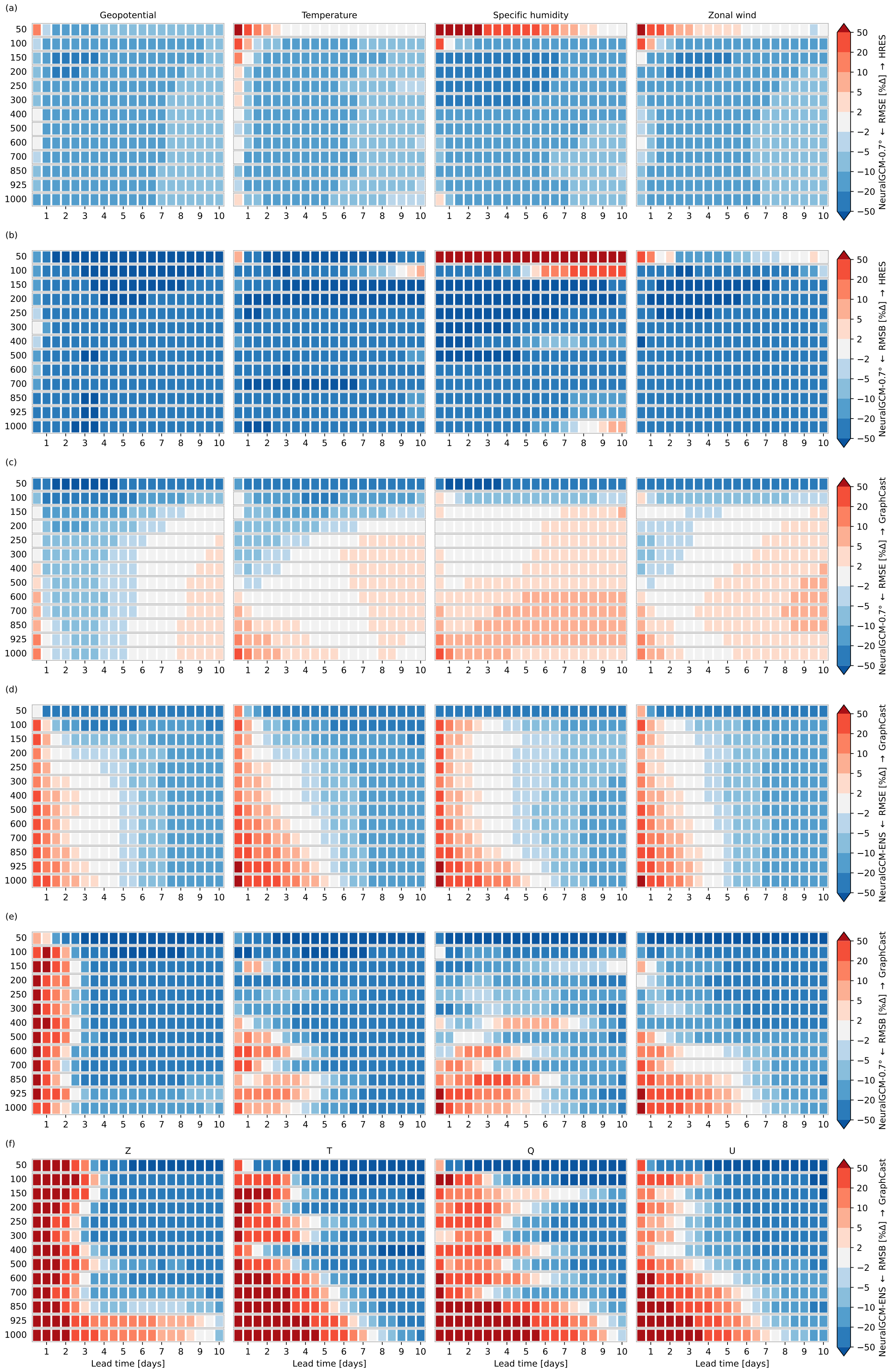

Supplementary Figure 9: Scorecards comparing NeuralGCM-0.7° and NeuralGCM-ENS models against ECMWF-HRES and GraphCast on RMSE and RSMB scores across atmospheric variables and pressure levels. (a) RMSE scorecard for NeuralGCM-0.7° vs ECMWF-HRES, (b) RSMB scorecard for NeuralGCM-0.7° vs ECMWF-HRES, (c) RMSE scorecard for NeuralGCM-0.7° vs GraphCast, (d) RMSE scorecard for NeuralGCM-ENS vs GraphCast, (e) bias RMSE scorecard for NeuralGCM-0.7° vs GraphCast, (f) bias RMSE scorecard for NeuralGCM-ENS vs GraphCast.

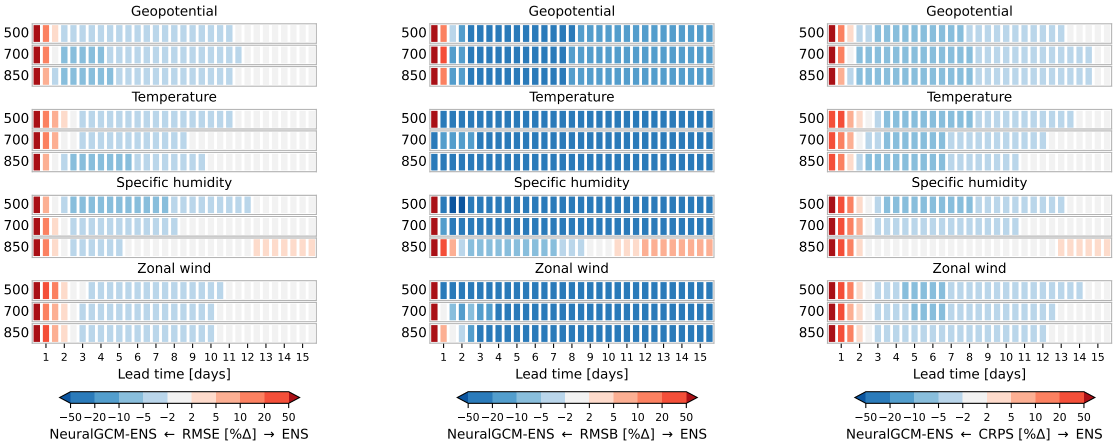

Supplementary Figure 10: Scorecards comparing NeuralGCM-ENS against ECMWF-ENS. Scorecards are shown for (a) RMSE, (b) RMSB and (c)CRPS for different atmospheric variables and pressure levels.

ECMWF-ENS's advantage possibly stems from two factors: a) NeuralGCM-ENS was optimized for 5-day rollouts, and initial condition perturbations likely improve later-stage predictions while introducing minor errors (in absolute terms) at earlier stages. Overall, these perturbations likely reduce loss. b) NeuralGCM's lower resolution (1.4°) compared to ECMWF-ENS (0.2°) limits its ability to incorporate fine-scale initial perturbations. Namely, ECMWF-ENS can introduce these perturbations while still matching IFS-analysis at a coarser scale, whereas perturbations in NeuralGCM would always increase RMSE.

SI Figs. 15, 16 and extended data Figs. 1 and 2 compare spatial metrics for 10-day forecasts from NeuralGCM-ENS and ECMWF-ENS across all core atmospheric variables on WeatherBench2 pressure levels. Overall, NeuralGCM-ENS and ECMWF-ENS have similar spatial patterns of skill relative to climatology as measured by RMSE and CRPS. Spatial biases for Neural-GCM are better overall than for ECMWF-ENS, and are also noticeably more evenly distributed. Comparing spread-skill ratio shows that unlike ECMWF-ENS, NeuralGCM-ENS is not consistently under-dispersed in the tropics.

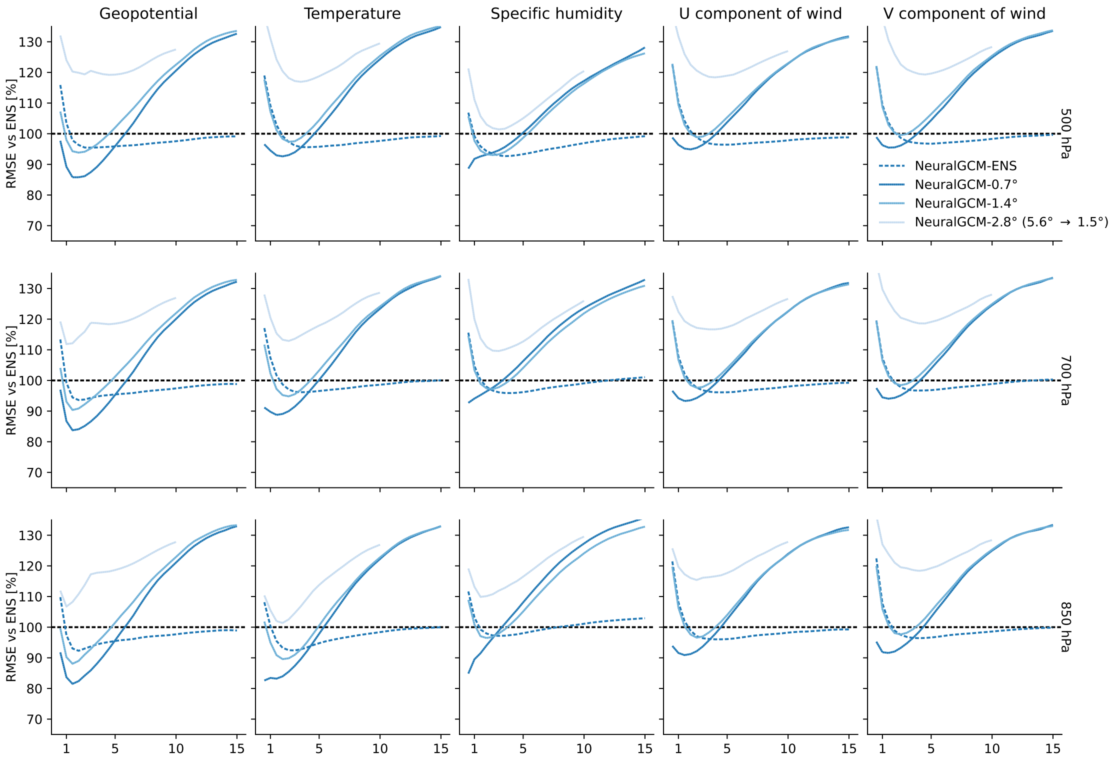

Supplementary Figure 11: Root-mean-squared-error (RMSE) relative to ECMWF-ENS for all NeuralGCM forecasts in 2020.

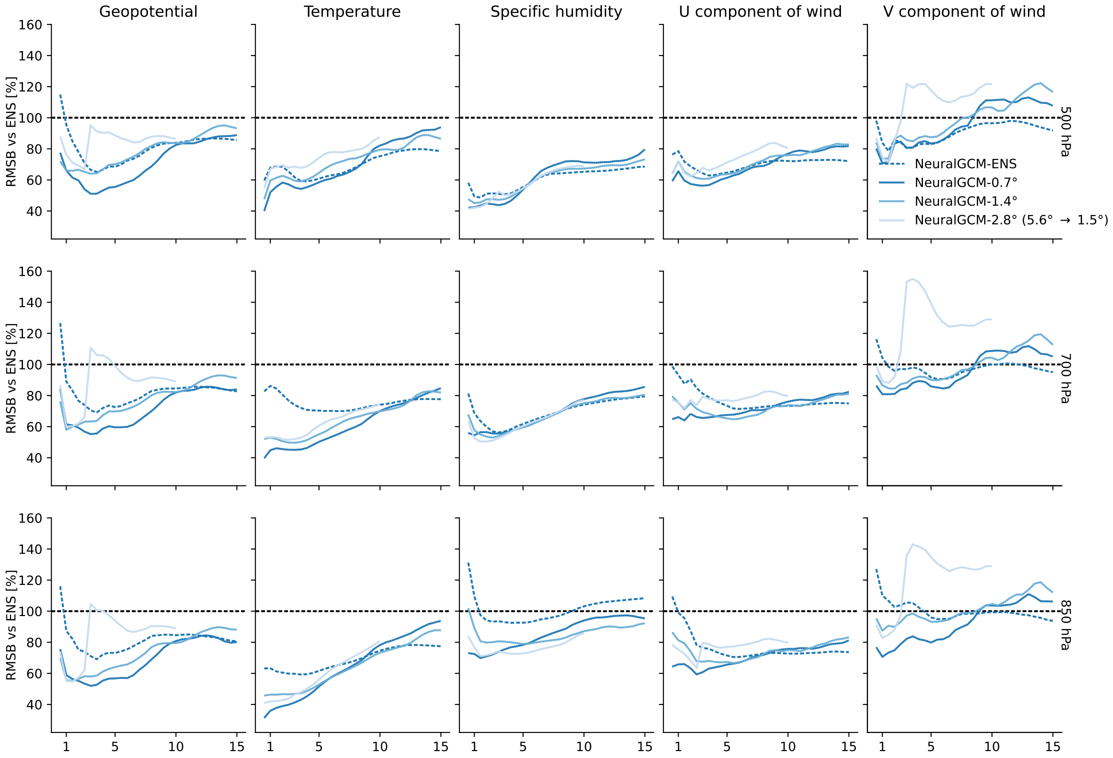

Supplementary Figure 12: Root-mean-squared-bias (RMSB) relative to ECMWF-ENS for all NeuralGCM forecasts in 2020.

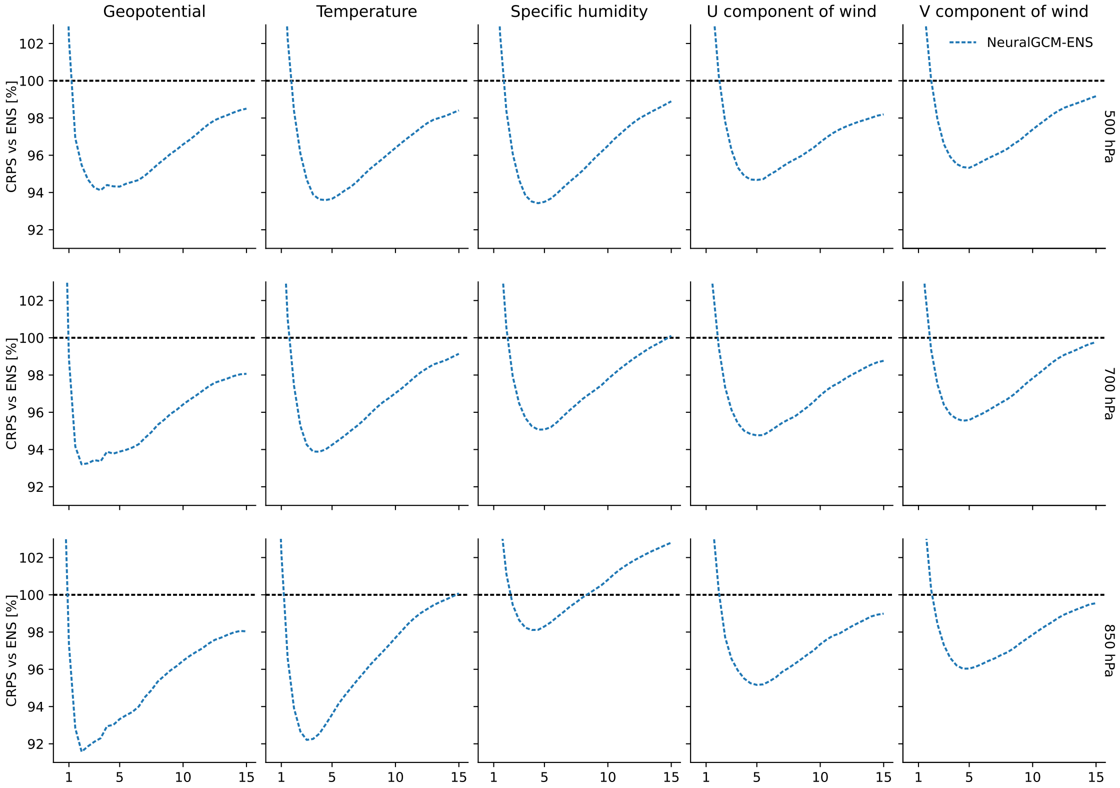

Supplementary Figure 13: Continuous ranked probability score (CRPS) relative to ECMWF-ENS for NeuralGCM-ENS forecasts in 2020.

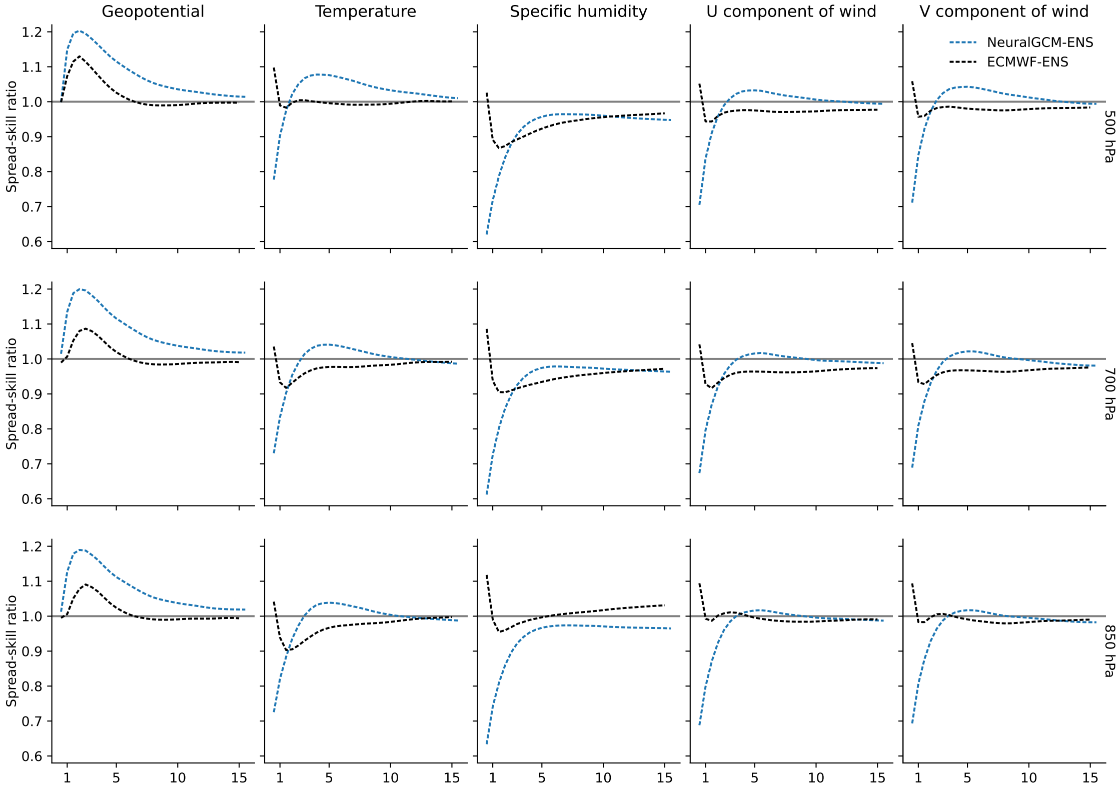

Supplementary Figure 14: Spread-skill-ratio for NeuralGCM-ENS and ECMWF-ENS forecasts in 2020.

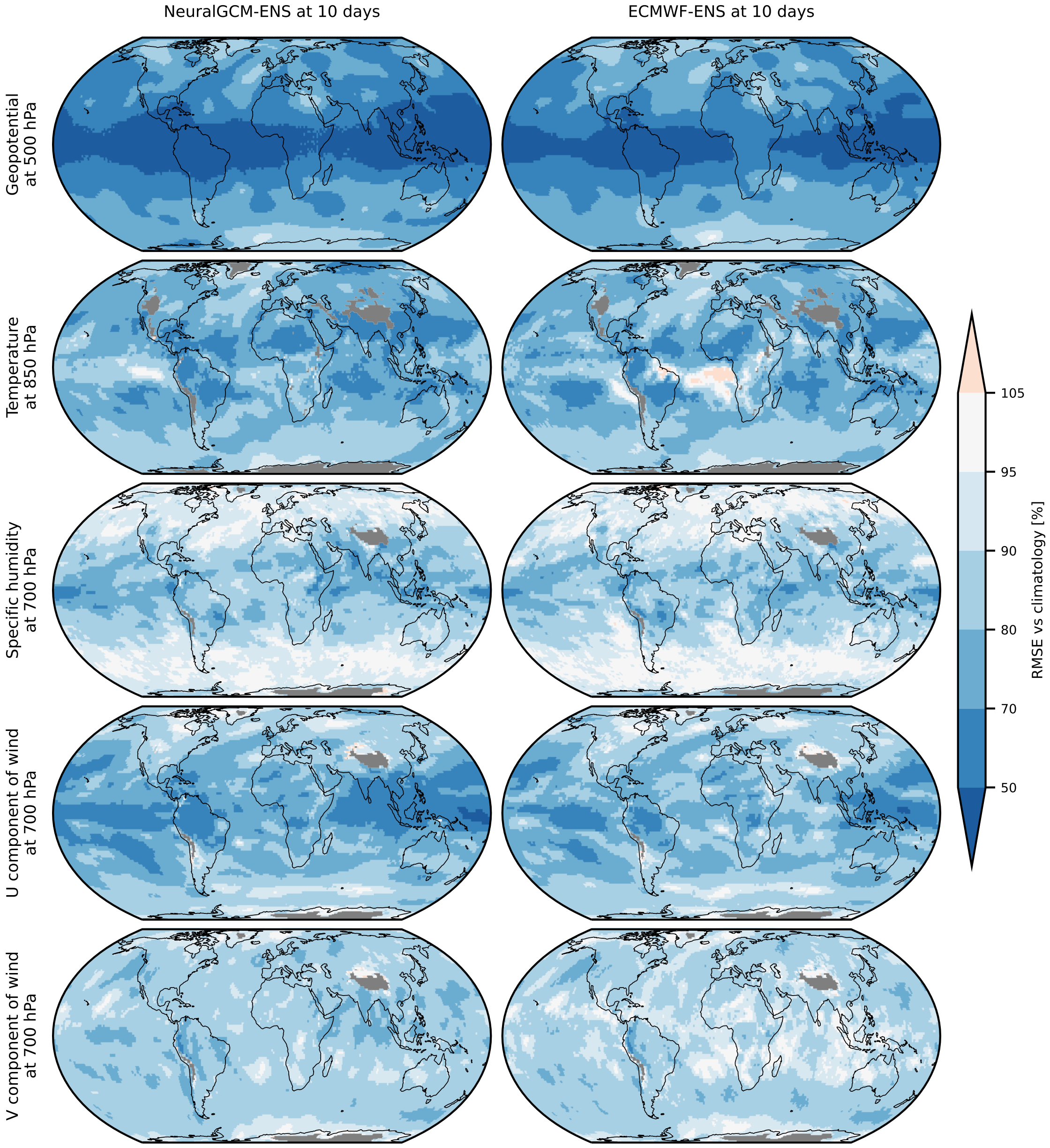

Supplementary Figure 15: Maps of root-mean-squared-error for NeuralGCM-ENS and ECMWF-ENS relative to 1990-2019 climatology for all forecasts in 2020.

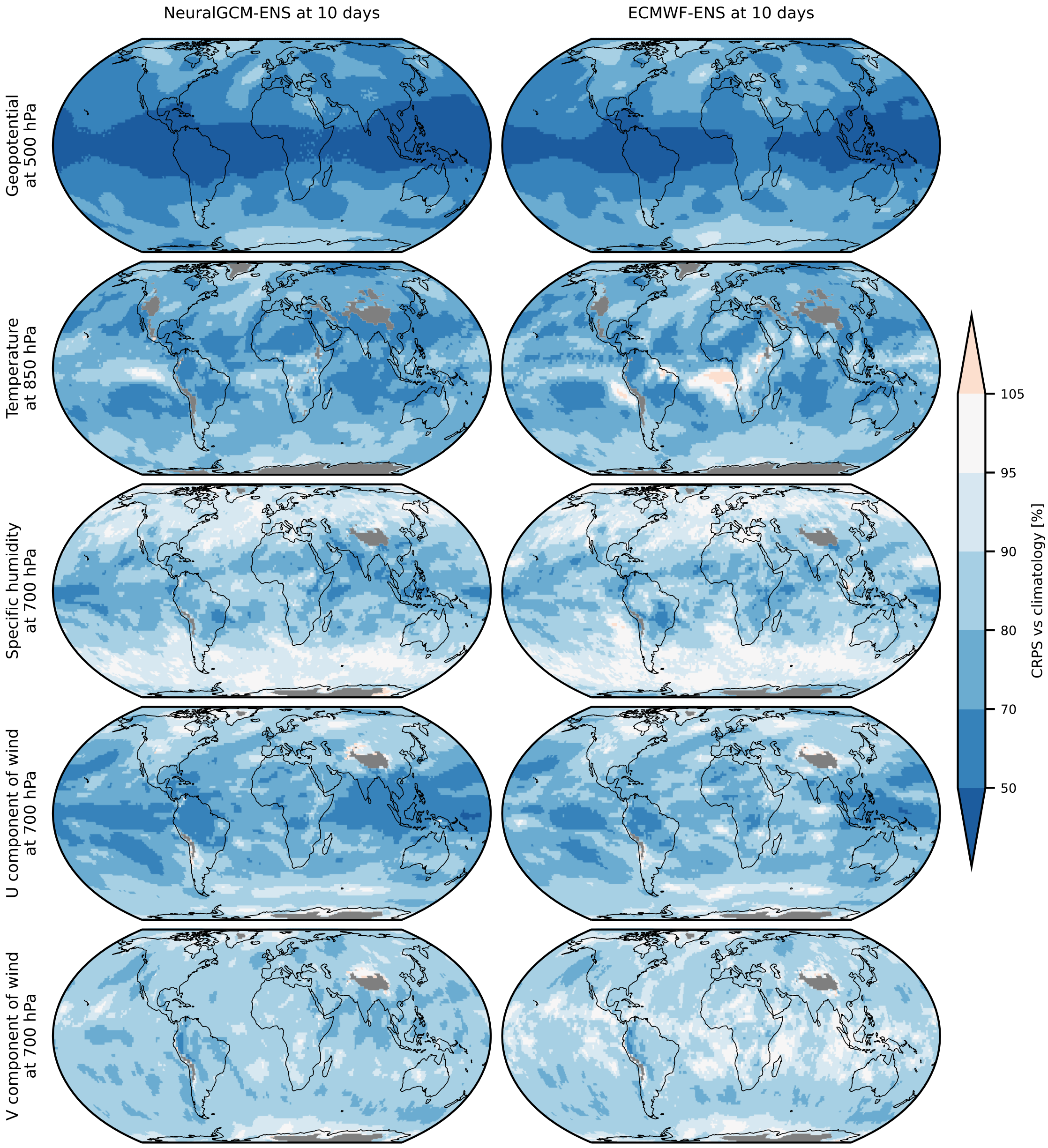

Supplementary Figure 16: Maps of average CRPS for NeuralGCM-ENS and ECMWF-ENS relative to a probabilistic climatology sampled from the years 1990-2019 for all forecasts in 2020.

H.2 Derived variables and spectra

To better understand the consistency of ML weather forecasts, we calculate a variety of the variables that can be derived from geopotential, temperature, horizontal wind velocity and specific humidity:

1. **Lapse rate** is given by

$$\frac{\partial T}{\partial z} = \left(\frac{\partial T}{\partial p}\right)\left(\frac{1}{g}\frac{\partial \Phi}{\partial p}\right)^{-1},$$

where T is temperature, p is pressure, g is the gravitional constant and Φ is geopotential.
2. **Wind speed** is given by

$$\sqrt{u^2 + v^2}$$

where u and v denote zonal and meridional wind velocity.
3. **Divergence** is given by

$$\nabla_p \cdot \mathbf{u} = \partial_x u + \partial_y v,$$

where ∂_x and ∂_y denote the partial derivatives in the zonal and meridional directions.
4. **Vorticity** is given by

$$\hat{\mathbf{k}} \cdot (\nabla_p \times \mathbf{u}) = \partial_x v - \partial_y u.$$

5. **Vertical velocity** at pressure level p_0 under the assumption of hydrostatic balance $\partial p/\partial z = -\rho g$ is given by [3]

$$-\int_0^{p_0} (\nabla_p \cdot \mathbf{u})\, dp = -\int_0^{p_0} [\partial_x u + \partial_y v]\, dp.$$

6. **Eddy kinetic energy** is given by

$$\frac{1}{2}\left[(u-\bar{u})^2 + (v-\bar{v})^2\right],$$

where $\bar{u}$ and $\bar{v}$ denote the longitudinal mean of zonal and meridional wind velocity, respectively.
7. **Geostrophic wind** is a horizontal vector given by

$$\frac{1}{2\Omega\sin\phi}\hat{\mathbf{k}} \times \nabla_p \Phi = \frac{1}{2\Omega\sin\phi}\left[-\partial_y \Phi, \partial_x \Phi\right],$$

where Ω is the rotational speed of the Earth, ϕ is latitude in radians, ∇_p is the gradient on constant pressure levels and Φ is geopotential [18].

8. **Ageostrophic wind** is the horizontal wind vector minus geostrophic wind.
9. **Total column moisture** quantities are given by a vertical integral over pressure levels,

$$\frac{1}{g}\int q\,dp,$$

where g is the gravitational constant and q is the desired moisture species (humidity/vapor, cloud liquid or cloud ice).
10. **Integrated vapor transport** is given by

$$\frac{1}{g}\sqrt{\left(\int_{p_0}^{p_1} qu\,dp\right)^2+\left(\int_{p_0}^{p_1} qv\,dp\right)^2},$$

where q is specific humidity, u is zonal wind, v is meridional wind, $p_0 = 300\,\mathrm{hPa}$ is the minimum pressure and $p_1 = 1000\,\mathrm{hPa}$ is the maximum pressure [1].
11. **Relative humidity** is given by

$$\frac{q/(1-q)}{\epsilon e_s/(p-e_s)}$$

where $\epsilon = 0.622$ and e_s the saturation vapor pressure in hPa is

$$e_s = 6.112\exp[17.67(T-273.15)/(T-29.65)],$$

where T is the temperature in Kelvin, which is the formula implemented by MetPy [19].

All derivatives are calculated using second-order finite differences, and vertical integrals are calculated using trapezoidal integration. Code for calculating these variables has been added to the WeatherBench2 codebase [14].

SI Figs. 17 and 18 plot a variety of example 7-day forecast fields and power spectra averaged over many weather forecasts, based archived weather forecasts from WeatherBench2. To consistently compare predictions from models with different resolutions, all calculations are performed after conservative regridding to a 1.5° equiangular grid on 37 pressure levels. Variables are excluded if calculating them requires fields not archived as part of WeatherBench2 for a particular forecast model, including vertical integrals or derivatives if not all pressure levels are available. We omit NeuralGCM-ENS because conservative regridding from a 1.4° Gaussian to a 1.5° equiangular grid introduces aliasing artifacts that are particularly evident for derived variables.

H.3 Visualization of ensemble weather forecasts

To verify that NeuralGCM-ENS produces reasonable looking forecasts of all variables, we plot maps of forecast fields in SI Fig. 19 and vertical profiles in SI Fig. 20 and SI Fig. 21. Here we compare to ERA5 after conservative horizontal regridding to the native resolution of NeuralGCM-ENS. The forecasts look qualitatively similar to

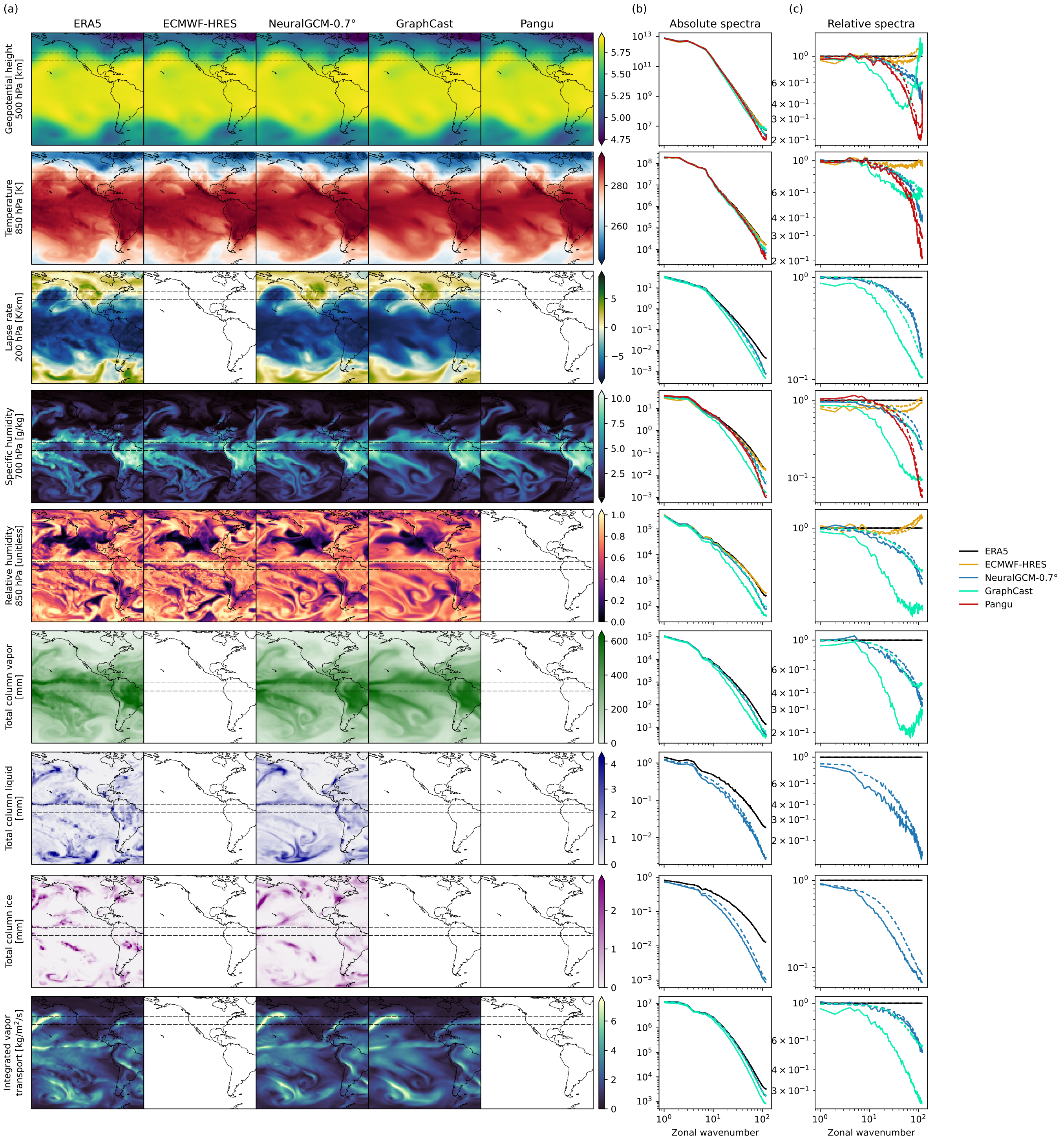

Supplementary Figure 17: Forecasts and power spectra for forecasts of thermodynamic variables. (a) Example 7-day forecasts initialized at 2020-01-01T00. (b) Absolute spectral density for 1-day (dashed) and 7-day (solid) forecasts of the indicated field, averaged over all forecasts initialized in 2020 and either over the tropics ($[-15°, 15°]$ North) or the extratropics ($[25°, 55°]$ North) as indicated by the area between dashed lines in panel (a). (c) Normalized spectral density, given by the absolute spectral density divided by the spectral density of ERA5.

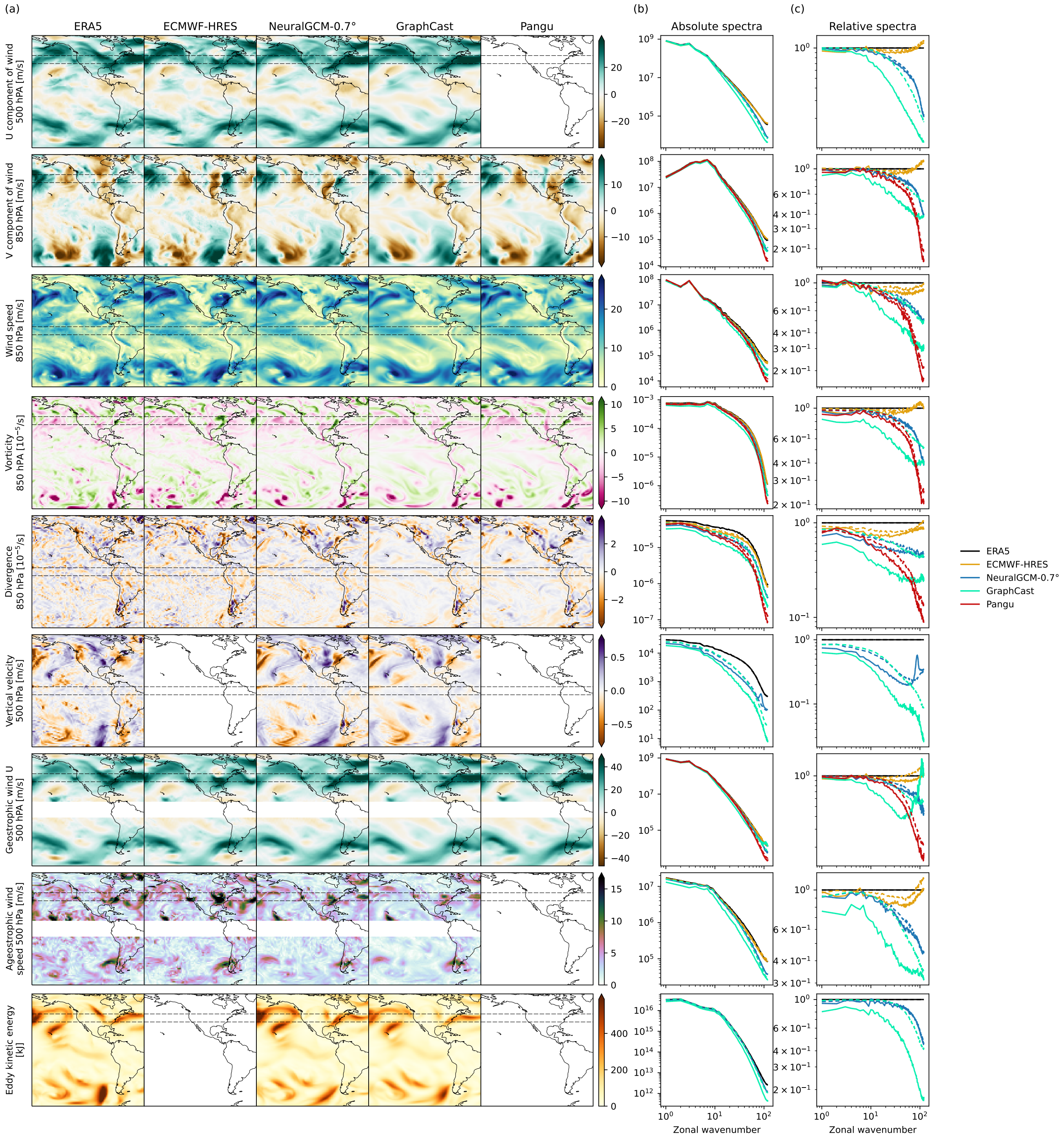

Supplementary Figure 18: Forecasts and power spectra for forecasts of wind variables, like in SI Fig. 17. Note that vertical velocity is recalculated for GraphCast from horizontal wind, rather than using the vertical velocity prediction directly output by GraphCast.

ERA5, except the cloud variables (specific cloud liquid water content and specific cloud ice water content) occasionally take on small in magnitude negative values, which is not physically possible. Fixing this issue represents an improvement opportunity for improving future versions of NeuralGCM.

H.4 Evaluation of lower resolution models

In SI Figs. 11 and 12 we compare RMSE skill and root mean squared bias of NeuralGCM models at 0.7°, 1.4° and 2.8° resolutions, evaluated on the year 2020. Similar to the main text we normalize the results against ECMWF-ENS for easier comparison. Across all atmospheric variables we find that increasing the level of detail improves RMSE. Note that the skill of NeuralGCM-2.8° is evaluated on 5.6° and then rescaled by the ratio of ECMWF-HRES errors at 1.5° and 5.6° resolutions to estimate model performance without upsampling coarse predictions.

SI Fig. 22 compares power spectra of core atmospheric variables for NeuralGCM models of different resolution at 1 and 7 days into the forecast.

H.5 Diagnosing precipitation minus evaporation

To diagnose precipitation minus evaporation rate $(P-E)$ we calculate:

$$P - E = \frac{1}{g}\int_0^1 \sum_{\mathrm{i}} \left(\frac{dq}{dt}\right)_{\mathrm{i}}^{\mathrm{NN}} p_{\mathrm{s}} d\sigma \tag{13}$$

where p_{s} is the surface pressure, and $\sum_{\mathrm{i}}(\frac{dq}{dt})_{\mathrm{i}}^{\mathrm{NN}}$ is the sum of the water species tendencies predicted by the neural network. To compare against reanalysis data we use the precipitation rate and evaporation rate from ERA5.

Extended data Fig.4a,b shows the daily distribution of precipitation minus evaporation rates from NeuralGCM-0.7° forecasts alongside ERA5. The distribution of precipitation minus evaporation rates has been normalized such that the area under the points is equal to one. We observe that the precipitation rate distribution from NeuralGCM-0.7° aligns closely with the ERA5 distribution in the extratropics. However, NeuralGCM-0.7° tends to underestimate extreme events in the tropics. For weather forecasting, we note that the average forecast (averaged across all initial conditions for the third day of prediction) aligns well with the spatial distribution seen in ERA5 (extended data Fig.4c,d). To underscore the differences between ERA5's $P-E$ and NeuralGCM-0.7°'s diagnosed $P-E$, we present snapshots of daily $P-E$. While there are overarching similarities between these snapshots, it's evident that in the tropics, NeuralGCM-0.7° tends to moderate extreme events (extended data Fig.4e,f).

We note that the calculation of $P-E$ assumes that the dynamical core is responsible for all horizontal motions; thus, $P-E$ could be diagnosed using Eq. 13. However, the physics module might also learn to correct errors originating from inaccuracies in the dynamical core (e.g., as a result of calculating advective tendencies on a coarse grid), which may introduce errors into the calculation of $P-E$. Given that $P-E$ calculated from NeuralGCM-0.7° appears generally consistent with ERA5 in the weather forecasting scenario (extended data Fig.4), this suggests that any error is likely small.

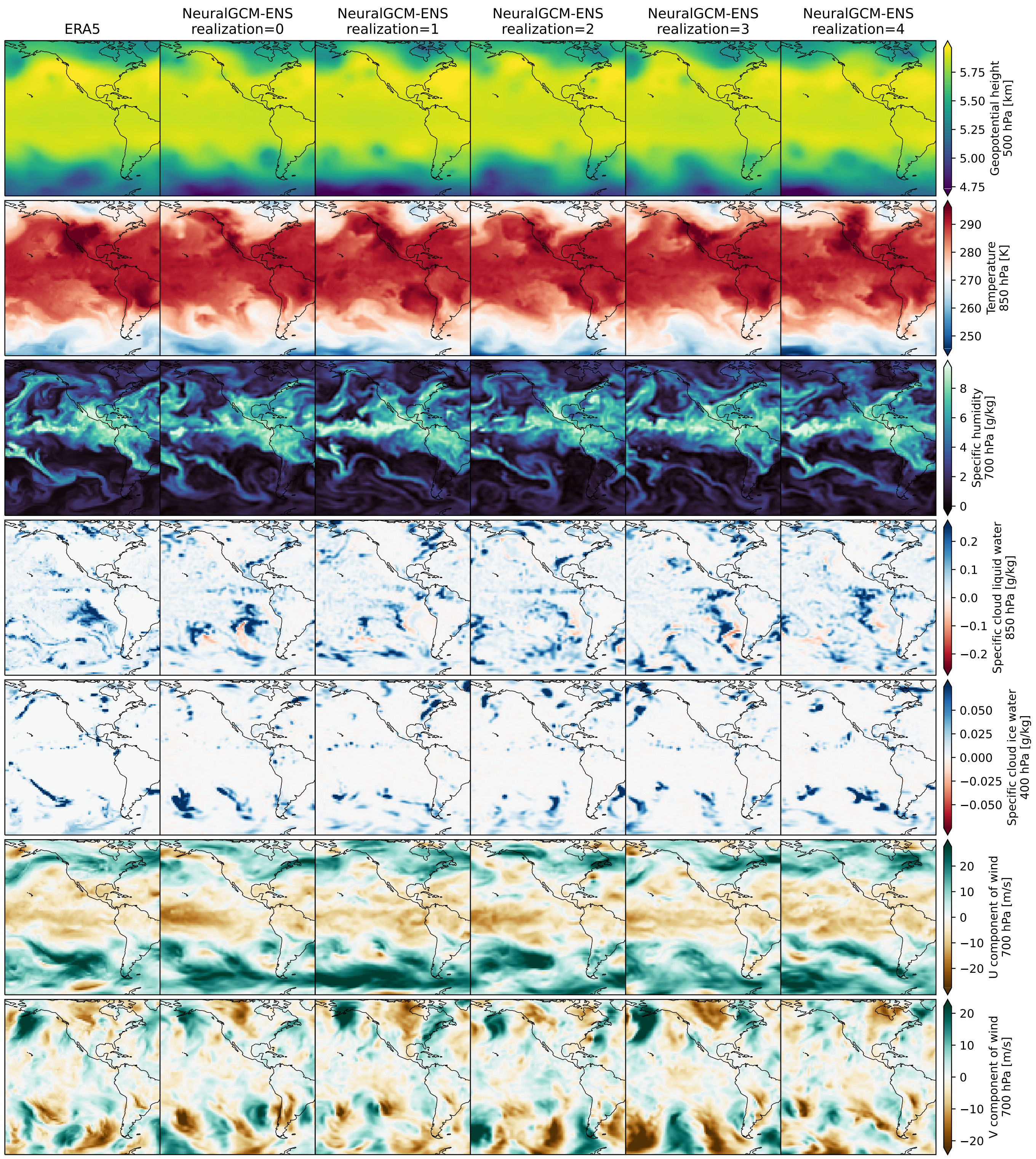

Supplementary Figure 19: Maps of five ensemble realizations of +15 day forecast fields from NeuralGCM-ENS initialized at 2020-08-22T12z, compared to ERA5.

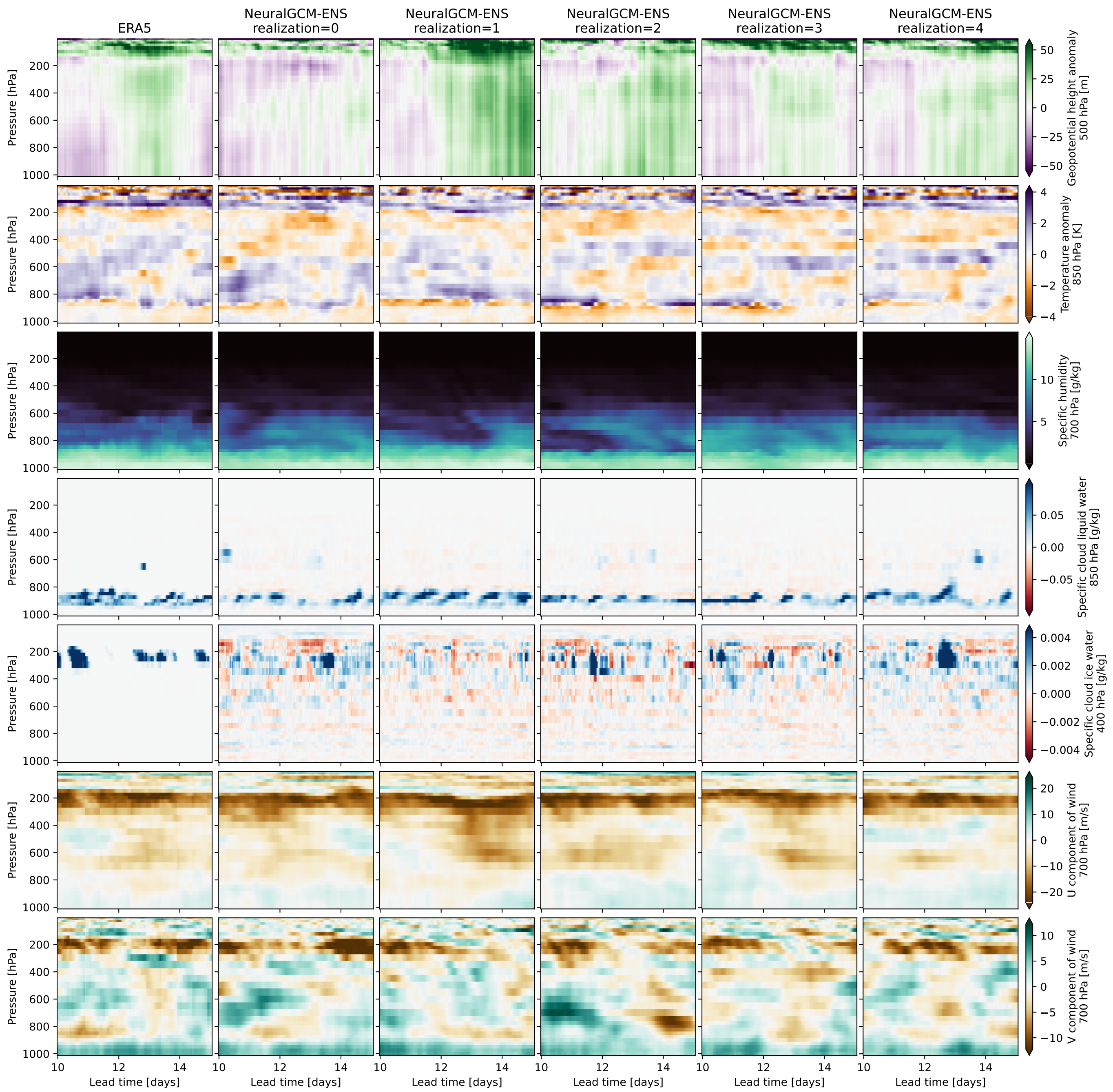

Supplementary Figure 20: Vertical profiles of five ensemble realizations of 10-15 day forecast fields at 0° N 0° W over the tropical Pacific ocean, from NeuralGCM-ENS initialized at 2020-08-22T12z, compared to ERA5.

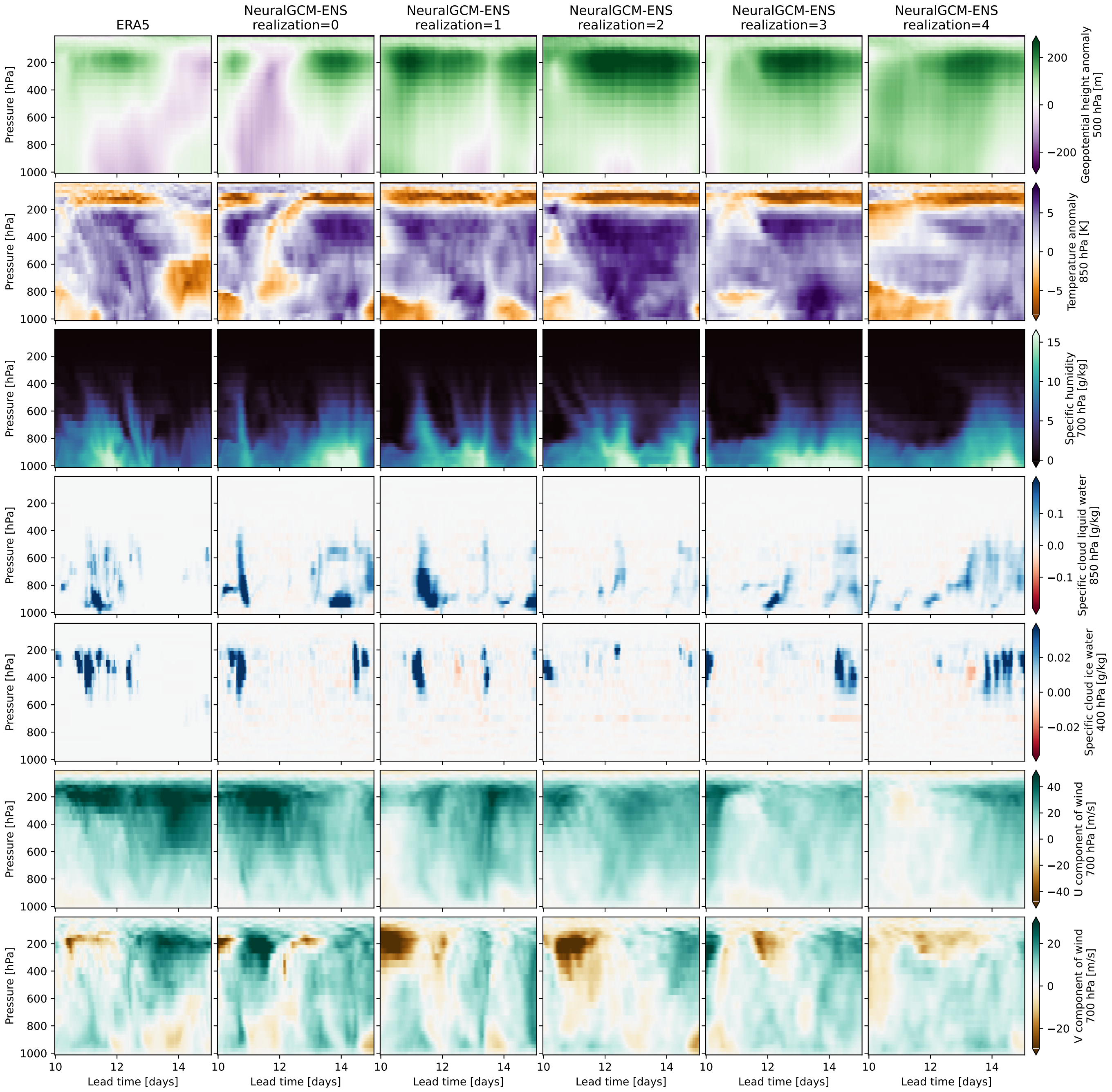

Supplementary Figure 21: Like SI Fig. 20, but at 42.3° N 71.1° W over Boston, MA, USA.

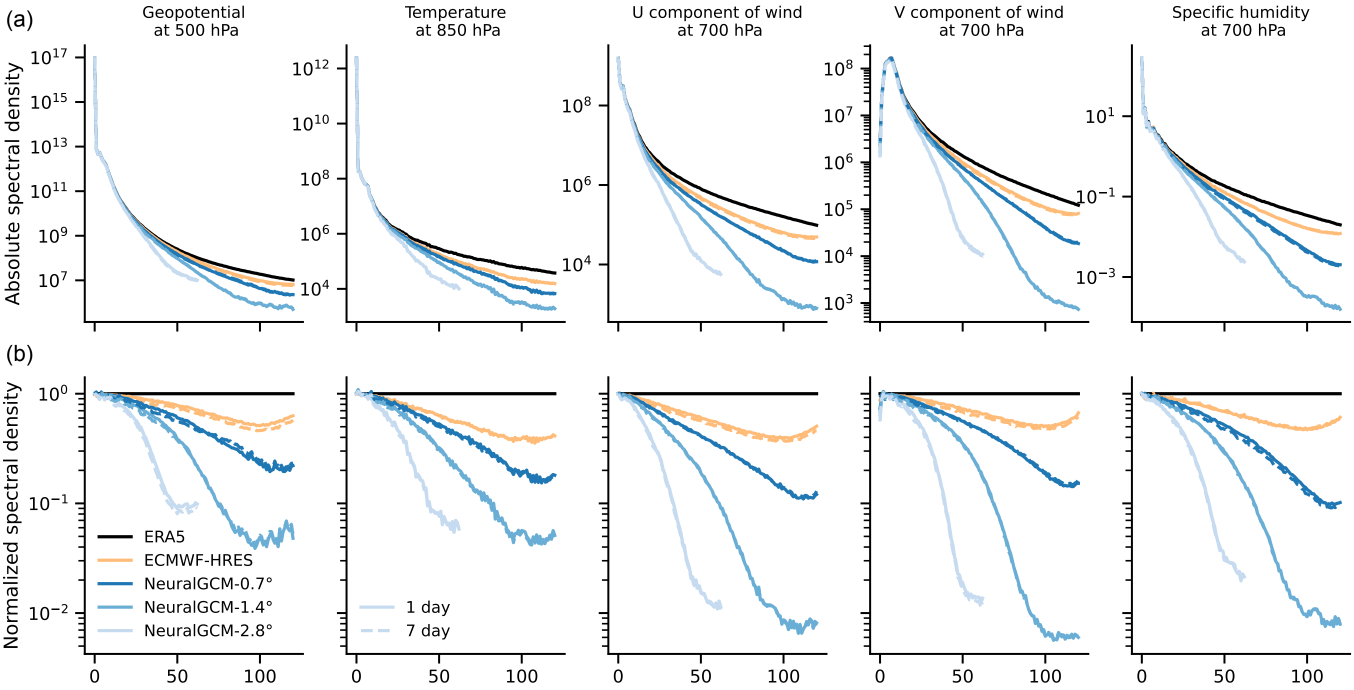

Supplementary Figure 22: Comparison of NeuralGCM models at different resolutions on zonal power spectra averaged over latitudes 15° to 55° for core atmospheric variables. (a) Absolute spectral density. (b) Spectral density normalized against ERA5.

However, at lower resolutions, the error could be larger. For example, extended data Fig 5 displays the climatology of $P-E$ calculated with NeuralGCM-1.4°, which shows clear artifacts, although it remains unclear whether these artifacts stem from the aforementioned error in calculation or another issue.

In this work, we only diagnosed precipitation minus evaporation from NeuralGCM. However, in future work, we plan to develop a scheme to reformulate NeuralGCM to predict precipitation and evaporation separately. This could be achieved, for example, by using conventional parameterization to estimate evaporation (and then calculating precipitation by adding it to P-E). Another approach could involve training a neural network (NN) specifically to predict evaporation, potentially optimized to predict the same fluxes as those in ERA5 data.

H.6 Ablation tests

H.6.1 Different loss functions

To train deterministic NeuralGCM models we used a combination of 3 different loss functions (sections G.4.1, G.4.2,G.4.3). To investigate the effect of utilizing these loss terms, we trained NeuralGCM-2.8° models while omitting certain loss terms and compared the skill and spectrum of models employing various loss terms. Specifically, we

trained models: (a) without the bias loss (section G.4.3), (b) without applying filtering to the MSE loss (section G.4.1), and (c) excluding spectral loss, bias loss, and filtering on the MSE, resulting in a simple MSE loss.

We find that removing these loss terms, or removing the MSE loss filter, enhances the RMSE score (SI Figs. 23a-c). For example, adopting solely the MSE loss - a common approach in ML algorithms for weather forecasting - enabled us to enhance our skill by several percent. Namely, the RMSE of NeuralGCM deterministic models could be improved by using an MSE loss instead of the losses we used in the paper. However, removing these loss terms leads to a degradation of the simulation spectrum over time (SI Figs. 23d and 24). Such a degradation of the spectrum over time would likely harm the ability to conduct climate simulations, and would make the NeuralGCM forecasts less useful as they would not resemble realistic atmospheric states. The spectral degradation observed in the model employing MSE loss suggests that the primary cause of such degradation in previous ML-based models [1, 20] could potentially be addressed by introducing new loss terms. However, these modifications might adversely affect the models' accuracy.

H.6.2 Training on shorter rollouts

To investigate the benefits of training on longer rollouts (i.e., optimizing for extended prediction periods), we trained a NeuralGCM-2.8° model exclusively on predictions up to 12 hours, while keeping all other model parameters identical to the default NeuralGCM-2.8° model. We find that the model trained on shorter rollouts not only exhibit deteriorated performance in terms of RMSE but, more critically, tend to be significantly less stable (SI Fig. 23a-c). For example, the sharp increase in RMSE after 13 days is associated with model instabilities (SI Fig. 23a-c).

Additionally, we attempted to train a model on 1-hour rollouts (which is a single physics timestep for NeuralGCM-2.8°) but found that the trained model becomes unstable after very few days (not shown).

H.6.3 Learning curve - pure ML vs hybrid

To estimate the volume of training data which is beneficial for NeuralGCM we trained several NeuralGCM-2.8° models using varying amounts of data (all models were trained on the most recent years up to 2017, but each utilized a different number of years for training). We find that for weather forecasting NeuralGCM-2.8° does not benefit from incorporating more than the last 21 years of data (SI Fig. 25). This finding might be related to variations in the ERA5 dataset, in which recent years uses assimilated data sources which are more similar to the year on which we tested these models (2018). In contrast older years likely incorporate fewer or different assimilated data sources relative to the year on which we tested the model.

Next, we wanted to assess whether the NeuralGCM requires a different quantity of training data compared to a purely ML-based approach. To this end, we trained multiple NeuralGCM-ML-only-2.8° models, which are similar to NeuralGCM-2.8° but lack a dynamical core, making them solely ML-based. We find that incorporating the dynamical core enhances performance compared to ML-only model (SI Fig. 25), as anticipated, given that the single-column parameterization structure we employ limits

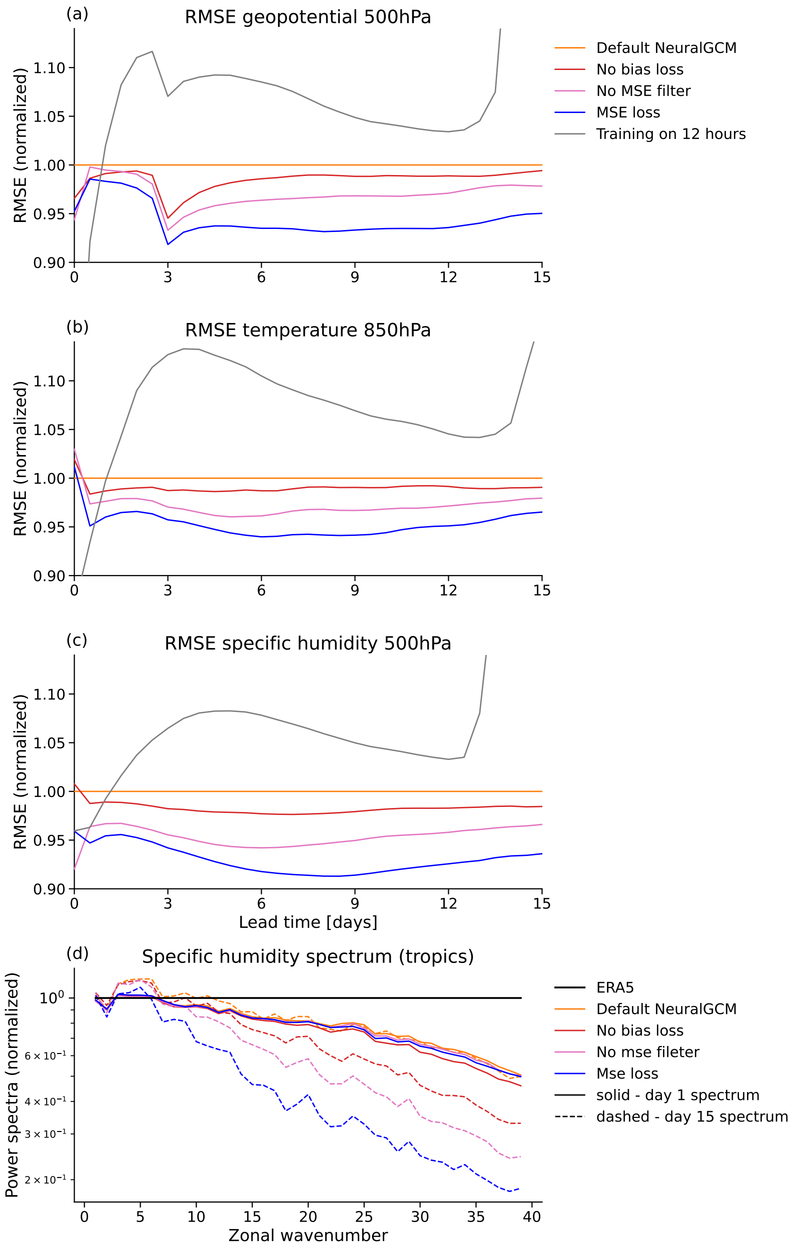

Supplementary Figure 23: Accuracy and spectrum analysis for models using different loss terms and models trained on different rollout lengths. (a-c) RMSE normalized by the RMSE of the default NeuralGCM-2.8° model, as a function of prediction lead time for simulations utilizing various loss functions: the default NeuralGCM-2.8° loss function (sum of filtered-MSE, spectral and bias losses; orange), a model trained without the bias loss term (red), a model trained without the filter on the MSE loss (pink), and a model trained with simple MSE loss function (without the spectral loss, without bias loss, and without the filter on the MSE; blue) and a model trained only up to 12 hour rollouts (grey), for (a) geopotential at 500 hPa, (b) temperature at 850 hPa, and (c) specific humidity at 700 hPa. Panel (d) depicts the zonal spectrum of specific humidity at 850 hPa in the tropics (defined from 15S-15N), normalized by ERA5 spectrum for the aforementioned models (except for the model model that is trained up to 12 hours as the spectrum diverges at day 15) and also includes the ERA5 spectrum (black). Solid lines show 1-day predictions and dashed lines show 15-day predictions. The zonal spectrum is truncated at wavenumber 40 to facilitate a clearer comparison between the models.

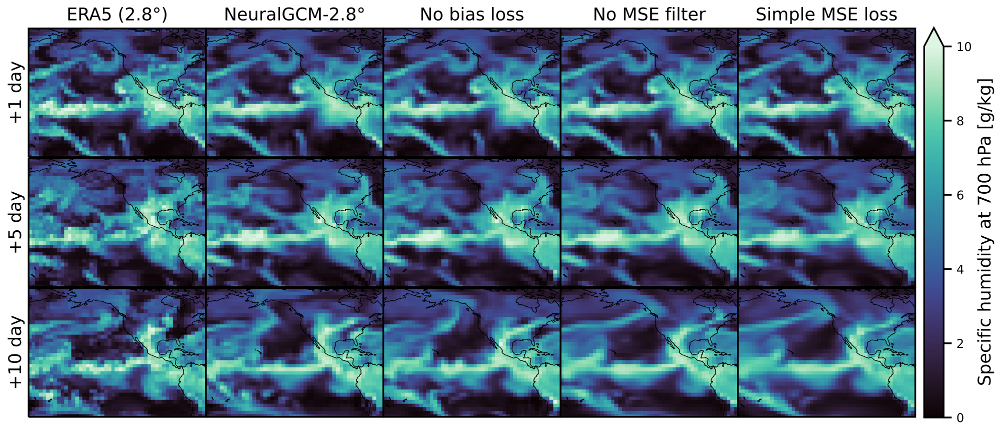

Supplementary Figure 24: Case study on the impact of different loss functions on forecast snapshots. The plot illustrates the specific humidity at 700 hPa for 1-day, 5-day, and 10-day forecasts from each model across latitudes 30S-70N and longitudes 162-300 for ERA5, NeuralGCM-2.8° as well as NeuralGCM-2.8° variants trained without bias loss, without an MSE filter, and with a simple MSE loss (excluding spectral loss, bias loss, and MSE filter). All models are at 2.8° resolution (with ERA5 data regridded to match this resolution). All forecasts are initialized at 2021-08- 25T00z.

the ability to accurately learn horizontal advective tendencies. Interestingly, we find that the inclusion of the dynamical core does not reduce the amount of necessary training data (both NeuralGCM-2.8° and NeuralGCM-ML-only-2.8° have a similar learning curve - SI Fig. 25). We hypothesize that the single-column approach might inherently reduce the training data needed, suggesting that such a structure could potentially require less data than state-of-the-art ML models for weather forecasting. However, this idea remains speculative, as direct tests of this hypothesis have not yet been conducted.

H.7 Instability case study

SI Figs. 26 and 27 show examples of an instabilities that were found in NeuralGCM-1.4° and NeuralGCM-0.7° simulations. The figures illustrates the progression of two instability case-studies over time.

SI Fig. 26 presents an example of instability observed in a NeuralGCM-1.4° simulation. Specifically, it illustrates that on day 388, the temperature field in the simulation appears realistic. However, by day 389, the 12-hour temperature tendencies at 1000 hPa (SI Fig. 26j) exhibit an unrealistic strong local tendencies at the South of the Indian Ocean. This unrealistic signal continues to intensify over time, and by day 392, the instability has significantly intensified and propagated throughout the Indian ocean (SI Fig. 26d).

SI Fig. 27 presents an example of instability observed in a NeuralGCM-0.7° simulation. Specifically, it illustrates that on day 138, the temperature field in the simulation appears realistic. However, by day 139, the 12-hour temperature tendencies in the

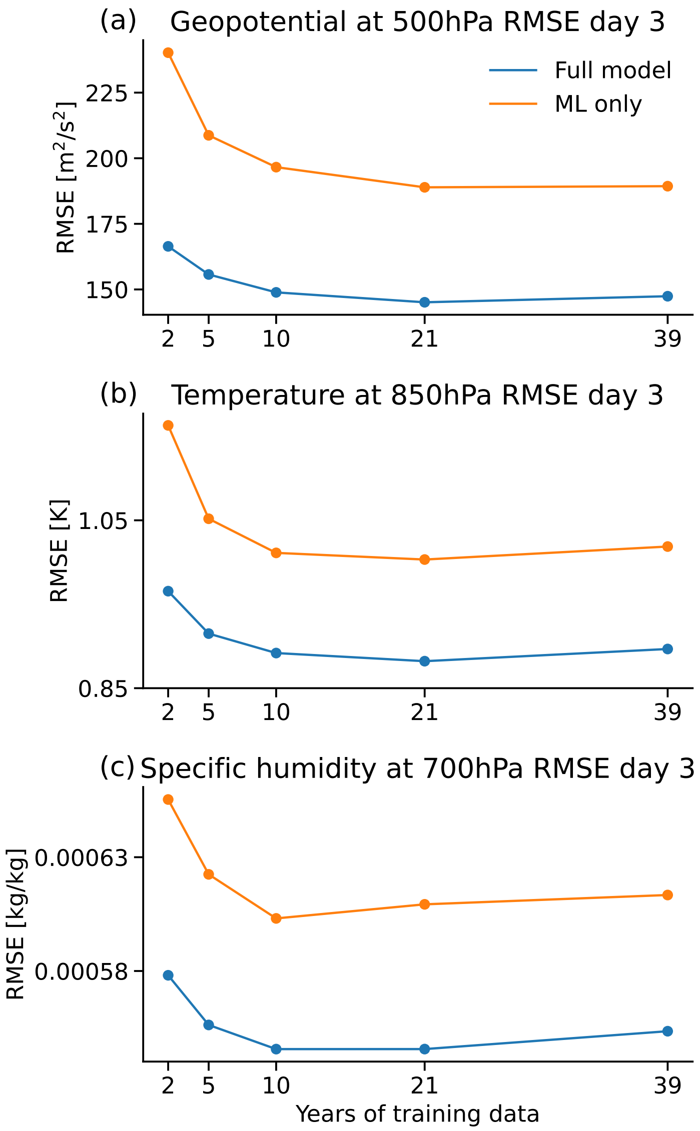

Supplementary Figure 25: Learning curves comparing the hybrid NeuralGCM model with the ML-only variant of NeuralGCM. RMSE as a function of the number of training years (with all models trained on the years leading up to 2017 and evaluated on data from 2018, utilizing varying lengths of training data) for both NeuralGCM-2.8° (blue) and its ML-only counterpart, NeuralGCM-ML-only-2.8° (orange), which is similar to NeuralGCM-2.8° but without the dynamical core. The RMSE values are presented for forecasts at day 3 for (a) geopotential at 500hPa, (b) temperature at 850hPa, and (c) specific humidity at 700 hPa.

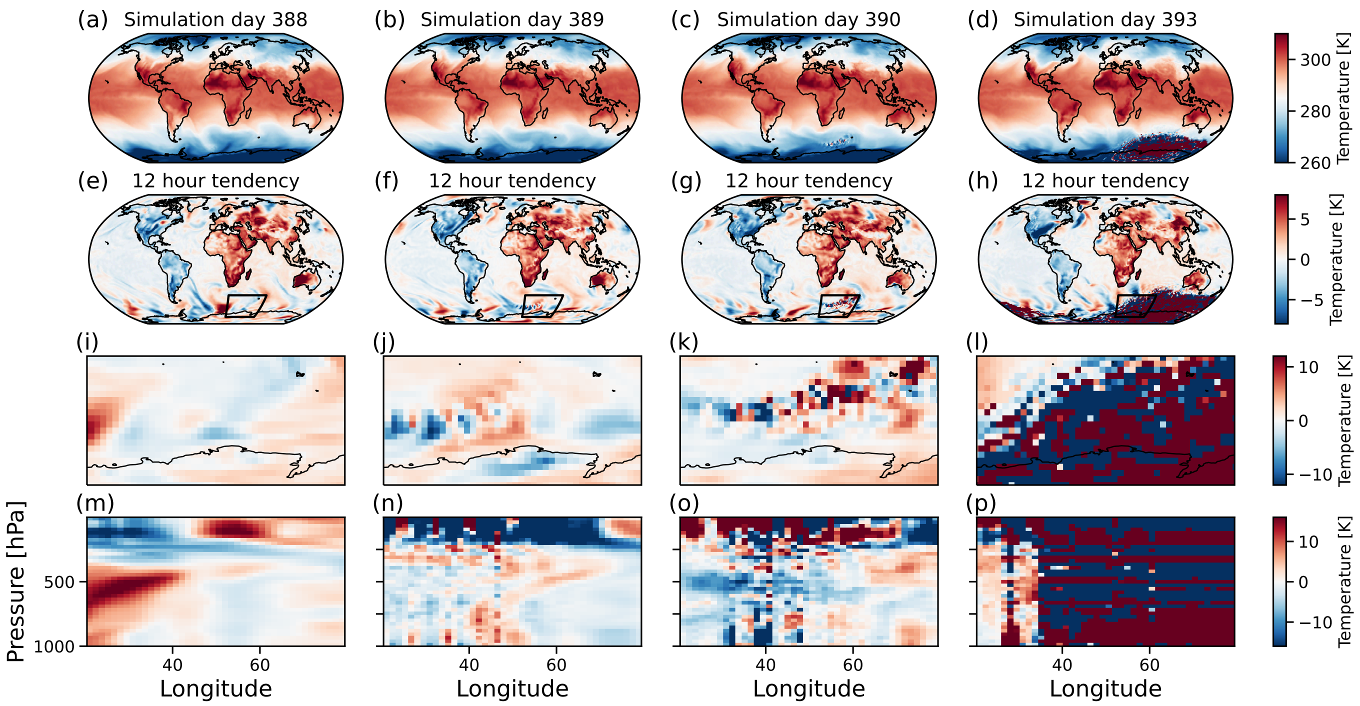

Supplementary Figure 26: Case study on instability in the NeuralGCM-1.4° model. (a-d) The temperature, (e-h) 12 hour temperature change at 1000hPa (i-l) zoom into longitudes 20 to 80 and latitudes 75S to 45S and (m-p) the vertical structure of 12 hour temperature change at latitude 60S, for different simulation lead times: day 388 (left column), day 389 (second column), day 390 (third column) and day 392 (right column). The simulation was initialized at 09-18-2019.

stratosphere (SI Fig. 27n) and at 1000 hPa (SI Fig. 27j) exhibit an unrealistic strong high wavenumber component. This unrealistic signal continues to intensify over time, and by day 155, the instability has significantly intensified and propagated throughout the tropics (SI Fig. 27d).

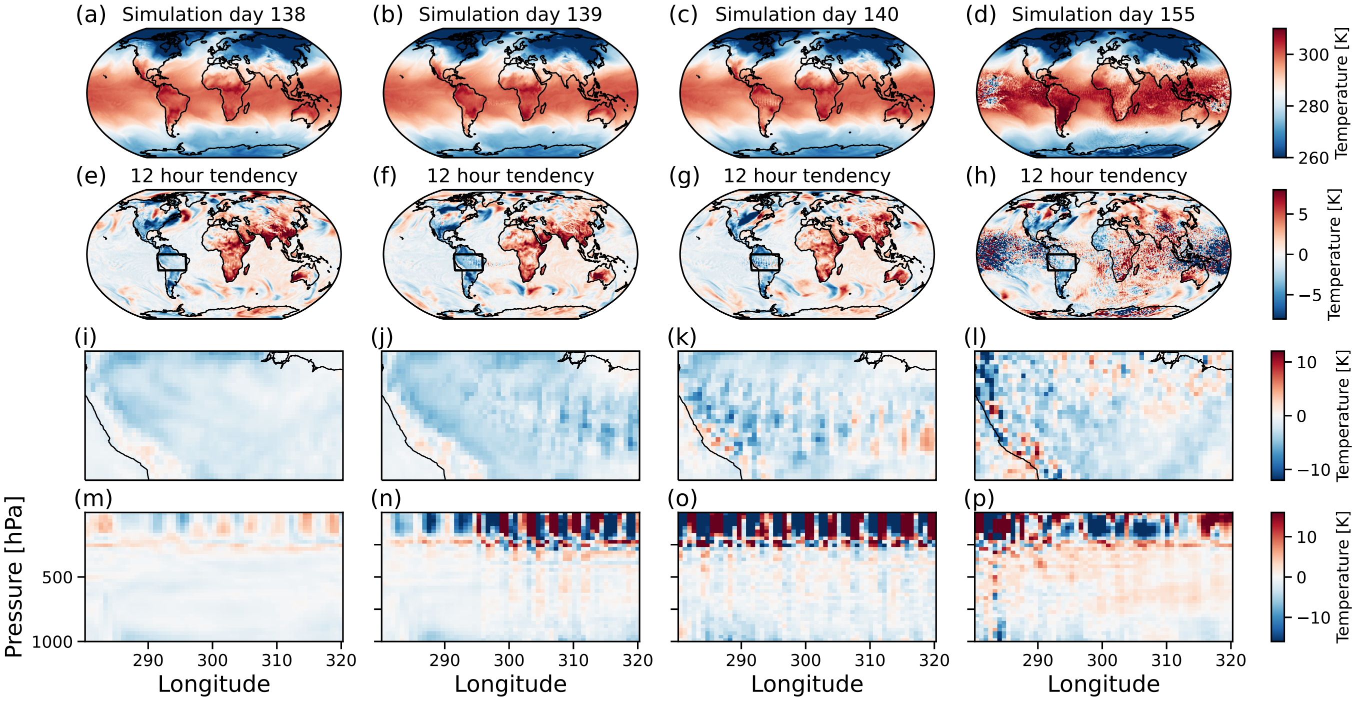

Supplementary Figure 27: Case study on instability in the NeuralGCM-0.7° model. (a-d) The temperature, (e-h) 12 hour temperature change at 1000hPa (i-l) zoom into longitudes 280 to 320 and latitudes 20S to the equator and (m-p) the vertical structure of 12 hour temperature change at latitude 10.1S, for different simulation lead times: day 138 (left column), day 139 (second column), day 140 (third column) and day 155 (right column). The simulation was initialized at 08-29-2019.

I Additional climate evaluations

I.1 Seasonal cycle

To assess the skill of our model for simulating seasonal cycles, we conduct a comprehensive comparison between NeuralGCM-1.4° resolution and ERA5. We ran 2-year simulations with 37 different initial conditions spaced at 10 days for the year 2019. Out of these 37 initial conditions, 35 successfully completed full two years without encountering model instability. The comparison is done for the year 2020, focusing on several key aspects. We begin by examining the global mean temperature at 850 hPa and find that NeuralGCM-1.4° closely resembles ERA5, both in terms of mean temperature and variability for all stable initial conditions (main text Fig.4a).

To example the tropical circulation we analyze the Hadley cell seasonal cycle and its amplitude. The Hadley cell circulation is characterized by computing the mass streamfunction $\psi(\phi, p)$, which quantifies the mass transport between latitudes and altitudes. The mass streamfunction is

$$\psi(\phi, p) = \frac{2\pi cos(\phi)}{g} \int_{p}^{p_s} \bar{v} dp, \tag{14}$$

where ϕ is the latitude, p is the pressure, g is the acceleration due to gravity, p_s is the surface pressure and $\bar{v}$ is the zonal mean meridional velocity. We find that NeuralGCM-1.4° is able to capture both the seasonal cycle and the amplitude of the Hadley cell circulation (SI Fig. 28). We also demonstrate in SI Fig. 29 that NeuralGCM-1.4° can accurately capture the spatial structure of the wind during the Indian monsoon and non-monsoon months.

To characterize the extratropical circulation, we plot zonal-mean zonal wind in different seasons and find that NeuralGCM-1.4° has a very similar structure compared to ERA5 (SI Fig. 28) with the exception that above 30hPa there are noticeable differences as NeuralGCM-1.4° does not optimize its predictions for these levels (since the upper-most level exist in sigma coordinates is ≈ 0.03). We also consider the extratropical storm tracks and we compare the seasonal cycle of the eddy kinetic energy (EKE). EKE is computed as:

$$\mathrm{EKE} = \int_{150\mathrm{hPa}}^{1000\mathrm{hPa}} \frac{1}{2\mathrm{g}} (\mathrm{u}'^2 + \mathrm{v}'^2) \mathrm{dp}, \tag{15}$$

where the prime symbol denotes deviations from the instantaneous zonal mean (i.e., $v' = v - \bar{v}$, where $\bar{v}$ represents the zonal mean of the variable), and g is the gravitational acceleration. We find that NeuralGCM-1.4° successfully captures the seasonal cycle of EKE, displaying the correct seasonal and spatial structure of EKE (SI Fig. 31).

We also illustrate the global annual cycle of atmospheric water and total kinetic energy ($\mathrm{TKE} = \int_{150\mathrm{hPa}}^{1000\mathrm{hPa}} \frac{1}{2\mathrm{g}} (\mathrm{u}^2 + \mathrm{v}^2) \mathrm{dp}$), demonstrating that there is no visible drift in these quantities and that we achieve a realistic annual cycle (SI Fig. 30). This is in contrast to a recent attempt at hybrid modelling found that the atmosphere tend to dry out rapidly [21]. The ensemble mean of NeuralGCM-1.4°'s precipitable water

accurately captures the magnitude seen in ERA5 during 2020, yielding a smaller RMSE than the climatological value. However, the ensemble mean TKE of NeuralGCM-1.4° exhibits a slight bias, trending lower than the ERA5 values.

I.2 Tropical cyclone tracking

To assess NeuralGCM's capability to simulate Tropical Cyclones (TCs), we use the TempestExtremes tracker [22] to identify TC tracks. To compare NeuralGCM TC results to ERA5 we first detect TCs in ERA5 native resolution (0.25°) using the default configuration of TempestExtremes.

The default configuration of TempestExtremes, used for the native ERA5 resolution (0.25°), utilizes sea level pressure (SLP) as the feature-tracking variable. Candidate TCs are initially identified based on SLP minima, and a closed contour criterion is applied, demanding an SLP increase of at least 2 hPa within a 5.5° Great Circle Distance (GCD) from the candidate point. Additionally, the difference between geopotential on the 300 and 500 hPa surfaces must decrease by 58.8 m^2s^{-2} within a 6.5° GCD from the candidate geopotential, taken as the maximum geopotential height difference within 1° GCD of the candidate location. These candidates are then linked over time to form TC paths. These paths have a maximum allowable distance of 8° between consecutive candidates, feature a maximum allowable gap size of 24 hours (representing time periods with no TC identification) and have a minimum trajectory length of 54 hours. For at least 10 time steps, the underlying orography has to be less than 150m, the storm formation is constrained within latitudes of $-50°$ to 50°, and the 10m wind magnitude has to exceed 10 m/s. Tracking is conducted at 6-hour output intervals for all resolutions.

One challenge with TempestExtremes is its tendency to yield significantly different numbers of Tropical Cyclones (TCs) when applied to data at varying resolutions [23]. To apply the tracker to NeuralGCM-1.4° and make meaningful comparisons with ERA5 data, we specify a set of tracking parameters that yield nearly identical TC tracks when ERA5 data is regridded to a resolution of 1.4°.

We fine-tuned these parameters so that TempestExtremes detected nearly identical TC tracks (as shown in SI Fig. 34a) and a similar number of TCs, with 88 in the native resolution (using the default parameters for tracking) and 84 in the 1.4° resolution (using the new set of parameters for tracking). The main differences is that we reduced the gradient requirements, necessitating an SLP increase of at least 0.6 hPa within a 5.5° GCD radius of the candidate node and the difference between geopotential on the 300 and 500 hPa surfaces must decrease by 25.8 m^2s^{-2} within a 6.5° GCD from the candidate geopotential, taken as the maximum geopotential height difference within 1° GCD of the candidate location. It's worth noting that, for the 1.4° tracking, we do not use the 10m wind criterion since this data was not available for the NeuralGCM simulation.

Next, we compare these results with TCs simulated by the X-SHiELD model (main text Fig.4e,f,g) when regridded to 1.4°, allowing for a fair comparison with NeuralGCM-1.4° at the same resolution. Since the X-SHiELD dataset lacks SLP information, we utilize vorticity for tracking TCs. In a manner similar to what is described in the previous paragraph, we identify a set of vorticity parameters that, when used

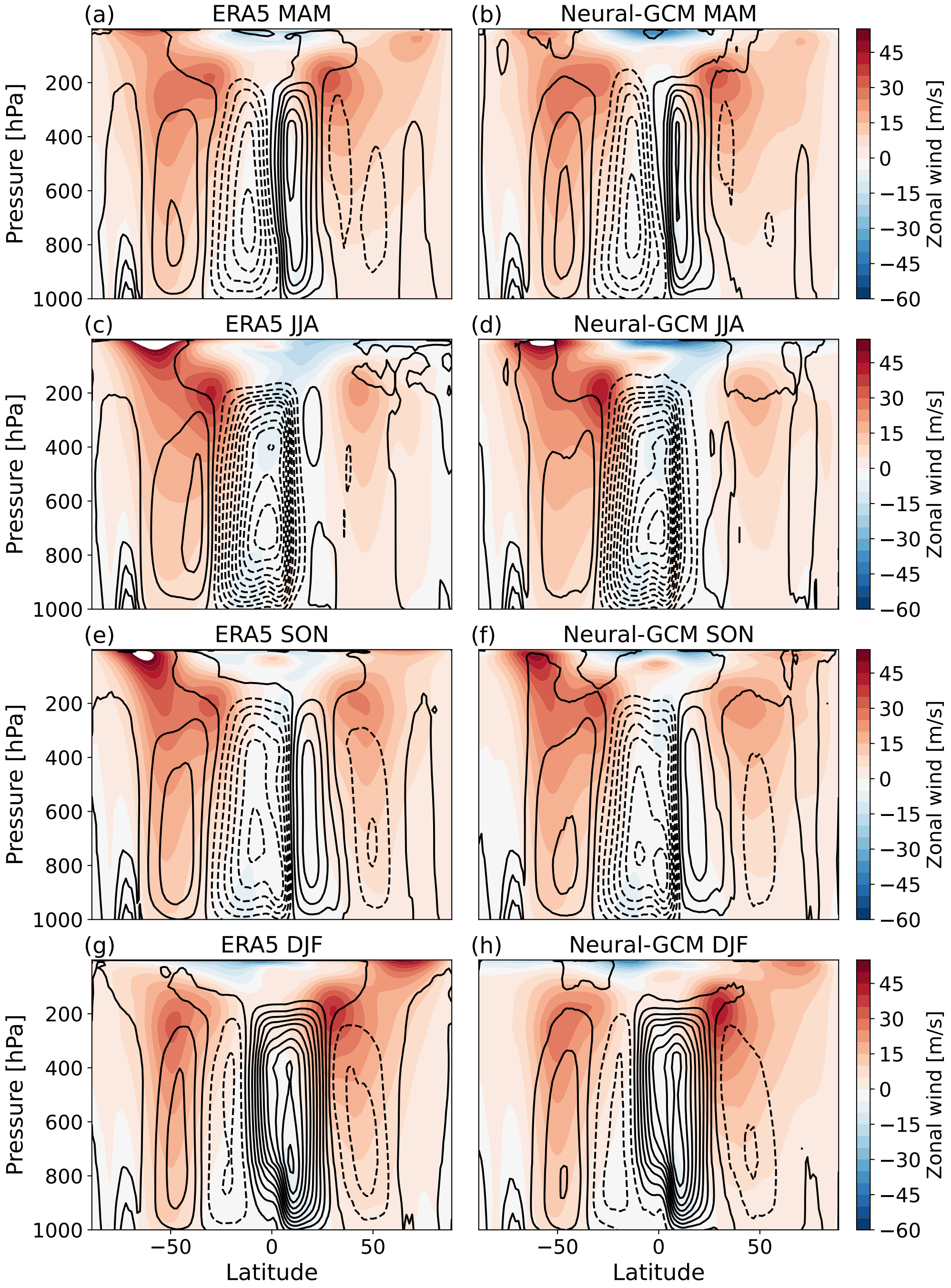

Supplementary Figure 28: The Hadley cell circulation and zonal wind for different seasons in NeuralGCM and ERA5. The mass streamfunction (contours) and zonal-mean zonal wind (colors) as a function of pressure and latitude averaged over different seasons during 2020 for (a,c,e,f) ERA5 and (b,d,f,h) NeuralGCM-1.4° simulation initialized in October 18th 2019. Solid contours indicate positive values of mass streamfunction and dashed contours indicate negative values and contour intervals are 2×10^{10} kg/s.

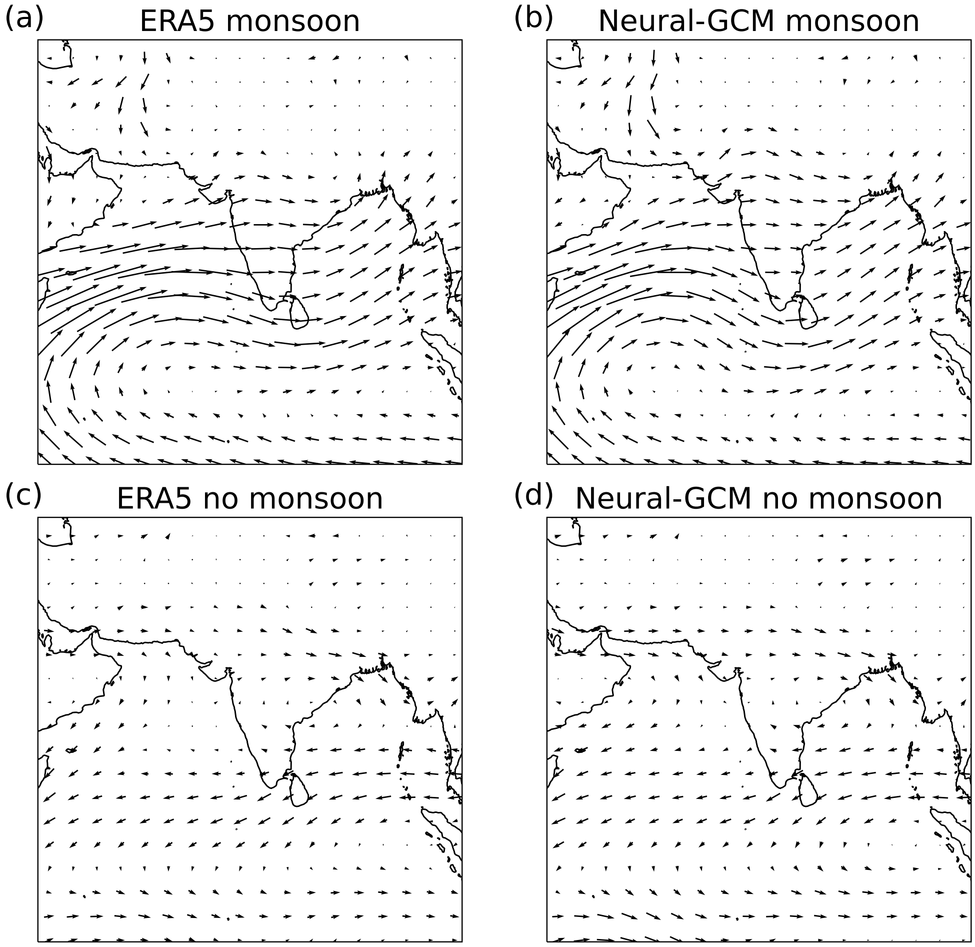

Supplementary Figure 29: Winds during Monsoon and no Monsoon months in NeuralGCM and ERA5. Quiver plot of the time mean wind at 850 hPa for the Indian Monsoon months (defined here as June 15th to Septemper 15th; panels a and b) and no Monsoon months (defined here as January 1st to April 1st; panels b and d) for 2020 for (a,c) ERA5 (b,d) NeuralGCM-1.4° simulation (initialized in October 18th 2019)

with the TempestExtremes tracker on ERA5 data regridded to 1.4° resolution, yield results that closely matched TCs in native ERA5 data tracked using SLP as the criterion. We successfully fine-tune the vorticity parameters to ensure that TempestExtremes detects nearly identical TC tracks (as shown in SI Fig. 34b) when using the vorticity criterion compared to when using the tracker with native ERA5 resolution and the SLP criterion. This yields a similar number of TCs, with 88 in the native resolution and 86 when using the vorticity criterion in the 1.4° resolution. For vorticity tracking, we require a vorticity increase/decrease of at least 0.00006 s^{-1} within a 5.5° GCD radius of the candidate location. Additionally, we impose the requirement

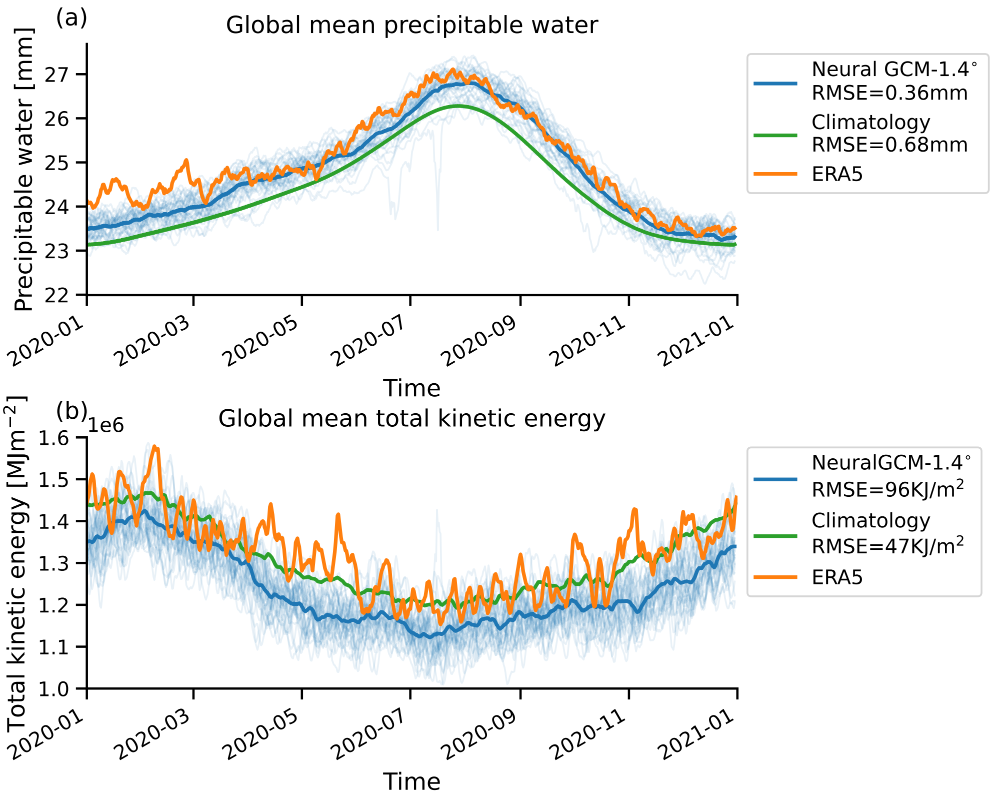

Supplementary Figure 30: Yearly cycle of precipitable water and total kinetic energy. Global mean (a) precipitable water and (b) total kinetic energy for 2020 ERA5 for 2020 (orange), climatology (defined as the averaged temperature between 1990-2019; green), and for NeuralGCM-1.4° for 2020 for simulations initialized every 10 days during 2019 (thick blue represents the ensemble mean, and thin blue lines indicate different initial conditions).

for a decrease in geopotential height difference between the 300 hPa and 500 hPa of 25.8 m^2s^{-2} within a 6.5° GCD from the candidate geopotential. (see SI Table 6).

We apply these new SLP and vorticity parameters to detect TCs in the NeuralGCM-1.4° simulation. We find that tracking TCs with both sets of parameters produces similar TC tracks (SI Fig. 34c). These TC tracks also closely resemble TC tracks identified in ERA5 data in terms of number of TCs, as well as their locations and shapes (SI Fig. 34).

We also employ the tracker to derive TC tracks from both ECMWF-ENS and NeuralGCM-ENS, each at a resolution of 1.4°. However, since ECMWF-ENS lacks data for geopotential at 300 hPa, which is necessary for the conditions we used above, we track TCs for the ensemble using the sea-level pressure (SLP) criterion in conjunction with the vorticity criterion, as previously described for simulations at the 1.4° resolution.

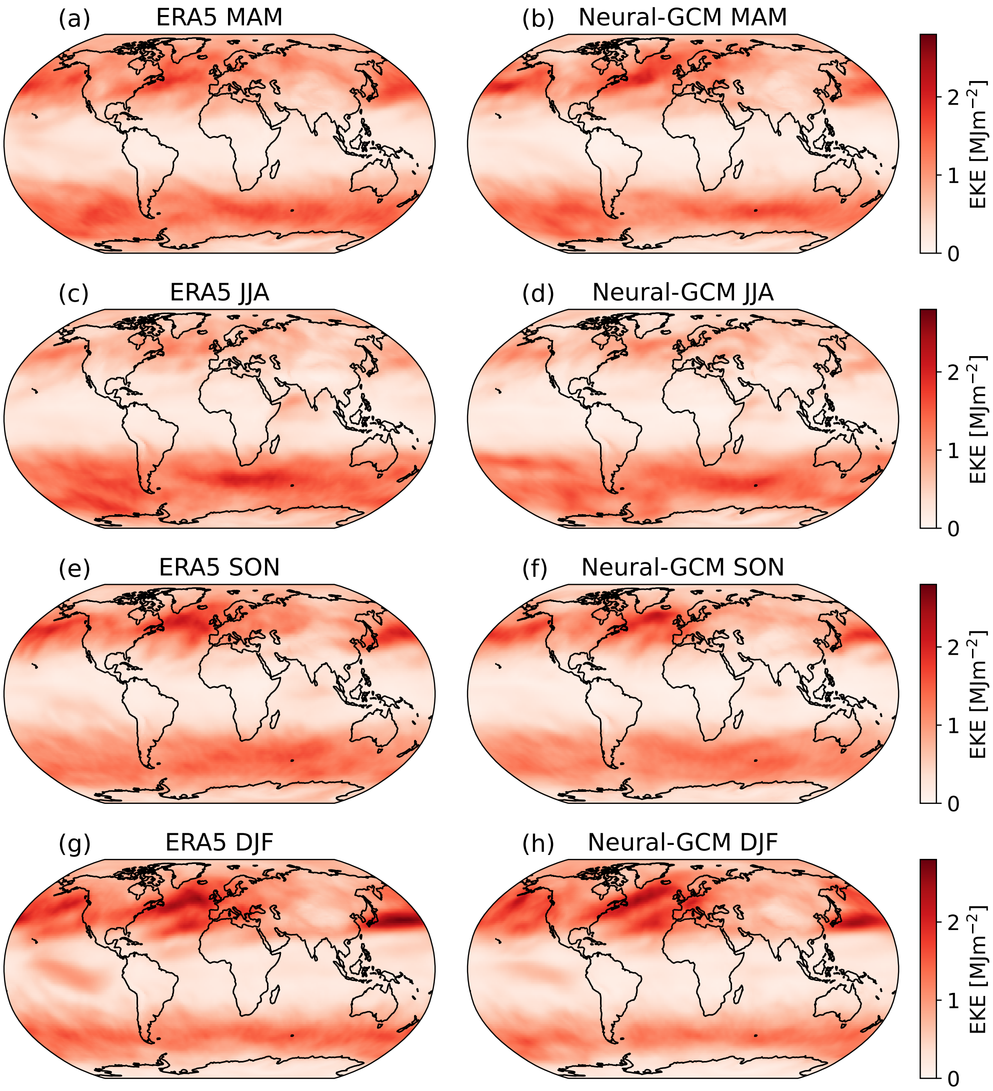

Supplementary Figure 31: Eddy kinetic energy in NeuralGCM and ERA5. Vertically integrated Eddy Kinetic Energy (EKE) as a function of longitude and latitude averaged over different seasons during 2020 for (a,c,e,f) ERA5 and (b,d,f,h) NeuralGCM-1.4° simulation (initialized in October 18th 2019).

I.3 CMIP6 models used in AMIP runs

We analyzed data from 22 CMIP6 models that were configured to use prescribed sea surface temperatures (SST), known as AMIP runs, taken from Google's Public Dataset program stored on Google Cloud Storage. Among these, for the following 17 models — BCC-CSM2-MR, CAMS-CSM1-0, CESM2, CESM2-WACCM,

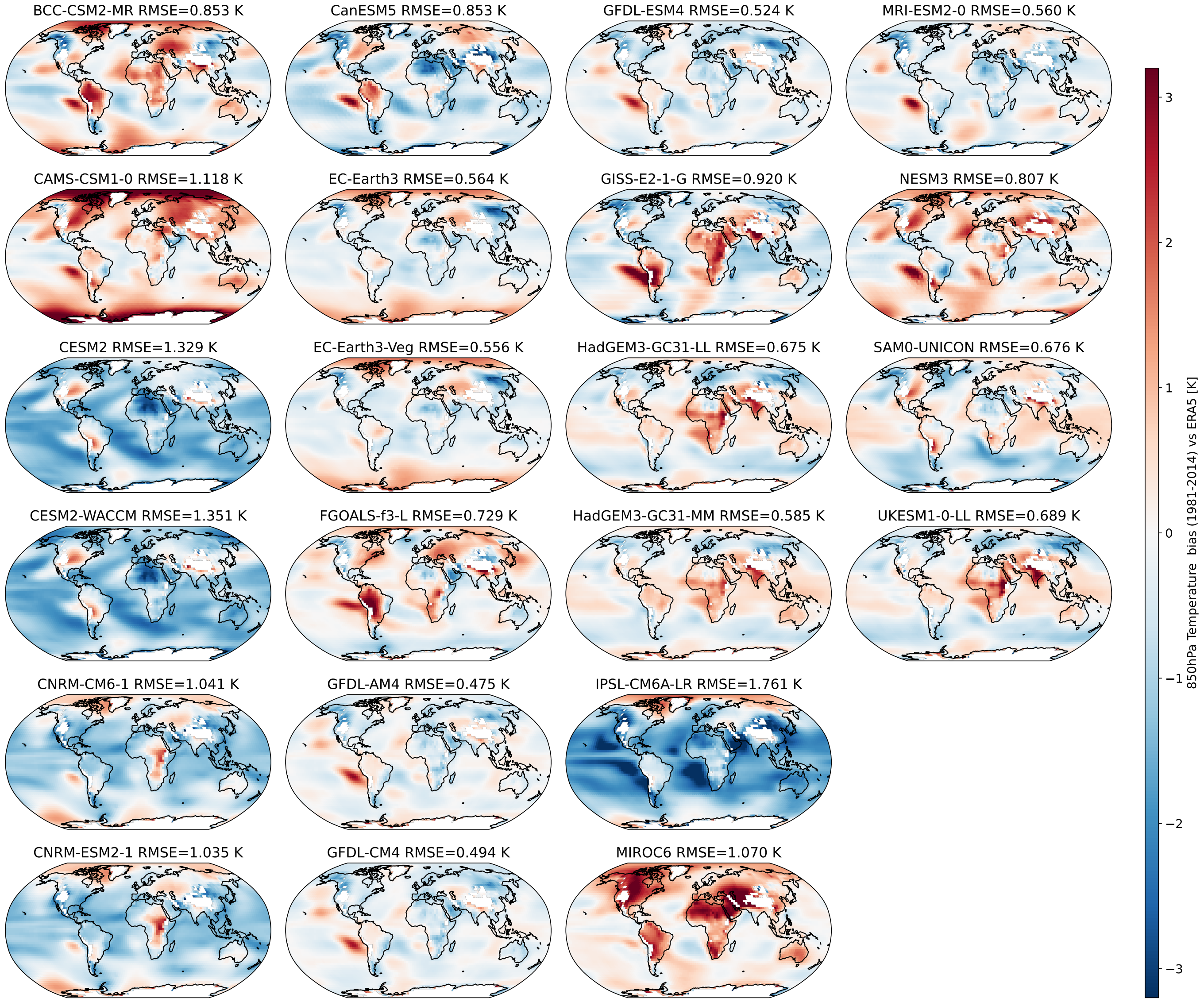

Supplementary Figure 32: 850hPa temperature bias averaged over 1981-2014 in 22 models from CMIP6 AMIP simulations.

CanESM5, EC-Earth3, EC-Earth3-Veg, FGOALS-f3-L, GFDL-AM4, GFDL-CM4, GFDL-ESM4, GISS-E2-1-G, IPSL-CM6A-LR, MIROC6, MRI-ESM2-0, NESM3, and SAM0-UNICON — we utilized the "r1i1p1f1" variant identifier to which we had access. However, for the remaining five models, we resorted to using alternative variant identifiers: "r1i1p1f2" for CNRM-CM6-1 and CNRM-ESM2-1, "r2i1p1f3" for HadGEM3-GC31-LL, "r1i1p1f3" for HadGEM3-GC31-MM, and "r1i1p1f2" for

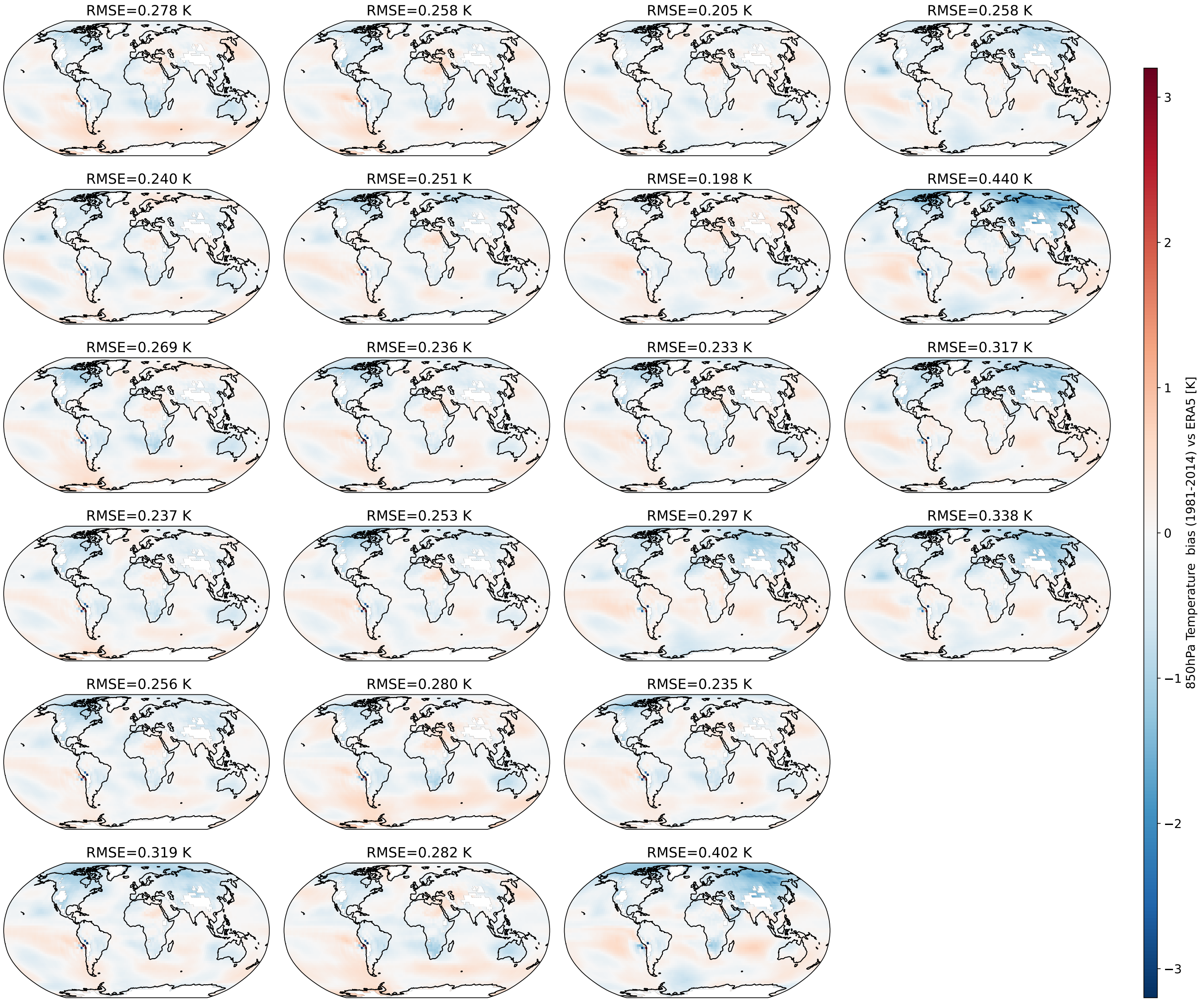

Supplementary Figure 33: 850hPa temperature bias averaged over 1981-2014 in 22 NeuralGCM-2.8° simulations.

UKESM1-0-LL. Additionally, although data from the E3SM model was available from Google's Public Dataset program, we chose not to include it in our analysis due to some temperature artifacts near Antarctica.

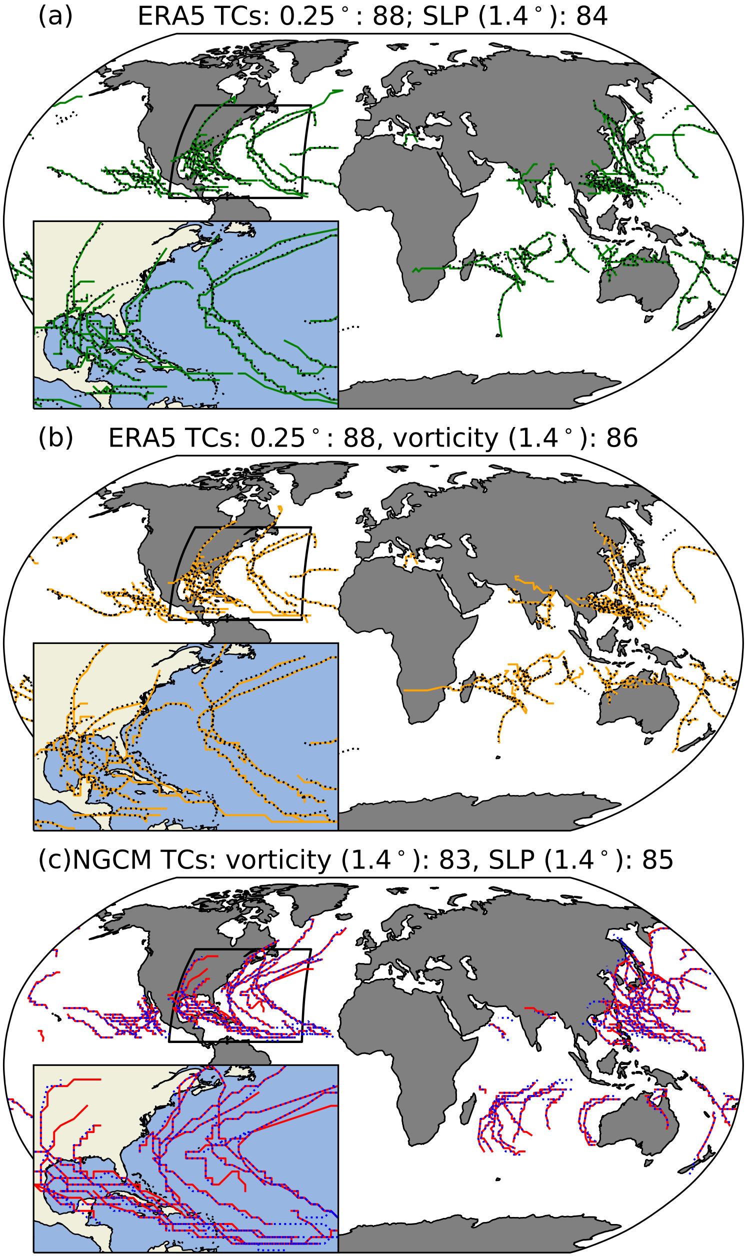

Supplementary Figure 34: Tropical cyclone tracks for different tracking criteria. Tropical Cyclone (TC) tracks identified from (a,b) ERA5 and (c) NeuralGCM-1.4° using different criteria and resolutions. (a) TC tracks from ERA5 native resolution (0.25°) using sea level pressure criterion (SLP; dotted black) and ERA5 at 1.4° resolution using SLP modified criterion (green). (b) TC tracks from ERA5 native resolution (0.25°) using SLP criterion (dotted black) and ERA5 at 1.4° resolution using vorticity criterion (orange). (c) TC tracks from NeuralGCM-1.4° using SLP modified criterion (dotted blue) and vorticity criterion (red). The TC tracking was applied to all simulations between 01-19-2020 to 01-17-2021, which were the dates that were available for the X-SHiELD model. NeuralGCM-1.4° initialized on 10-18-2019 (similar to X-SHiELD). Insets show a zoom in into the Northern Atlantic region.

	Criterion 1	Criterion 2
Sea level pressure tracking (0.25°)	SLP change of 200Pa over 5.5 GCD	Difference between geopotential surfaces at 300 and 500 hPa decrease by at least -58.8m^2/s^2 over 6.5 GCD
Sea level pressure tracking (1.4°)	SLP change of 60Pa over 5.5 GCD	Difference between geopotential surfaces at 300 and 500 hPa decrease by at least -25.8m^2/s^2 over 6.5 GCD
Vorticity tracking (1.4°)	Vorticity at 850hPa change of ±0.00006s^{-1} over 5.5GCD	Difference between geopotential surfaces at 300 and 500 hPa decrease by at least -25.8m^2/s^2 over 6.5 GCD
SLP ensemble tracking (1.4°)	SLP change of 60Pa over 5.5 GCD	Vorticity at 850hPa change of ±0.00006s^{-1} over 5.5GCD

Supplementary Table 6: The parameters used for TC tracking when using different model resolutions.

I.4 Generalizing to unseen data

One motivation for incorporating strong physics priors into NeuralGCM is to improve performance on out-of-sample data, such as that caused by climate change or systematic changes in Earth observing systems. Physically consistent weather models should remain accurate even under different climates or with weather conditions outside their training data. First, we study the ability of NeuralGCM models to generalize weather forecasts to warmer years. Later, we investigate its ability to simulate warmer climates.

I.4.1 Weather forecasting in warmer years

To assess the extrapolation capabilities of NeuralGCM in the context of weather predictions, we trained a NeuralGCM-2.8° model using ERA5 data exclusively from 1979 to 2000. We then tested its performance across 21 future years. SI Fig. 35 demonstrates the model's skill in mid-range weather prediction for various years and variables. For geopotential, forecast skill remains remarkably consistent even when extrapolating 20 years into the future. While there is a noticeable decline in the 4-day forecast accuracy for temperature and specific humidity, skill remains stable for temperature and exhibits only a slight deterioration for specific humidity in longer-range predictions.

Next, we wanted to compare the ability of NeuralGCM-0.7° to generalize to future years against that of GraphCast. Because the final version of NeuralGCM-0.7° was trained on data through 2019, to produce additional data for comparisons we also consider a developmental version NeuralGCM-0.7°-2017 only trained through 2017 data. This model uses the same architecture described previously with three major differences:

1. It does not include a surface embedding or surface forcing features (sea surface temperature, sea ice concentration).
2. It does not include the memory feature.
3. Its loss is scaled differently in time, like $(1+(\tau/24))^{-1}$ instead of $(1+(\tau/40)^4)^{-1/2}$.

In SI Fig. 36 we compare accuracy trends of GraphCast, NeuralGCM-0.7°, NeuralGCM-0.7°-2017 and ECMWF-HRES evaluated on ERA5 data from years 2018-2022. In this experiment GraphCast and NeuralGCM-0.7°-2017 variants were trained

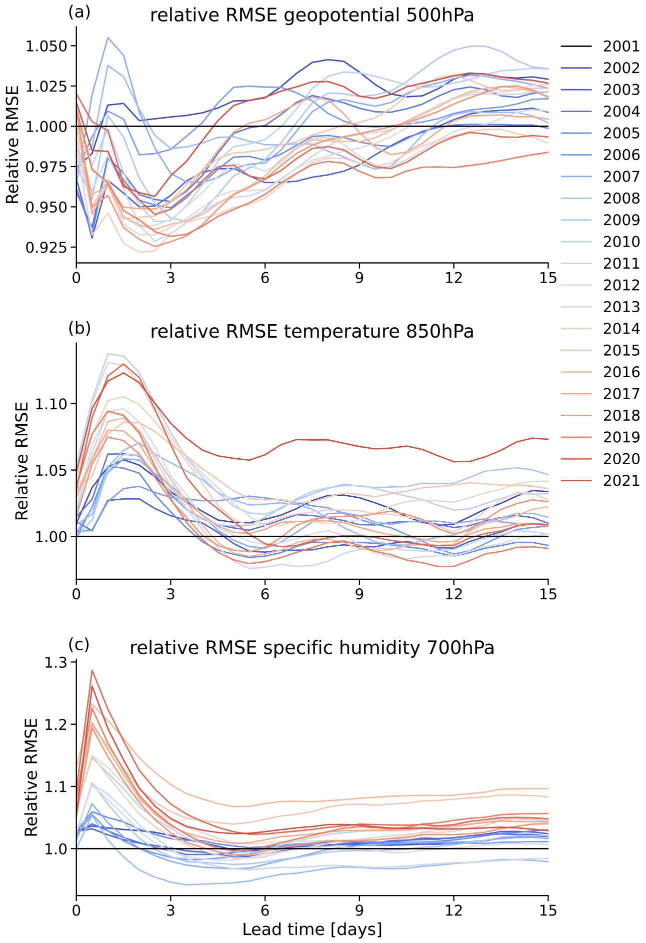

Supplementary Figure 35: Extrapolation of mid-range weather forecast skill over 21 years. Relative Root Mean Square Error (RMSE), normalized to the year 2001, for a NeuralGCM-2.8° model trained on 1979-2000 ERA5 data. Results are shown for (a) 500 hPa geopotential height, (b) 850 hPa temperature, and (c) 700 hPa specific humidity as a function of forecast lead time.

on data until 2017. ECMWF-HRES and NeuralGCM models display little variation over evaluation years, while GraphCast accuracy degrades by a few percent when evaluated on 2022 forecasts (5 years more recent than the training domain), consistent with the degraded performance noted in the GraphCast paper. Forecasts of geopotential and at short lead times have the largest increases in error.

I.4.2 Extrapolation to warmer climates

All NeuralGCM models have been trained solely on ERA5 data. As with all machine learning models, we do not expect the NeuralGCM models to extrapolate beyond their

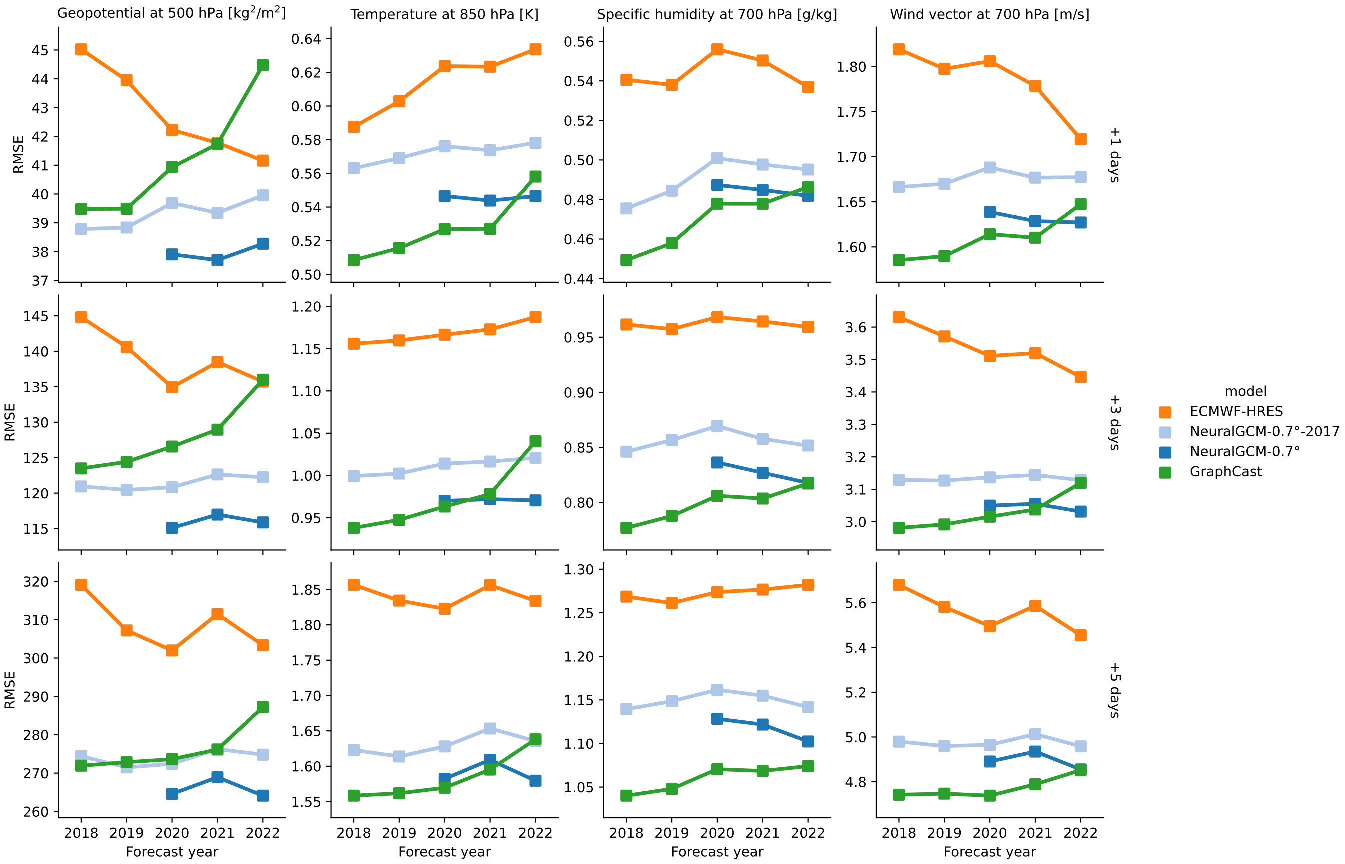

Supplementary Figure 36: RMSE for 1-, 3- and 5-day forecasts, starting in different forecast outside the training datasets for NeuralGCM and GraphCast. RMSE is averaged over forecasts initialized every 12 hours at midnight and noon UTC in the indicated year.

training regime. However, we acknowledge that extrapolation might be reasonable in cases where the input/output distributions do not substantially change compared to the training data. Therefore, we aimed to test whether NeuralGCM can still provide reasonable results for climates that are moderately to substantially warmer.

To assess the capability of NeuralGCM to simulate warmer climates, we conducted AMIP-like simulations with warmer SST (including +1K, +2K, and +4K SST) over a duration of 34 years using NeuralGCM-2.8°. As a baseline for comparison, we used AMIP and AMIP +4K SST runs of the CESM model (CESM AMIP +4K was available until 2013). Out of 8 different runs with initial conditions spaced every 50 days during the year 1980, 6 (for AMIP +1K), 6 (for AMIP +2K), and 4 (for AMIP +4K) simulations were stable for the entire 34-year period. Since we compare to a single realization of CESM, we have chosen to use simulations that were initialized on April

10th, as simulations started on this date remained stable for 34 years across all SST warming levels.

In SI Fig. 37, we test the response of NeuralGCM model runs to SST warming. We find that the zonal mean temperature structure and the zonal mean zonal wind response in NeuralGCM for +1K and +2K SST warming runs are broadly consistent with the CESM response. Specifically, robust features of climate warming, such as upper tropospheric warming, polar amplification, and Southern Hemisphere polar jet shift [24], are present in all simulations. However, there are also some differences in the responses, for example, the zonal mean zonal wind changes in the tropics have opposite signs in CESM and NeuralGCM. We find that the NeuralGCM +4K SST warming simulation shows a very different response compared to NeuralGCM simulations with +1K and +2K SST warming. This likely indicates that when using NeuralGCM in a climate driven by +4K SST warming, the results provided by NeuralGCM are unrealistic.

SI Fig. 38a demonstrates that the mean global temperature at 850hPa adjusts in response to an increase in SST by +4K on the same timescale in both NeuralGCM and MIROC6. We use MIROC6 for comparison because the daily temperature data for both AMIP and AMIP +4K SST scenarios were available from Google Cloud Storage for MIROC6. This implies that the short-term response of NeuralGCM is similar to that of a physics-based model.

In SI Fig. 38b and c, we present the global mean temperature at 850hPa for NeuralGCM and CESM simulations. The most notable observation is that, unlike CESM simulations, NeuralGCM simulations with warmer SST do not exhibit the same trend as NeuralGCM with the AMIP configuration. This suggests that NeuralGCM, when simulating a warmer climate, experiences a climate drift. The climate drift is particularly evident for the +4K SST warming (a scenario we have already noted NeuralGCM does not realistically extrapolate to), but it is also observable in the +1K and +2K SST warming scenarios, albeit to a lesser extent. For example, after 34 years of model integration, the temperature difference between the AMIP run and AMIP + 1K SST -1K is approximately 0.25K. We note that the drift we find in NeuralGCM AMIP +1K and +2K SST warming is substantially lower than the warming itself. Therefore, we believe it is still appropriate to compare the response to warming in SI Fig. 38.

In the future, NeuralGCM could improve its ability to generalize to different climates, even those it has not trained on, by using a mix of strategies. While climate-invariant methods [25] can help, training NeuralGCM on data obtained from simulations (e.g., of warmer climates) and incorporating physically-based models (e.g., radiation schemes) will likely be necessary.

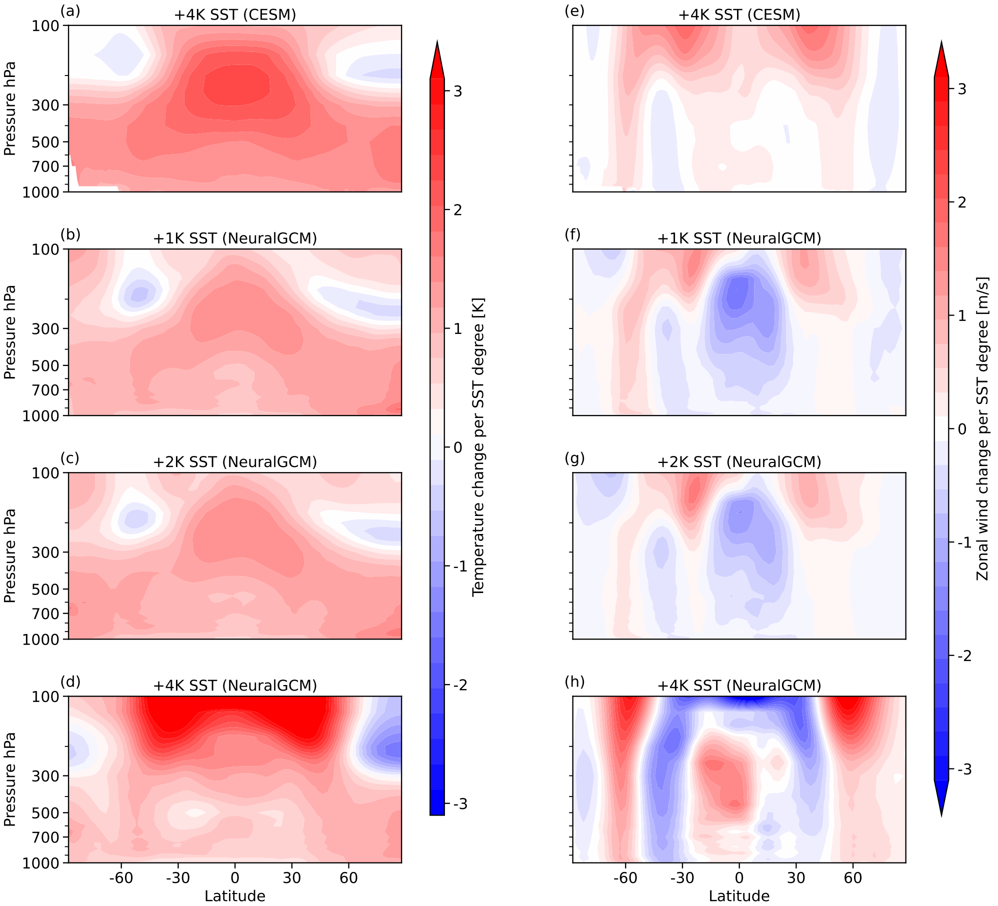

Supplementary Figure 37: Response of temperature and zonal wind to sea surface temperature (SST) warming. Left panels depict the zonal mean temperature response per degree of SST increase as a function of latitude and pressure, calculated as the difference between AMIP runs with increased SST to AMIP simulations averaged over 1981-2013: (a) CESM with a +4K SST warming, (b-d) NeuralGCM with +1K, +2K, and +4K SST warming, respectively. Right panels illustrate the corresponding zonal mean zonal wind response per SST degree increase for (e) CESM with a +4K SST enhancement and (f-h) NeuralGCM with +1K, +2K, and +4K SST warming, respectively. All NeuralGCM models used initial condition from April 10th 1980.

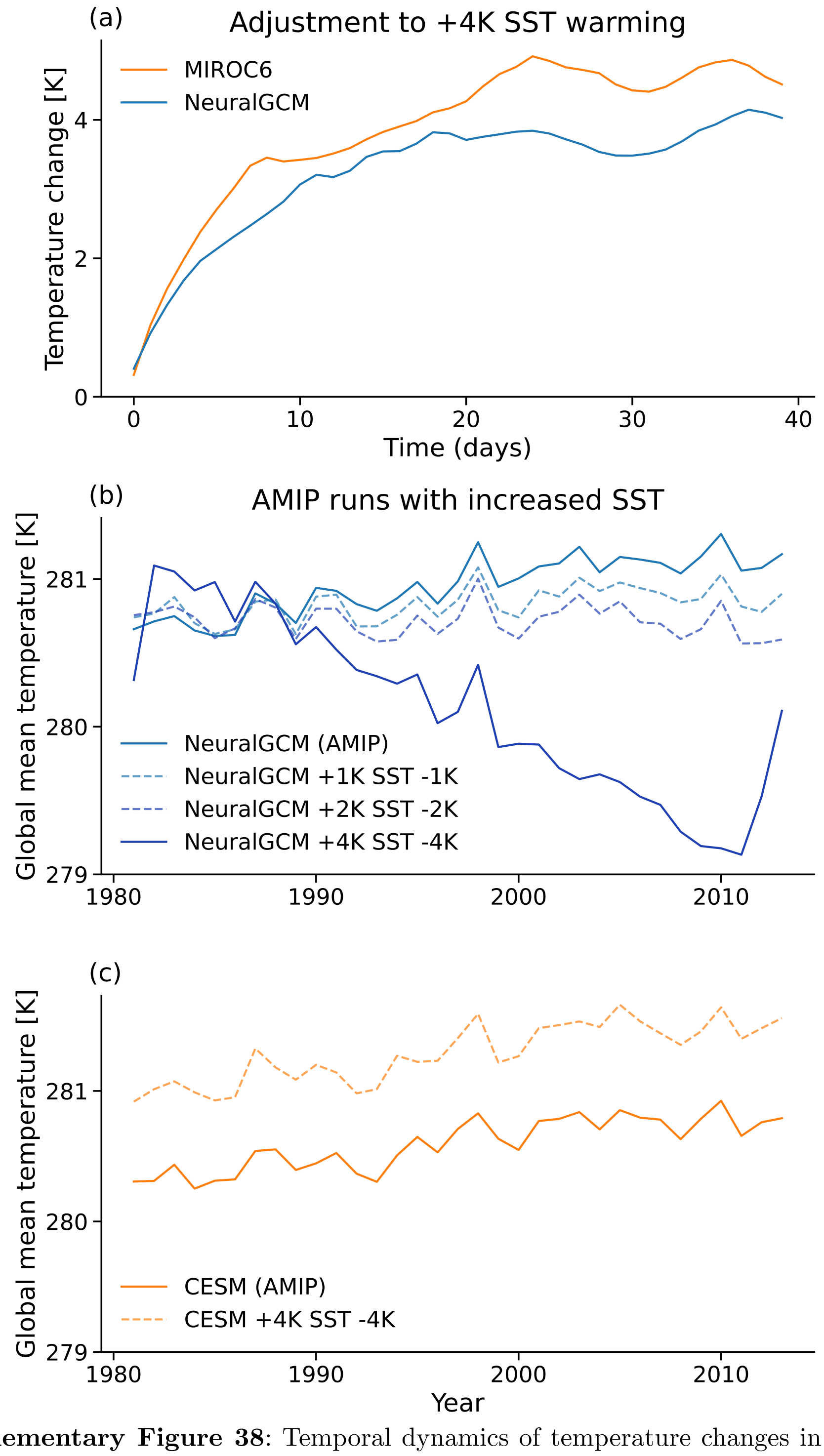

Supplementary Figure 38: Temporal dynamics of temperature changes in AMIP simulations with warmed sea surface temperature (SST). Panel (a) shows the 40-day 850hPa global temperature response to a +4K SST increase in MIROC6 (defined as the difference between AMIP +4K and the standard AMIP run; represented in orange) and NeuralGCM (in blue). Panel (b) presents the global mean temperature at 850hPa across various NeuralGCM AMIP scenarios, including standard runs and those with +1K, +2K, and +4K SST enhancements, adjusting the global mean by subtracting the corresponding SST warming. Panel (c) displays the global mean temperature at 850hPa for both the CESM AMIP standard run and the CESM AMIP scenario with a +4K SST increase, applying the same SST adjustment methodology. Panels b and c cover the period from 1981 to 2013, with 2013 being the final year for which CESM AMIP +4K SST data were available. All NeuralGCM simulations commenced from the initial condition on April 10th, 1980.

References

[1] Lam, R. *et al.* Learning skillful medium-range global weather forecasting. *Science* **382**, 1416–1421 (2023). URL https://www.science.org/doi/abs/10.1126/science.adi2336.

[2] Bourke, W. A Multi-Level Spectral Model. I. Formulation and Hemispheric Integrations. *Mon. Weather Rev.* **102**, 687–701 (1974). URL https://journals.ametsoc.org/view/journals/mwre/102/10/1520-0493_1974_102_0687_amlsmi_2_0_co_2.xml.

[3] Durran, D. R. *Numerical methods for fluid dynamics: With applications to geophysics* Second edn, Vol. 32 (Springer, New York, 2010).

[4] Jouppi, N. *et al. Tpu v4: An optically reconfigurable supercomputer for machine learning with hardware support for embeddings*, ISCA '23 (Association for Computing Machinery, New York, NY, USA, 2023). URL https://doi.org/10.1145/3579371.3589350.

[5] Henry, G., Tang, P. T. P. & Heinecke, A. *Leveraging the bfloat16 artificial intelligence datatype for higher-precision computations* (IEEE, Kyoto, Japan, 2019). URL https://ieeexplore.ieee.org/document/8877427/.

[6] Xu, Y. *et al.* GSPMD: General and scalable parallelization for ML computation graphs (2021). URL http://arxiv.org/abs/2105.04663.

[7] Douglas, S., Vikram, S., Bradbury, J., Zhang, Q. & Johnson, M. shmap (shard_map) for simple per-device code (2023). URL https://jax.readthedocs.io/en/latest/jep/14273-shard-map.html. Accessed: 2023-10-21.

[8] Palmer, T. N. *et al.* Stochastic parametrization and model uncertainty (2009). URL https://www.ecmwf.int/sites/default/files/elibrary/2009/11577-stochastic-parametrization-and-model-uncertainty.pdf.

[9] Battaglia, P. W. *et al.* Relational inductive biases, deep learning, and graph networks. *arXiv preprint arXiv:1806.01261* (2018).

[10] Whitaker, J. S. & Kar, S. K. Implicit–explicit runge–kutta methods for fast–slow wave problems. *Monthly weather review* **141**, 3426–3434 (2013).

[11] Keisler, R. Forecasting global weather with graph neural networks. *arXiv preprint arXiv:2202.07575* (2022).

[12] Jablonowski, C. & Williamson, D. L. The pros and cons of diffusion, filters and fixers in atmospheric general circulation models. *Numerical techniques for global atmospheric models* 381–493 (2011).

[13] Jablonowski, C. & Williamson, D. L. in *The pros and cons of diffusion, filters and fixers in atmospheric general circulation models* (eds Lauritzen, P., Jablonowski, C., Taylor, M. & Nair, R.) *Numerical Techniques for Global Atmospheric Models* 381–493 (Springer Berlin Heidelberg, Berlin, Heidelberg, 2011). URL https://doi.org/10.1007/978-3-642-11640-7_13.

[14] Rasp, S. *et al.* WeatherBench 2: A benchmark for the next generation of data-driven global weather models (2023). Preprint at http://arxiv.org/abs/2308.15560.

[15] Gneiting, T. & Raftery, A. E. Strictly Proper Scoring Rules, Prediction, and Estimation. *J. Am. Stat. Assoc.* **102**, 359–378 (2007). URL https://doi.org/10.1198/016214506000001437.

[16] Kingma, D. P. & Ba, J. Adam: A method for stochastic optimization. *arXiv preprint arXiv:1412.6980* (2014).

[17] Gilleland, E., Ahijevych, D., Brown, B. G., Casati, B. & Ebert, E. E. Intercomparison of spatial forecast verification methods. *Weather and forecasting* **24**, 1416–1430 (2009).

[18] Bonavita, M. On the limitations of data-driven weather forecasting models. *arXiv preprint arXiv:2309.08473* (2023).

[19] May, R. M. *et al.* Metpy: A meteorological python library for data analysis and visualization. *Bulletin of the American Meteorological Society* **103**, E2273 – E2284 (2022). URL https://journals.ametsoc.org/view/journals/bams/103/10/BAMS-D-21-0125.1.xml.

[20] Bi, K. *et al.* Accurate medium-range global weather forecasting with 3d neural networks. *Nature* **619**, 533–538 (2023).

[21] Kwa, A. *et al.* Machine-learned climate model corrections from a global storm-resolving model: Performance across the annual cycle. *Journal of Advances in Modeling Earth Systems* **15**, e2022MS003400 (2023).

[22] Ullrich, P. A. *et al.* Tempestextremes v2. 1: A community framework for feature detection, tracking, and analysis in large datasets. *Geoscientific Model Development* **14**, 5023–5048 (2021).

[23] Roberts, M. J. *et al.* Impact of model resolution on tropical cyclone simulation using the highresmip–primavera multimodel ensemble. *Journal of Climate* **33**, 2557–2583 (2020).

[24] Vallis, G. K., Zurita-Gotor, P., Cairns, C. & Kidston, J. Response of the large-scale structure of the atmosphere to global warming. *Quarterly Journal of the Royal Meteorological Society* **141**, 1479–1501 (2015).

[25] Beucler, T. *et al.* Climate-invariant machine learning. *Science Advances* **10**, eadj7250 (2024).
